# Polyphyletic origin of the genus *Physarum* (Physarales, Myxomycetes) revealed by nuclear rDNA mini-chromosome analysis and group I intron synapomorphy

**DOI:** 10.1186/1471-2148-12-166

**Published:** 2012-08-31

**Authors:** Satish CR Nandipati, Kari Haugli, Dag H Coucheron, Edward F Haskins, Steinar D Johansen

**Affiliations:** 1 RNA and Transcriptomics group, Department of Medical Biology, Faculty of Health Sciences, University of Tromsø, MH-building Breivika, N-9037, Tromsø, Norway; 2 Department of Biology, University of Washington, Seattle, Washington, USA

## Abstract

**Background:**

Physarales represents the largest taxonomic order among the plasmodial slime molds (myxomycetes). Physarales is of particular interest since the two best-studied myxomycete species, *Physarum polycephalum* and *Didymium iridis*, belong to this order and are currently subjected to whole genome and transcriptome analyses. Here we report molecular phylogeny based on ribosomal DNA (rDNA) sequences that includes 57 Physarales isolates.

**Results:**

The Physarales nuclear rDNA sequences were found to be loaded with 222 autocatalytic group I introns, which may complicate correct alignments and subsequent phylogenetic tree constructions. Phylogenetic analysis of rDNA sequences depleted of introns confirmed monophyly of the Physarales families Didymiaceae and Physaraceae. Whereas good correlation was noted between phylogeny and taxonomy among the Didymiaceae isolates, significant deviations were seen in Physaraceae. The largest genus, *Physarum*, was found to be polyphyletic consisting of at least three well supported clades. A synapomorphy, located at the highly conserved G-binding site of L2449 group I intron ribozymes further supported the *Physarum* clades.

**Conclusions:**

Our results provide molecular relationship of Physarales genera, species, and isolates. This information is important in further interpretations of comparative genomics nd transcriptomics. In addition, the result supports a polyphyletic origin of the genus *Physarum* and calls for a reevaluation of current taxonomy.

## Background

Myxomycetes (plasmodial slime molds) are eukaryotic microorganisms that according to Olive [1] represent one of four main groups of slime molds (Mycetozoans). The myxomycetes consist of about 850 assigned species classified into five orders (Physarales, Stemonitales, Trichiales, Liceales and Echinosteliales) [2]. A typical myxomycete species has a complex sexual life cycle that consists of two vegetative stages; a haploid unicellular stage (amoeba/ flagellate) and a diploid syncytium stage (plasmodium), as well as several dormant and developmental stages [3-5]. Myxomycetes are commonly found in nature on decaying plant materials where they feed on a variety of bacteria and unicellular eukaryotes as well as dissolved plant nutrients [5-8]. Identification of species is mainly based on morphological characters including fruiting body structures and sporocarp colors, and lime deposition [6,9]. More recently, nuclear DNA sequence markers have contributed in resolving relationships among and within taxonomic groups. The ribosomal DNA (rDNA) spacers [10], obligatory group I introns [11], ribosomal RNA (rRNA) genes [11-16], and the elongation factor-1α (EF-1α) gene [17] and have all been applied.

The order Physarales is of special interest since it contains the two best-studied myxomycete species at the biochemical and molecular levels (*Physarum polycephalum* and *Didymium iridis*) [6,18]. One unusual feature among the Physarales is the linear multicopy nature of the nuclear rDNA minichromosome, which ranges in size from about 20 kb to 80 kb among species [19-22]. Whereas the rDNA minichromosome in *D. iridis* is only 21 kb and contains a single pre-rRNA transcription unit, a 60-kb palindromic rDNA with two transcription units has been characterized in *P. polycephalum*. A hallmark of Physarales rDNA minichromosomes is the presence of multiple group I introns within the rRNA coding regions. All isolates investigated to date contain at least two group I introns in the large subunit (LSU) rRNA gene, and high intron loads are exemplified in isolates of *Fuligo septica* and *Diderma niveum* which contain 12 and 21 intron insertions, respectively [11,22-25], our unpublished results. Group I introns code for ribozymes (catalytic RNAs) that perform self-splicing by a common molecular mechanism based on a series of transesterification reactions [25]. A group I ribozyme is organized into a well-defined and highly conserved RNA core structure that consists of three helical stacks referred to as the catalytic domain (P3, P7-P9), the substrate domain (P1, P2, P10), and the scaffold domain (P4-P6) [26].

Here we report the presence of 222 rDNA group I introns and molecular phylogeny of 57 Physarales isolates (2 families, 10 genera, 31 defined species) based on combined data sets of nuclear small subunit (SSU) and LSU rRNA gene sequences. The analysis supports a polyphyletic origin of the *Physarum* genus. Phylogenetic analysis of Physarales is of particular importance since two representative species are currently under whole genome and transcriptome analyses. Whereas *P. polycephalum* is sequenced at The Genome Institute – Washington University, our laboratory at University of Tromsø investigates *D. iridis* by deep sequencing technologies. Resolving relationships of genera or species within and between the two Physarales families (Physaraceae and Didymiaceae) are crucial in interpretations of comparative genome and transcriptome data.

## Results and discussion

### High load of group I introns in myxomycete rDNA

A characteristic feature of myxomycete rRNA gene sequences is the high load of autocatalytic group I introns [11,12,24,25,27,28]. These introns have to be correctly identified by structural characterizations and removed from coding sequences prior to phylogenetic analysis. Myxomycete group I introns have been found at 23 different, but highly conserved, insertion sites [25], and several of the introns are diverse in sequence and highly complex in organization due to internal protein coding genes, large arrays of direct repeat motifs, and even additional catalytic RNA domains [11,29].

**Figure 1:**
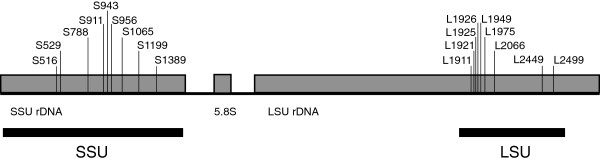
**Schematic presentation of the analyzed rDNA regions in myxomycetes.** The SSU rRNA and LSU rRNA gene regions used in phylogenetic reconstructions correspond to approximately 1800 nt and 750 nt, respectively. Myxomycete SSU and LSU rRNA genes are frequently interrupted by group I introns in myxomycetes. The position of each intron insertion site in analyzed regions is indicated and numbered according to the *E.coli* rRNA gene [25].

The rDNA sequences included in this study (near complete SSU rRNA gene and partial LSU rRNA gene; Figure 1) were examined for the presence of group I introns. We observed a massive intron contents (231 group I introns in 81 isolates; Additional file 1: Table S1, Additional file 2: Table S2, and Additional file 3: Table S3). Myxomycetes belonging to the order Physarales (Physaraceae and Didymiaceae families) harbored the majority of intron insertions (222 introns). Here, 55 and 167 introns were recognized in 21 and 36 isolates of Physaraceae and Didymiaceae, respectively (Additional file 3: Table S2 and Additional file 4: Table S3). Interestingly, all Physarales isolates investigated contained two obligatory group I introns at positions L1949 and L2449 in the LSU rRNA gene. Introns at these sites have been found to be strictly vertically inherited [11], have probably gained an essential biological role in benefit for the host cell [25], and possess highly complex structure and organization features at the RNA and DNA levels (see below).

### Relationships among the mycetozoan rDNA

According to Olive [1] the mycetozoans have been divided into four main groups of slime molds; the myxomycetes, dictyostelids, protostelids and acrasids. As an initial approach to investigate the relationships among the mycetozoans groups we performed molecular phylogeny based on unambiguously aligned SSU rRNA genes sequences (1028 nt positions) from 26 defined species representing the four mycetozoan groups as well as various amoebozoans (Table 1). These sequences were either retrieved directly from the sequence database (http://www.ncbi.nlm.nih.gov) or generated in our laboratory by PCR amplification using specific primers followed by Sanger sequence determination (see Methods).

**Table 1:** Mycetozoan and representative amoebozoan isolates

**Species**	**Isolate**^**(a)**^	**SSU**^**(b)**^	**Accession no**^**(c)**^
*MYCETOZOA*^(d)^			
**Myxomycetes** (plasmodial slime molds)			
**Physarales (Order)**			
*Didymium iridis*	Pan2	+	AJ938153
*Physarum polycephalum*	Wis1	+	X13160
**Stemonitales (Order)**			
*Comatricha nigricapillitia*	AMFD114	+	AY643824
*Stemonites flavogenita*	ATCC24714	+	HE655085
**Trichiales (Order)**			
*Arcyria stipata*	AMFD257	+	EF513170
*Trichia persimilis*	--	+	AY643826
**Liceales (Order)**			
*Cribraria cancellata*	AMFD94	+	EF513177
**Echinosteliales (Order)**			
*Echinostelium minutum*	ATCC22345	+	HE655087
**Protostelids**			
*Soliformovum irregulare*	ATCC26826	+	HE655088
**Dictyostelids** (cellular slime molds)			
*Acytostelium ellipticum*	ATCC22247	+	HE655086
*Acytostelium leptosomum*	FG12	+	AM168111
*Acytostelium subglobosum*	LB1	+	AM168110
*Dictyostelium discoideum*	--	+	K02641
*Dictyostelium fasciculatum*	SH3	+	AM168087
*Dictyostelium medusoides*	OH592	+	AM168088
*Dictyostelium rhizopodium*	AusKY-4	+	AM168063
**Acrasids**			
*Acrasis rosea*	T-235	+	AF011458
*AMOEBOZOA*			
*Amoeba leningradensis*	CCAP1503/6	+	AJ314605
*Acanthamoeba palestinensis*	CCAP1547/1	+	L09599
*Entamoeba histolytica*	HM1-IMSS	+	X65163
*Filamoeba nolandi*	ATCC50430	+	AF293896
*Gephyramoeba* sp.	ATCC50654	+	AF293897
*Hartmannella abertawensis*	Page180	+	DQ190241
*Mastigella commutans*	--	+	AF421219
*Naegleria gruberi*	NEG-M	+	AB298288
*Platyamoeba placida*	--	+	AY294150

^(a)^ Source of the organism (see Additional file 8: Table S4).

^(b)^ SSU rDNA sequence.

^(c)^ GenBank/EMBL/DDJB accession numbers.

^(d)^ Species are grouped into phyla of Protist.

--, no isolate name given; +, analysed.

Some significant findings are noted from the un-rooted maximum likelihood (ML) tree (Additional file 1: Figure S1). The dictyostelids and myxomycetes were both found to represent monophyletic groups supported by high bootstrap values for ML, maximum parsimony (MP), neighbour joining (NJ), and Bayesian inference (BAY) analyses. Furthermore, each of the five main myxomycete orders (Physarales, Stemonitales, Trichiales, Liceales and Echinosteliales) represented distinct groups within the plasmodial slime molds. These findings corroborate earlier studies on the phylogeny of slime molds [13-16,30,31]. Finally, *Soliformovum irregulare* (protostelid) and *Acrasis rosea* (acrasid) appeared both distantly related from each other and from the dictyostelids and myxomycetes. From this analysis we conclude that the apparent phenotypic similarity between the mycetozoan groups [1] is not reflected in the genetic relationship.

### Relationships within the Didymiaceae

Data presented in Additional file 1: Figure S1 and previous studies [13-16] supported a monophyletic origin of the order Physarales among the myxomycetes. To gain deeper insights in the relationships among families, genera, species and isolates within the order Physarales we performed molecular phylogeny based on two different data sets of nuclear rRNA gene sequences (Figure 1). The first data set represents an alignment of 2522 nt positions consisting of a near complete SSU rRNA gene in combination with a segment of the LSU rRNA gene (SSU/LSU data set; Additional file 5: Figure S2). The second data set consists of the LSU rRNA gene segment only (765 bp LSU data set; Additional file 6: Figure S3 and Additional file 7: Figure S4).

**Figure 2:**
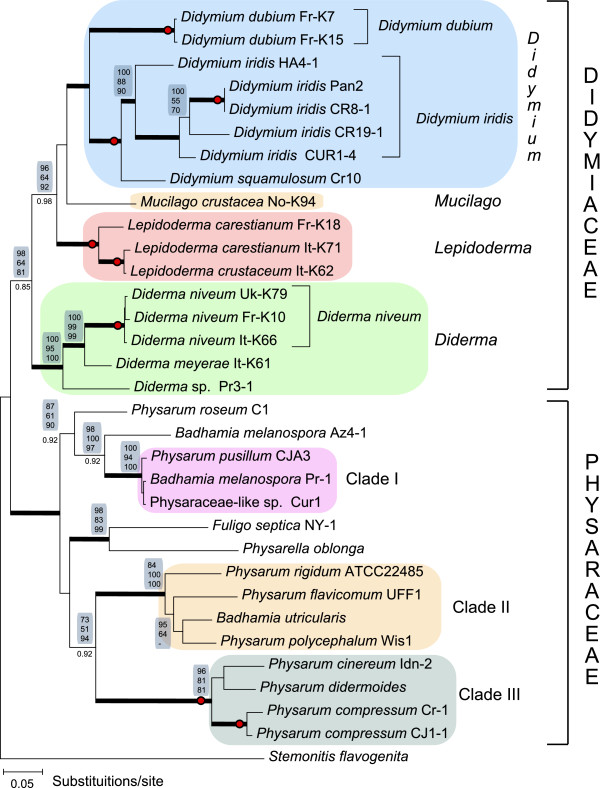
**ML phylogenetic tree of Physarales isolates based on the combined SSU/LSU data set.** The analysis was based on the alignment of 2522 nucleotide positions from 33 isolates representing the Didymiaceae and Physaraceae families as well as *Stemonitis flavogenita* (order Stemonitales) as out-group. The evolution model is GTR + I + G. Tree reconstruction using NJ, MP, ML, or BAY resulted in essential identical tree topologies. Bootstrap values (> 50%) of 2000 replications are shown above the branches (NJ/MP/ML). Node of high statistical support (bootstrap values of 100%) is indicated by red circle. BAY analysis consists of 1,000,000 generations with trees sampled every 100 generations. BAY posterior probabilities are shown below branches, and branches with posterior probabilities of 1.0 are presented as thick black lines. The Physarales clades (Clades I to III) are indicated.

**Figure 3:**
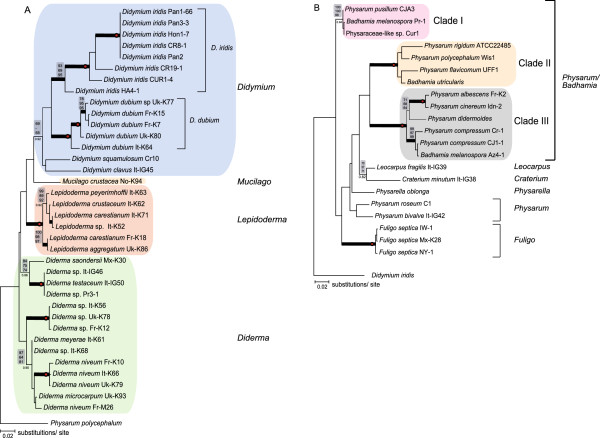
**ML phylogenetic tree of Physarales isolates based on the LSU data set.** The analysis was based on the alignment of 765 nucleotide positions from 57 isolates representing the Didymiaceae and Physaraceae families. The evolution models are TrNef + I (Didymiaceae) and GTR + I + G (Physaraceae). Tree reconstruction using NJ, MP, ML, or BAY resulted in essential similar tree topologies. Bootstrap values (> 50%) of 2000 replications are shown above the branches (NJ/MP/ML). Node of high statistical support (bootstrap values of 100%) is indicated by red circle. BAY analysis consists of 1,000,000 generations with trees sampled every 100 generations. BAY posterior probabilities are shown below branches, and branches with posterior probabilities of 1.0 are presented as thick black lines. **A**. ML-tree of Didymiaceae isolates. *P. polycephalum* was used as an out-group. **B**. ML-tree of Physaraceae isolates. *D. iridis* was used as an out-group.

A representative ML tree based on the SSU/LSU data set is presented in Figure 2. The ML tree includes 17 isolates (4 genera, 8 defined species) of the Didymiaceae family, and supported by NJ/MP/ML > 64% and BAY posterior probability > 0.85 (Table 2). The monophyly of the four genera (*Didymium*, *Lepidoderma*, *Mucilago* and *Diderma*) was supported by NJ/MP/ML > 93% and BAY posterior probability of 1.0 (Figure 2). Furthermore, phylogenetic analysis was performed on the LSU data set (Table 2) generated from 36 Didymiaceae isolates (4 genera, 17 defined species). A representative ML tree is presented in Figure 3A. This analysis is in general agreement with that of the SSU/LSU data set, but with less statistical support at basal nodes of the *Didymium* and *Diderma* genera. However, the *Lepidoderma* genus was strongly supported in both data sets (Figure 2 and Figure 3A). These findings corroborate more recent studies of Didymiaceae relationships [11,12], and we conclude that rDNA-based phylogeny is well consistent with the current Didymiaceae taxonomy [2,32].

**Table 2:** Didymiaceae isolates and rDNA sequences

**Species**	**Isolate**	**SSU**^**(a)**^	**Accession no**^**(b)**^	**LSU**^**(a)**^	**Accession no**^**(b)**^
***Didymium***					
*D. clavus*	It-IG45	na		+	HE655047
*D. dubium*	Fr-K7	+	HE614606	+	AM407422
*D. dubium*	Fr-K15	+	HE614607	+	AM407423
*D. dubium*	It-K64	na		+	HE655048
*D. dubium*	Uk-K80	na		+	HE655049
*D. dubium*	Uk-K77	na		+	HE655050
*D. iridis*	Pan1-66	na		+	AM407418
*D. iridis*	Pan2	+	AJ938153	+	AM407414
*D. iridis*	Pan3-3	na		+	AM407419
*D. iridis*	Hon1-7	+	AJ938152	+	HE655051
*D. iridis*	CUR1-4	+	AJ938150	+	AM407420
*D. iridis*	HA4-1	+	AJ938149	+	AM407416
*D. iridis*	CR19-1	+	AJ938151	+	AM407421
*D. iridis*	CR8-1	+	AJ938154	+	AM407415
*D. squamulosum*	Cr10	+	HE614613	+	AM407427
***Diderma***					
*D. meyerae*	It-K61	+	HE614614	+	HE655059
*D. microcarpum*	Uk-K93	na		+	HE655052
*D. niveum*	Fr-K10	+	HE614615	+	AM407429
*D. niveum*	Fr-M26	na	+		AM407425
*D. niveum*	It-K66	+	HE614616	+	HE655060
*D. niveum*	Uk-K79	+	HE614617	+	HE655061
*D. saondersii*	Mx-K30	na		+	AM407428
*D. testaceum*	It-IG50	na		+	HE655053
*Diderma* sp.	It-K68	na		+	HE655054
*Diderma* sp.	Fr-K12	na		+	AM407426
*Diderma* sp.	It-K56	na		+	HE655057
*Diderma* sp.	Uk-K78	na		+	HE655058
*Diderma* sp.	It-IG46	na		+	HE655055
*Diderma* sp.^(c)^	Pr3-1	+	HE614612	+	HE655056
***Lepidoderma***					
*L. aggregatum*	Uk-K86	na		+	HE655062
*L. carestianum*	Fr-K18	+	HE614609	+	AM407430
*L. carestianum*	It-K71	+	HE614618	+	HE655063
*L. crustaceum*	It-K62	+	HE614619	+	HE655064
*L. peyerimhoffii*	It-K63	na		+	HE655065
*Lepidoderma* sp.	It-K52	na		+	HE655066
***Mucilago***					
*M. crustacea*	No-K94	+	HE614620	+	HE655067

^(a)^ SSU and LSU rDNA sequence.

^(b)^ GenBank/EMBL/DDJB accession numbers.

^(c)^ Reported previous as *Didymium anellus* in [11,12].

+, analysed; na, not analysed.

### Relationships within Physaraceae

Figure 2 presents molecular phylogeny of 15 isolates (4 genera, 13 defined species) of the family Physaraceae based on the SSU/LSU data set. These isolates were separated from those of Didymiaceae with strong statistical supports represented by NJ/MP/ML bootstraps > 96% and BAY posterior probabilities of 1.0 (Figure 2). Furthermore, an extended analysis including 21 Physaraceae isolates (6 genera; 16 defined species; Table 3) based on the LSU data set is presented in Figure 3B. A different clustering pattern was seen among the Physaraceae genera compared to that of Didymiaceae. The ten investigated *Physarum* species were found to intersperse among isolates belonging to genera *Badhamia*, *Craterium*, *Fuligo*, *Leocarpus* and *Physarella* (Figures 2 and 3B). Three main clades (Clade I to III) with strong statistical support (SSU/LSU data set: NJ/MP/ML bootstraps > 84%, BAY posterior probabilities of 1; LSU data set: NJ/MP/ML bootstraps > 96%, BAY posterior probabilities > 0.94) were found to harbor both *Physarum* and *Badhamia* isolates (Figure 3B).

**Table 3:** Physaraceae isolates and rDNA sequences

**Species**	**Isolate**	**SSU**^**(a)**^	**Accession no**^**(b)**^	**LSU**^**(a)**^	**Accession no**^**(b)**^
***Badhamia***					
*B. melanospora*	Az4-1	+	HE614610	+	HE655068
*B. melanospora*	Pr-1	+	HE614596	+	HE655069
*B. utricularis*	--	+	HE614597	+	HE655070
Physaracea-like sp.	Cur1	+	HE614608	+	HE663133
***Craterium***					
*C. minutum*	It-IG38	na		+	HE655081
***Fuligo***					
*F. septica*	IW-1	na		+	HE655082
*F. septica*	Mx-K28	na		+	HE655083
*F. septica*	NY-1	+	AJ584697	+	AJ584697
***Leocarpus***					
*L. fragilis*	It-IG39	na		+	HE655071
***Physarella***					
*P. oblonga*	--	+	HE614598	+	HE655072
***Physarum***					
*P. albescens*	Fr-K2	na		+	HE655073
*P. bivalve*	It-IG42	na		+	HE655084
*P. cinereum*	Idn-2	+	HE614599	+	HE655074
*P. compressum*	CJ1-1	+	HE614600	+	HE655075
*P. compressum*	Cr-1	+	HE614601	+	HE655076
*P. didermoides*	--	+	HE614602	+	HE655077
*P. flavicomum*	UFF1	+	HE614611	+	X78959
*P. polycephalum*	Wis1	+	X13160	+	X60211
*P. pusillum*	CJA3	+	HE614603	+	HE655078
*P. rigidum*	ATCC22485	+	HE614604	+	HE655079
*P. roseum*	C1	+	HE614605	+	HE655080

^(a)^ SSU and LSU rDNA sequences.

^(b)^ GenBank/EMBL/DDJB accession numbers.

--, no isolate name given; +, analysed; na, not analysed.

Recent taxonomy updates recognize at least 80 and 20 species of *Physarum* and *Badhamia*, respectively [2,32]. Myxomycete taxonomy has traditionally been based on morphological characteristics of sporocarps and fruiting bodies. However, it has been debated for decades what characters that unambiguously distinguish *Physarum* and *Badhamia* genera species [33,34], and consequently some *Physarum* species in the current taxonomy has previously been assigned to the *Badhamia* genus and vice versa [32]. A similar relationship based on rRNA gene sequence phylogeny involving one *Badhamia* and two *Physarum* isolates was recently noted, but not further commented [15]. Our findings of a polyphyletic origin of the *Physarum* genus, with at least three phylogenetic clades interspersed with *Badhamia* isolates, strongly suggest a reevaluation of the current taxonomy to also include molecular data.

### Intron synapomorphy supports *Physarum* clades

**Figure 4:**
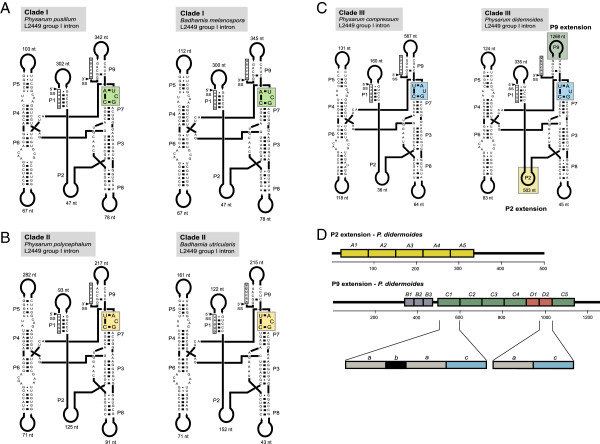
**RNA structure diagrams of L2449 group I introns in Physarales clades.** Paired elements (P1 to P9) are indicated according to the consensus of L2449 group I introns in myxomycetes [11]. The intron core and exon sequences are shown in upper case and lower case letters, respectively. Arrows indicate the 5’ splice site (SS) and the 3’ SS. Sizes of extension sequences at peripheral P-regions are indicated. The synapomorphy at the intron G-binding site in P7 is boxed. **A**. Clade I (*P. pusillum* CJA3, *B. melanospora* Pr-1) L2449 introns that contain the G-binding site motif UCG-CA (green box). **B**. Clade II (*P. polycephalum* Wis1, *B. utricularis*) L2449 introns that contain the G-binding site motif ACG-CU (yellow box). **C**. Clade III (*P. compressum* CJ1-1, *P. didermoides*) L2449 introns that contain the G-binding site motif AUG-CU (blue box). **D**. Schematic organization of large extension sequences in P2 and P9 of the 2483 nt L2449 intron in *P. didermoides* (Clade III). The 503 nt P2 extension contains five copies of an approximately 60-nt A motif. The 1268 nt P9 extension has a more complex organization of three different motifs of approximately 40 nt (B motif, 3 copies), 100 nt (C motif, 5 copies) and 55 nt (D motif, 2 copies), respectively. Here, motif C is composed by three sub-motifs (a-b-a-c), and related to motif D (a-c).

A different approach to validate the *Physarum* clades is structural analysis of the obligatory group I introns present in the LSU rRNA gene of all investigated Physarales isolates (Additional file 3: Table S2 and Additional file 4: Table S3). Both L1949 and L2449 introns have strict vertical inheritance pattern within the Physarales with potential as genetic markers [11,24]. We analyzed structural features of L2449 introns at the RNA level in more detail, and representative secondary structure diagrams of the catalytic core are presented in Figure 4A-C. Whereas the approximately 120-nt catalytic core was found conserved in sequence and structure, large extensions of various lengths were observed in all peripheral paired segment regions (P1, P2, P5, P6, P8 and P9). An extreme case was noted in the L2449 intron (2483 nt) in *P. didermoides* (Figure 4C). Here, direct repeat motifs were found both within the 503 nt P2 extension and the 1268 nt P9 extension (Figure 4D), and exemplifies the complex structural nature of myxomycete group I introns.

The most surprising structural feature, however, was found directly at the guanosine binding site (G-binding site) within the P7 segment. The G-binding site is at the catalytic center of a group I ribozyme, is highly conserved among all group I introns, and contains a universally conserved G:C pair adjacent to a bulged nucleotide (C or A). The G-binding site follows strong sequence co-variation rules, and if the bulged nucleotide is a C the first pair of the P7 segment is always an A:U or a U:A pair [35]. Figure 4A presents structures of Clade I introns (*P. pusillum* and *B. melanospora*) which have a U:A pair followed by a bulged C. Clade II introns (*P. polycephalum* and *B. utricularis*) have a slightly different G-binding site of an A:U pair followed by a bulged C (Figure 4B). A third G-binding site variant was noted in the Clade III introns (*P. compressum* and *P. didermoides*; Figure 4C). Similar to Clade II introns, the Clade III introns have a A:U as the first P7 pair, but followed by a bulged U. The latter feature is highly unusual among group I introns and to our knowledge the *Physarum* Clade III L2449 introns are the only examples among the more than 22.000 group I introns available in the sequence data base [36,37]. The fact that G-binding site variants appears to correlate with sequence-based phylogeny suggests that the intron structure character represents a synapomorphic character that further supports a complex origin of the *Physarum* genus.

## Conclusions

Partial and complete rRNA gene sequences have been obtained from a large number of myxomycete isolates and have proven to represent reliable phylogenetic markers. However, myxomycete rRNA gene sequencing is challenged by an extreme high load of group I intron elements, and we report in our data sets 222 introns interrupting coding sequences in Physarales isolates. Several of the introns have complex organisations and were thus difficult to identify at sequence level. Introns are typically located in highly conserved parts of the rRNA genes that directly interfere with the design of universal primers and may result in sampling biases in analysis approaches. We observed congruence between phylogeny and current taxonomy of the Didymiaceae isolates, but a different pattern was observed among the family Physaraceae and includes a polyphyletic origin of the genus *Physarum*. Three well supported clades containing a mixture of *Physarum* and *Badhamia* isolates were noted, suggesting reevaluation of the current taxonomy into new genera. These observations were further supported by an unusual synapomorphic character at the G-binding site of the L2449 group I introns.

## Methods

### Myxomycetes isolates and culturing

All new isolates reported were collected as sporocarps. Their geographical origin, mode of classification, name of the collector, and if a cell line was obtained, are listed in Additional file 8: Table S4. Cultivation of amoebae was essentially performed as previously described [12,38].

### DNA extraction and sequencing

Total DNA was extracted from growing amoebae as previously described [11] or directly from sporocarps without culturing (summarized in Additional file 8: Table S4). Here, DNA extraction was performed using Berlin Technologies instrument (PreCelly’s 24 tube # VK05). Two to five sporocarp heads were homogenized in 250 μl lysis buffer (4 M Guanidine thiocyanate, 50 mM Tris–HCl (pH 7.5), 10 mM EDTA, 2% SDS, 1% β-mercaptoethanol) at 5000 rpm in 30 seconds for 1 to 4 cycles. The lysed spores were treated twice with phenol-chloroform, and DNA was extracted by ethanol precipitation and re-suspended in TE buffer. The SSU and LSU rRNA gene segments were PCR-amplified from total DNA extracts in several overlapping fragments using multiple sets of specific oligonucleotides (contact SDJ for details). DNA sequences were performed on both strands using automatic sequencing (Big Dye terminator chemistry, Applied Biosystems, Foster City, CA, USA).

### Phylogenetic analyses

Three data sets based on nuclear rRNA sequences were used in the molecular phylogeny. The SSU dataset (1028 nucleotide positions), the combined SSU/LSU data set (2522 nucleotide positions), and the LSU data set (765 nucleotide positions) were manually aligned in BioEdit v.7.0.5.3 [39] based on the secondary structure models of *P. polycephalum* and *D. iridis*[22,40]. Phylogenetic trees were built with the methods of NJ applying the Jukes-Cantor nucleotide substitution model based on the number of nucleotide substitution per site [41], and MP using heuristic search with close-neighbor-interchange (CNI) level 3 and generation of 10 random initial trees, in MEGA version 4.0.2 [42]., as well as ML using PhyML interface [43] and nucleotide substitution models selected by jModelTest 0.1 package [44]. The following substitution models were used in ML: TPM2uf + I + G (SSU data set), GTR + I + G (SSU/LSU dataset), TNef + I (LSU dataset; Didymiaceae) and GTR + I + G (LSU dataset; Physaraceae). The reliabilities of tree branching points for NJ, MP and ML trees were evaluated by bootstrap analyses (2000 replications). BAY analyses were performed to reconstruct phylogenetic trees from all datasets using MrBayes, version 3.1.2 [45,46] with two independent runs and the Metropolis-coupled Markov chain Monte Carlo (MCMCMC) method. Here the evolutionary model GTR + I + G was used for all datasets. A total of 1,000,000 generations were run with sampling every 100 generations. For each dataset the standard deviation of split frequencies was below 0.015 at the end of the run. Twenty-five % of initial trees were discarded and a consensus tree with posterior probabilities was generated from the remaining 15,000 trees.

## Competing interests

The authors declare that they have no competing interests.

## Authors’ contributions

SCRN participated in rDNA sequencing and performed the phylogenetic analysis. KH collected natural isolates, performed culturing and DNA extraction, and participated in rDNA sequencing. DHC participated in the initial design of this study and in the phylogenetic analysis. EFH participated in the initial design of this study and in discussions of results. SDJ directed the research, performed intron analysis, and wrote the manuscript in collaboration with all authors. All authors read and approved the final manuscript.

## Supplementary Material

Additional file 1 **Figure S1.** ML phylogenetic tree of Mycetozoa isolates and associated amoebozoa based on the SSU data set. The analysis was based on the alignment of 1028 nucleotide positions from 26 isolates (Table 1). The evolution model is TPM2uf + I + G. Tree reconstructions using NJ, MP, ML, or BAY resulted in essential identical tree topologies. Bootstrap values (> 50%) of 2000 replications are shown at the internal nodes (NJ/MP/ML). Node of high statistical support (bootstrap values of 100%) is indicated by red circle. BAY analysis consists of 1,000,000 generations with trees sampled every 100 generations. Branches with BAY posterior probabilities of 1.0 are represented as thick black lines. Click here for file

Additional file 2 **Table S1.** Key features of group I introns distribution in selected mycetozoan and associated amoebozoan isolates. Click here for file

Additional file 3 **Table S2.** Key features of group I introns distribution in Didymiaceae isolates. Click here for file

Additional file 4 **Table S3.** Key features of group I introns distribution in Physaraceae isolates. Click here for file

Additional file 5 **Figure S2.** Alignment of the Physarales SSU/LSU data set. Click here for file

Additional file 6 **Figure S3.** Alignment of the Didymiaceae LSU data set. Click here for file

Additional file 7 **Figure S4.** Alignment of the Physaraceae LSU data set. Click here for file

Additional file 8 **Table S4.** Geographic origin, classification and culturing of the myxomycete isolates. Click here for file
