# Supplementary material for: Polyphyletic origin of the genus Physarum (Physarales, Myxomycetes) revealed by nuclear rDNA mini-chromosome analysis and group I intron synapomorphy
Source: BMC Evol Biol. 2012 Aug 31;12:166. doi: 10.1186/1471-2148-12-166 (PMC3511172; doi:10.1186/1471-2148-12-166)
Supplement: Additional file 2 — Table S1. Key features of group I introns distribution in selected mycetozoan and associated amoebozoan isolates. [file 1471-2148-12-166-S2.pdf]

TABLE S1. Group I intron distribution: Mycetozoan and representative amoebozoan isolates

Species	Isolate [a]	SSU-intron [b]	LSU-intron [b]	Acc No [c]
MYCETOZOA				
**Myxomycetes** (plasmodial slime molds)				
**Stemonitales (Order)**				
Comatricha nigricapillitia	AMFD114	S516	na	AY643824
Stemonites flavogenita	ATCC24714	NO	L1949	HE614592; HE655085
**Trichiales (Order)**				
Arcyria stipata	AMFD257	S529; S1199	na	EF513170
Trichia persimilis	--	NO	na	AY643826
**Liceales (Order)**				
Cribraria cancellata	AMFD94	S956	na	EF513177
**Echinosteliales (Order)**				
Echinostelium minutum	ATCC24714	NO	NO	HE614593; HE655087
**Protostelids**				
Soliformovum irregulare	ATCC26826	NO	NO	HE614594; HE655088
**Dictyostelids** (cellular slime molds)				
Acytostelium ellipticum	ATCC22247	NO	L1949	HE614595; HE655086
Acytostelium leptosomum	FG12	NO	na	AM168111
Acytostelium subglobosum	LB1	NO	na	AM168110
Dictyostelium discoideum	--	NO	na	K02641
Dictyostelium fasciculatum	SH3	NO	na	AM168087
Dictyostelium medusoides	OH592	NO	na	AM168088
Dictyostelium rhizopodium	AusKY-4	NO	na	AM168063
**Acrasids**				
Acrasis rosea	T-235	S529; S943; S956	na	AF011458
AMOEBOZOA				
Amoeba leningradensis	CCAP1503/6	NO	na	AJ314605
Acanthamoeba palestinensis	CCAP1547/1	NO	na	L09599
Entamoeba histolytica	HM1-IMSS	NO	NO	X65163
Filamoeba nolandi	ATCC50430	NO	na	AF293896
Gephyramoeba sp.	ATCC50654	NO	na	AF293897
Hartmannella abertawensis	--	NO	na	DQ190241
Mastigella commutans	--	NO	na	AF4212129
Naegleria gruberi	NEG-M	NO	NO	AB298288
Platyamoeba placida	--	NO	na	AY294150

[a] Source of the organism. [b] Intron insertion site (*E.coli* numbering) in the small subunit and large subunit rRNA. [c] GenBank/EMBL/DDJB accession numbers.
--, no isolate name given; NO, no presence of introns; na, not analysed.
