# Supplementary material for: Polyphyletic origin of the genus Physarum (Physarales, Myxomycetes) revealed by nuclear rDNA mini-chromosome analysis and group I intron synapomorphy
Source: BMC Evol Biol. 2012 Aug 31;12:166. doi: 10.1186/1471-2148-12-166 (PMC3511172; doi:10.1186/1471-2148-12-166)
Supplement: Additional file 3 — Table S2. Key features of group I introns distribution in Didymiaceae isolates. [file 1471-2148-12-166-S3.pdf]

TABLE S2. Group I intron distribution: Didymiaceae isolates

Species	Isolate	SSU-intron [(a)]	LSU-intron [(a)]	Acc No
Didymium				
D. clavus	It-IG45	na	L1949; L2449	HE655047
D. dubium	Fr-K7	NO	L1949; L2449	HE614606; AM407422
D. dubium	Fr-K15	NO	L1949; L2449	HE614607; AM407423
D. dubium	It-K64	na	L1949; L2449	HE655048
D. dubium	Uk-K80	na	L1949; L2449	HE655049
D. dubium	Uk-K77	na	L1949; L2449	HE655050
D. iridis	Pan1-66	na	L1949; L2449	AM407418
D. iridis	Pan2	S956	L1949; L2449	AJ938153; AM407414
D. iridis	Pan3-3	na	L1949; L2449	AM407419
D. iridis	Hon1-7	NO	L1949; L2449	AJ938152; HE655051
D. iridis	CUR1-4	S1389	L1949; L2449	AJ938150; AM407420
D. iridis	HA4-1	NO	L1949; L2449	AJ938149; AM407416
D. iridis	CR19-1	S1389	L1949; L2449	AJ938151; AM407421
D. iridis	CR8-1	S956	L1949; L2449	AJ938154; AM407415
D. squamulosum	Cr10	S529; S1389	L1949; L2066; L2449	HE614613; AM407427
Diderma				
D. meyerae	It-K61	S529; S788; S956; S1389	L1926; L1949; L2066; L2449	HE614614; HE655059
D. microcarpum	Uk-K93	na	L1949; L2449	HE655052
D. niveum	Fr-K10	S516; S529; S788; S911; S943; S956; S1065; S1199; S1389	L1911; L1926; L1949; L1975; L2066; L2449; L2499	HE614615; AM407429
D. niveum	Fr-M26	na	L1949; L2449	AM407425
D. niveum	It-K66	S516; S529; S788; S911; S943; S956; S1065; S1199; S1389	L1911; L1926; L1949; L1975; L2066; L2449; L2499	HE614616; HE655060
D. niveum	Uk-K79	S516; S529; S788; S911; S943; S956; S1065; S1199; S1389	L1911; L1926; L1949; L1975; L2066; L2449; L2499	HE614617; HE655061
D. saondersii	Mx-K30	na	L1949; L2066; L2449	AM407428
D. testaceum	It-IG50	na	L1949; L2449	HE655053
Diderma sp.	It-K68	na	L1949; L2449	HE655054
Diderma sp.	Fr-K12	na	L1949; L2449	AM407426
Diderma sp.	It-K56	na	L1949; L2066; L2449	HE655057
Diderma sp.	Uk-K78	na	L1949; L2066; L2449	HE655058
Diderma sp.	It-IG46	na	L1949; L2449	HE655055
Diderma sp.[(b)]	Pr3-1	S529; S1389	L1949; L2449	HE614612; HE655056
Lepidoderma				
L. aggregatum	Uk-K86	na	L1949; L2066; L2449	HE655062
L. carestianum	Fr-K18	NO	L1926; L1949; L2066; L2449	HE614609; AM407430
L. carestianum	It-K71	S529; S788; S911; S956; S1199; S1389	L1921; L1949; L1975; L2449; L2499	HE614618; HE655063
L. crustaceum	It-K62	S516; S529; S788; S911; S956; S1065; S1199; S1389	L1911; L1921; L1926; L1949; L1975; L2066; L2449	HE614619; HE655064
L. peyerimhoffii	It-K63	na	L1921; L1949; L1975; L2066; L2449; L2499	HE655065
Lepidoderma sp.	It-K52	na	L1921; L1949; L1975; L2449	HE655066
Mucilago				
M. crustacea	No-K94	S788; S1389	L1926; L1949; L2066; L2449	HE614620; HE655047

[(a)] Introns insertion site (*E.coli* numbering) in small subunit (SSU) and large subunit (LSU) rRNAs according to [47]. [(c)] Reported previous as *Didymium anellus* in [11,12]. NO, no presence of introns; na, not analysed.
