# Supplementary material for: Polyphyletic origin of the genus Physarum (Physarales, Myxomycetes) revealed by nuclear rDNA mini-chromosome analysis and group I intron synapomorphy
Source: BMC Evol Biol. 2012 Aug 31;12:166. doi: 10.1186/1471-2148-12-166 (PMC3511172; doi:10.1186/1471-2148-12-166)
Supplement: Additional file 4 — Table S3. Key features of group I introns distribution in Physaraceae isolates. [file 1471-2148-12-166-S4.pdf]

TABLE S3. Group I intron distribution: Physaraceae isolates

Species	Isolate	SSU-intron [a]	LSU-intron [a]	Accession no [b]
Badhamia				
B. melanospora	Az4-1	S516	L1949; L2449	HE614610; HE655068
B. melanospora	Pr-1	NO	L1949; L2449	HE614596; HE655069
B. utricularis	--	NO	L1949; L2449	HE614597; HE655070
Physaracea-like sp.	Cur1	NO	L1949; L2449	HE614608; HE663133
Craterium				
C. minutum	It-IG38	na	L1949; L2066; L2449	HE655081
Fuligo				
F. septica	IW-1	na	L1949; L2066; L2449; L2499	HE655082
F. septica	Mx-K28	na	L1925; L1949; L2449	HE655083
F. septica	NY-1	S516; S911; S956; S1065	L1911; L1949; L2449; L2499	AJ584497
Leocarpus				
L. fragilis	It-IG39	na	L1949; L2449	HE655071
Physarella				
P. oblonga	--	NO	L1949; L2449	HE614598; HE655072
Physarum				
P. albescens	Fr-K2	na	L1949; L2449	HE655073
P. bivalve	It-IG42	na	L1925; L1949; L2449	HE655084
P. cinereum	Idn-2	NO	L1949; L2449	HE614599; HE655074
P. compressum	CJ1-1	NO	L1949; L2449	HE614600; HE655075
P. compressum	Cr-1	NO	L1949; L2449	HE614601; HE655076
P. didermoides	--	NO	L1949; L2449	HE614602; HE655077
P. flavicomum	UFF1	S516	L1949; L2449	HE614611; X78959
P. polycephalum	Wis1	NO	L1949; L2449	X13160; X60211
P. pusillum	CJA3	NO	L1949; L2449	HE614603; HE655078
P. rigidum	ATCC22485	NO	L1949; L2449	HE614604; HE655079
P. roseum	C1	NO	L1949; L2449	HE614605; HE655080

[a] Intron insertion site (*E.coli* numbering) in the small subunit (SSU) and large subunit (LSU) rRNA. [b] GenBank/EMBL/DDJB accession numbers. NO, no presence of introns; na, not analysed.
