# Supplementary material for: Polyphyletic origin of the genus Physarum (Physarales, Myxomycetes) revealed by nuclear rDNA mini-chromosome analysis and group I intron synapomorphy
Source: BMC Evol Biol. 2012 Aug 31;12:166. doi: 10.1186/1471-2148-12-166 (PMC3511172; doi:10.1186/1471-2148-12-166)
Supplement: Additional file 5 — Figure S2. Alignment of the Physarales SSU/LSU data set. [file 1471-2148-12-166-S5.pdf]

Additional file Figure S2

SSU/LSU alignment (2522 nt)

Variable sites: 980
Parsimony informative characters: 775

```
#NEXUS
BEGIN DATA;
dimensions ntax=33 nchar=2522;
interleave=yes datatype=DNA gap=-;

matrix
CUR1_4             AGCAATTCTCTCTGAATCTGCGAACGGCTCCGCACACCAGTTGTAAACTA
HA4_1              AGCAATTCTCTCTGAATCTGCGAACGGCTCCGCAAACCAGTTGTAAACTA
CR19_1             AGCAATTCTCTCTGAATCTGCGAACGGCTCCGCACACCAGTTGTAAACTA
Pan2               AGCAATTCTCTCTGAATCTGCGAACGGCTCCGCAAACCAGTTGTAAACTA
CR8_1              AGCAATTCTCTCTGAATCTGCGAACGGCTCCGCAAACCAGTTGTAAACTA
Fr_K7              AGCAATTCTCTCTGAATCTGCGAACGGCTCCGCAAACCAGTTGTAAACTA
Fr_K15             AGCAATTCTCTCTGAATCTGCGAACGGCTCCGCAAACCAGTTGTAAACTA
CR10               AGCAATTCTCTCTGAATCTGCGAACGGCTCCGCAAACCAGTTGTAAACTA
It_K61             AGCAATTCTCTCTGAATCTGCGAACGGCTCCGCAAACCAGTTGTAAACCA
Fr_K10             AGCAATTCTCTCTGAATCTGCGAACGGCTCCGCACACCAGTTGTAAACCA
It_K66             AGCAATTCTCTCTGAATCTGCGAACGGCTCCGCACACCAGTTGTAAACCA
Uk_K79             AGCAATTCTCTCTGAATCTGCGAACGGCTCCGCACACCAGTTGTAAACCA
Pr3_1              AGCAATTCTCTCTGAATCTGCGAACGGCTCCGCACACCAGTTGTAAACTA
It_K71             AGCAATTCTCTCTGAATCTGCGAACGGCTCCGCAAACCAGTTGTAAACTA
Fr_K18             AGCAATTCTCTCTGAATCTGCGAACGGCTCCGCAAACCAGTTGTAAACTA
Fr_K62             AGCAATTCTCTCTGAATCTGCGAACGGCTCCGCAAACCAGTTGTAAACTA
No_K94             AGCAATTCTCTCTGAATCTGCGAACGGCTCCGCAAACCAGTTGTAAACAA
Cur_1              AGCAACTCTCTCTGAATCTGCGAACGGCTCCGCAAACCAGTTGTAAACCA
Az4_1              AGCACATCTCTCTGAATCTGCGAACGGCTCCGCAAACCAGTTGTAAACCA
PR_1               AGCAACTCTCTCTGAATCTGCGAACGGCTCCGCAAACCAGTTGTAAACCA
Butricularis       AGCAAGTCTCTCTGAATCTGCGAACGGCTCCGCATACCAGTTGTAAACCA
NY_1               AGCAAGTCTCTCTGAATCTGCGTACGGCTCCGCAAACCAGTTGTAAACCA
Poblonga           AGCAAATCTCTCCGAATCTGCGGACGGCTCCGCACACCAGTTGTAAACAA
Idn2               AGCAA-TCTCTTTGAATCTGCGAACGGCTCCGCACACCAGTTGTAAACTA
CJ1_1              AGCAT-TCTCTCTGAATCTGCGAACGGCTCCGCACACCAGTTGTAAACTA
CR_1               AGCAT-TCTCTCTGAATCTGCGAACGGCTCCGCACACCAGTTGTAAACTA
Pdidermoides       AGCTT-TCTCTCTGAATCTGCGAACGGCTCCGCAAACCAGTTGTAAACTA
UFF1               AGCAAGTCTCTCTGAATCTGCGAACGGCTCCGCATACCAGTTGTAAACCA
CJA3               AGCAACTCTCTCTGAATCTGCGAACGGCTCCGCAAACCAGTTGTAAACCA
Prigidum           AGCAAGTCTCTCTGAATCTGCGAACGGCTCCGCATACCAGTTGTAAACCA
C1                 AGCAATTCTCTCCGAATCTGCGAACGGCTCCGCAAACCAGTTGTAAACCA
PpolycephalumWis1  AGCAAGTCTCTCTGAATCTGCGAACGGCTCCGCATACCAGTTGTAAACCA
Sflavogenita       AGCAATTCTCTCTGAATCTGCGAACGGCTCCGCAAACCAGTTGTAAACCA
```

```
CUR1_4             TAGCAAGCATGCCCATATCCCTTCGGGGGTGTGGGTCACATGGATAACCC
HA4_1              TAGCAAGCAAGCCCTAGGCCCCTCGGGGTCTGGGGCAACATGGATAACCC
CR19_1             TAGCAAGCATGCCCATATCCCTTCGGGGGTGTGGGTCACATGGATAACCC
Pan2               TAGCAAGCAAGCCCGTGCCCTCTCGGGGGTGCGGGCAACACGGATAACCC
CR8_1              TAG-AAGCAAGCCCGTGCCCTCTCGGGGGTGCGGGCAACACGGATAACCC
Fr_K7              TAGCAAGCTCTCCTATGATCGCCCCTTCACGGGGGGGATAGGGATAACCC
Fr_K15             TAGCAAGCTCTCCTATGGTCGCCCCTTCACGGGGGGGATAGGGATAACCC
CR10               TAGCAAGCCCGCCCGGGGTCCCTTCACGGGGGCTCTGACACGGATAACCC
It_K61             AAGCAAGCATACTCGGGGTTTGCCCTTCACGGGGTCGATACGGGTAACCC
Fr_K10             AAGCAAGCTAGTCCATGGCCTGGCCCTCACGGGTTGGATACGGATAACCC
It_K66             AAGCAAGCTAGTCCATGGTCTGGCCCTCACGGGTTGGATACGGATAACCC
Uk_K79             AAGCAAGCTAGTCCATGGTCTGGCCCTCACGGGTTGGATACGGATAACCC
Pr3_1              TAGCAAGCATGCTCGGTGTCCGGCCTTCACGGGCTGGATAAGGATAACCC
It_K71             TAGCAAGCAATCCTGTGGTTGGGCTTCGGCTCGGCCAACATGGATAACCC
Fr_K18             TAGCAAGCAATCCTGCGGGCGGCTTCGGCTGCTCGTGACATGGATAACCC
Fr_K62             TAGCAAGCAATCCTGTGGTTGGGCTTCGGCTCGGCCAACATGGATAACCC
No_K94             TAGCAAGCAAGCCCGGGTCTGGTCTTCACGGGTCAGGACACGGATAACCC
Cur_1              TAGCAAGCACGCCTTGGGCCGCCGCAAGGCGGTCCTTATAGGGATAACCC
Az4_1              TAGCAAGCATGCCTTGGGACCCGGTCGCAAGGCCGGGACAGGGATAACCC
PR_1               TAGCAAGCACGCCTTGGGCCGCCGCAAGGCGGTCCTTATAGGGATAACCC
Butricularis       TAGCAAGCAAACCGGGCGGTCGTTTCACGACGCATCGATAAGGATACCCA
NY_1               TAGCTAGCAGGCCTCGGAGACGACCGCAAGTCGTAGCAAAAGGATATCCA
Poblonga           TGGCAAGCCGGTTTTCGAGATCGCCGCAAGGTGTTTCACAAGGCTATCCC
Idn2               TAGCAAGTCCGTACGGCTAGCGAGCAATCGTTAGTCGACAGGGATAACCC
CJ1_1              TAGCAAGTCCGCATGATCATGGGCAACCGTGGTTGTGACAGGGATAACCC
CR_1               TAGCAAGTCCGCATGACCATGGGCAACCGTGGTTGTGACAGGGATAACCC
Pdidermoides       TAGCAAGTCCGTACGGTTAGGGAGCAATCCTTAGCTGACAGGGATAACCC
UFF1               TAGCAAGCAAGCTCGATCGTCTTCCCCTCGTCCTCGTACAGGGATATCCC
CJA3               TAGCAAGCACGCCTTGGGCCGCCGCAAGGCGGTCCTTATAGGGATAACCC
Prigidum           TAGCGAGCAAGCCGCGTCGGCGTCGCAAGGCGTTCGGACAAGGATAACCC
C1                 TAGCGAGCATGCCCTTGGTCCGGCCGCAAGGCTGGGCATAAGGATAACCC
PpolycephalumWis1  TAGCAAGCAAGCCGCGTTGTTGCCGCAAGGCGACGGCACAGGGATAACCC
Sflavogenita       TAGC-AGCGTTCCGCCCGGCTTCAGCAATGGGGCCGGATATGGATAACCG

CUR1_4             TGGTAATTCTGAGGCTAATACGAGAACGTACAGCCTGGT-CGACTGGGAA
HA4_1              TGGTAATTCTGAGGCTAATACAAGAACGTAATACCTGGC-TAACCGGTTC
CR19_1             TGGTAATTCTGAGGCTAATACGAGAACGTACAGCCTGGT-CGACTGGGAA
Pan2               TGGTAATTCTGAGGCTAATACGAGAACGTACTCCCCGGT-CAACCGGGCA
CR8_1              TGGTAATTCTGAGGCTAATACGAGAACGTACTCCCCGGT-CAACCGGGCA
Fr_K7              TGGTAATTCTGAGGCTAATACCACCACGTAAAACCTGGC-TGACTGGGCA
Fr_K15             TGGTAATTCTGAGGCTAATACCACCACGTAAAACCTGGC-TGACTGGGCA
CR10               TGGTAATTCTGAGGCTAATACGAGAACGTAATACCTGGC-TGACCGGGCA
It_K61             TGGTAATTCTGAGGCTAATACGAGAATGTACTCCCTGGC-TAACCGGGAA
Fr_K10             TGGTAATTCTGAGGCTAATACGAGACTGCAATCCCTGGC-TAACCGCAA-
It_K66             TGGTAATTCTGAGGCTAATACGAGACTGCAATCCCTGGC-TAACCGCAA-
Uk_K79             TGGTAATTCTGAGGCTAATACGAGACTGCAATCCCTGGC-TAACCGCAA-
Pr3_1              TGGTAATTCTGAGGCTAATACAAGACCGTAATCCCTCGC-TGACTGGGTA
It_K71             TGGTAATTCTGAGGCTAATACAAGAACGTACAACCTGGC-TAACCGG-CA
Fr_K18             TGGTAATTCTGAGGCTAATACAAGAACGTACTCCCTGGC-TGACCGG-CA
Fr_K62             TGGTAATTCTGAGGCTAATACAAGAACGTACAACCTGGC-TAACCGG-CA
No_K94             TGGTAATTCTGAGGCTAATACGAGAACGCAACCCCTGGT-CGACCGGGCA
Cur_1              TGGTAATTCTGAGGCTAATACATGAACGTAACCCCCGGC-CGAC-GGGCA
Az4_1              TGGTAATTCTGAGGCTAATACATGAACGTACCCCCTGGC-CGAC-GGGCA
PR_1               TGGTAATTCTGAGGCTAATACATGAACGTAACCCCCGGC-CGAC-AGGCA
Butricularis       TGGTAATTCTGAGGCTAATACAAGAACGTACAACCCGCT-CGACC-CGTT
NY_1               TGGTAATTCTGAGGCTAATACTTGACTGTAACCCCTGGC-TGACCGGGCA
Poblonga           TGGCAATTCTGAGGCTAATACAAGAACATACCACCTGGC-TGACCGGGCA
Idn2               TGGTAATCCTGAGGCTAATACAAGAACGTAAAACTCGGT-CGACTGGGCA
CJ1_1              TGGTAATTCTGAGGCTAATACAAGAACGTAAAACTCGGT-TAACCGGTTC
CR_1               TGGTAATTCTGAGGCTAATACAAGAACGTAAAACTCGGT-TGACTGGTTT
Pdidermoides       TGGTAATCCTGAGGCTAATACAAGAACGTAAAACTCGGT-CGACTGGGCA
UFF1               TGGTAATTCTGAGGCTAATACAAGAATATACGGCCCGGCCCGACCTCGCA
CJA3               TGGTAATTCTGAGGCTAATACATGAACGTAACCCCCGGC-CGAC-GGGCA
Prigidum           TGGTAATCCTGAGGCTAATACAAGAACGTAGCACTCGTTTCGACC-CGCA
C1                 TGGTAATTCTGAGGCTAATACATGAACGCAAAACCTGGC-TGACCGGGTA
PpolycephalumWis1  TGGTAATTCTGAGGCTAATACAAGAACGTACCACCCGCTTCGACC-CGTA
Sflavogenita       TGGTAATTCTGAGGCTAATACATGAACACTAACCCGGGC-TGGGGGCAAC
```

```
CUR1_4             ACTGGAAGGCTGGGAGGTGTGTATTGTG-CACGAGGAATCTGCTGGGATA
HA4_1              GCTGGAAGGCTGGGAGACGTGTGATGCTTCGCGAGGAATTTGCTGGGATA
CR19_1             ACTGGAAGGCTGGGAGGTGTGTATTGTG-CACGAGGAATCTGCTGGGATA
Pan2               ACTGGAAGGCTGGGAGGTGTGTATTGAG-CACGAGGAATCTGCTGGGATA
CR8_1              ACTGGAAGGCTGGGAGGTGTGTATTGAG-CACGAGGAATCTGCTGGGATA
Fr_K7              ACCGGAAGGCTGGGGGGCGTGCATTTTTTCGCGAG--GTTTACCGGGAGG
Fr_K15             ACTGGAAGGCTGGGGGGCGTGCATTTTTTCGCGAG--GTTTACCGGGAGG
CR10               ACTGGAAGGCTGGGAGACGTGCGTTGTGTCGCGAG-AATCTATCGGGATG
It_K61             ACTGGTAGGCTGGGGGGCGTGTATCTATTTGCAAGACG-ATTCCAGGAGG
Fr_K10             ---GGAAGGCTGGGGGGCGTGTAAGGTTTCGCGAGGAA-TAACCGGGAGG
It_K66             ---GGAAGGCTGGGGGGCGTGTAAGGTTTCGCGAGGAA-TAACCGGGAGG
Uk_K79             ---GGAAGGCTGGGGGGCGTGTAAGGTTTCGCGAGGAA-TAACCGGGAGG
Pr3_1              ACTGGGAGGCTGGGGGACGTGTGTTTGGTCGCAAGG-A-TGACCGGGAAG
It_K71             AC-GGAAGGCTGGGGAACGTGCAACGTATCGTAGTGG--ATACCGGGAAG
Fr_K18             AC-GGAAGGCTGGGGAGCGTGCAACGTATCGTAGGGAT-AAACCGGGAAT
Fr_K62             AC-GGAAGGCTGGGGAACGTGCAACGTATCGTAATGG--ATACCGGGAAG
No_K94             ACTGGAAGGTTGGGAAGCGTGTACTAGATCGCGAGGAAGACGCCGGGAAG
Cur_1              ACTGGAAG-CCGGGGACCGTGCGTTAAG---CGTAACAGTTTATCGGAGT
Az4_1              ACCGGAGG-CTGGGGACCGTGCGT-AGG---CGCAA-AGTTATTGGGAGT
PR_1               ACTGGAAG-CCGGGGACCGTGCGTTAAG---CGTAACGATTTATCGGAGT
Butricularis       AGGGGA-GGGGGGGGAATGTGTCTAAGGCA-CGCAAATATCGTACTGAGT
NY_1               ACTGGTAGGCTGGGGGACGTGTGGCTTC---GCACACCATTA--AGGAAT
Poblonga           ACGGGGACGCTGGGTGCGGTGTGACCCT---CGCTAAGGTTGCTATGAGA
Idn2               ACCGGAAGGCCGAGGGTCGTGT-GCGCT-A-TGCAATACTCGACATGATT
CJ1_1              GCTGGAAGGCCGAGGGTCGTGTGGCTTTTACCGCAACAC-CGACACGAAA
CR_1               ACTGGAAGGCCGAGGGTCGTGTAGTTTTTACTGCAACAC-CGACACGAAA
Pdidermoides       ACCGGAAGGCTGAGGGTCGTGTCGCTTT-ACTGCAACGCTCGACATGAAT
UFF1               AGGGGAAGGGTGGGGGACGTGTCC-AGGCA-CGCAAAATAGCACCTGAGA
CJA3               ACTGTAAG-CCGGGGACCGTGCGTTAAG---CGTAACGATTTATCGGAGT
Prigidum           AGGGGA-GAACGGGGACTGTGTCC-AGGCA-CGCAAT--TTGATCTGAGT
C1                 ACTGGAAGGCTGGGGGACGTGTCTGAGT---CGCAA-TTTTGCTGGGAGT
PpolycephalumWis1  AGGGGA-GGGCGGGGGTTGTGTCCCAGGT--CGCAAATATTA-ACTGAGT
Sflavogenita       CTCGGCTTGGTGGGGGACGTGCACCGGCTACAATTAAAAATACCGCGAAA

CUR1_4             GACA-CACGCCTGGACCGCGCTAAAAACAGTCAGGGCTTCCTTTCGGGCG
HA4_1              GATG-CACGTCTGGACCGCGCTAAACACGGATAGGCCCTCTGAACCTGCG
CR19_1             GACA-CACGCCTGGACCGCGCTAAAAACAGTCAGGGCTTCCTTTCGGGCG
Pan2               GACA-CACGCCTGGACCGCGCCAAAAACGGATGGGGCTTCCTTCACGGCG
CR8_1              GACA-CACGCCTGGACCGCGCCAAAAACGGATGGGGCTTCCTTCACGGCG
Fr_K7              GACG-CACGTCTGGACCGCGCA--AAACGGATGACTGGGCCTTCGGGGCG
Fr_K15             GACG-CACGTCTGGACCGCGCA--AAACGGATGACTGGGCCTTCGGGGCG
CR10               AACG-CACGTCTGGACCGCGCTAAACACGGATGGGTGCCCTTGCCTTGCG
It_K61             GGTG-CACGCTCTGACCGCGACAAAGACAGTTCTGACGGACCCTCACGCG
Fr_K10             GGTA-CACGCTCTGACCACGACAAAGACAGTCCCGGTAAATCTTCTGGTG
It_K66             GGTA-CACGCTCTGACCACGACAAAGACAGTTCCGGCAAATCCTCGGGTG
Uk_K79             GGTA-CACGCTCTGACCACGACAAAGACAGTTCCGGCAAATCTTCGGGTG
Pr3_1              GATG-CACGTTCTGACCGCGATAAAAACGGATCACTTNGGTGGCCCGGCG
It_K71             GGTG-CACGTTTGGACCGCGCTAAAGACAGTCCTTGGTAAGCCCTTGGCG
Fr_K18             GGGTGCACGCTTGGACCGCGCTAAAGACAGATCTCGGCGAGCCTTCGGCG
Fr_K62             GGTG-CACGTTTGGACCGCGCTAAAAACAGTCCTTGGTAAGCCCTCAGCG
No_K94             GATG-CACGCTTGGACCGCGCTAAACAAGGATCAGGTTACCGACCTCGCG
Cur_1              AGTCGCACAGTACGACCACCGTAAAAACGGTATCCGCTTCGTCGCCTGTG
Az4_1              AGTCGCACAGTAAGACCATCATAAAAACGGTCCTCGTTTCGCTGGCCGTG
PR_1               AGTCGCACAGTACGACCACCATAAAAACGGTATCCGCTTCGTCGCCTGTG
Butricularis       GGCCACACGTTCAGACCACCATACAAACGGTCGTTCGCTTCCGAAAGGTG
NY_1               GAATGCACGTTCAGACCGCTTTTCACACAGATAGGGGGTAATCCTTCGCG
Poblonga           AGCTGCACGCTCTGACCCACTGCAAAACGGGCTCGCTTGTGAACNCTGTG
Idn2               GGATGCACGATCAAACCACCATACAAACGATATCGTCGCGTAAGCGCGTG
CJ1_1              GAGTGCACGATCAAACCACCATAAAAACGATAGGGCAGGGAAACCCCGTG
CR_1               GAGTGCACGATCAAACCACCATAAAAACGATAGGGCAGGGAAACCCCGTG
Pdidermoides       GAGTGCACGATCAAACCACCATACAAACGGATTAGTCGGGAAGCCTTGTG
UFF1               GGCCACACGTNCGGACCGCCACACAAACGGTTGCTCTCGTCTCCGACGCG
CJA3               AGTCGCACAGTACGACCACCATAAAAACGGTATCCGCTTCGCTGCCTGTG
Prigidum           GGCCACACGATCAGACCGCTATTCAAACAGTCGCTTGCTCGAACGCCGCG
C1                 AGACGCACGTTCAGACCACCATACAAACAGTCTTGCCGGGGTTCGCCGTG
PpolycephalumWis1  GGCCACACGATCTGACCACCATACAAACGGTTATCCGCTTCGAAAGCGTG
Sflavogenita       GGACGCACGTTCAGACCGCCATCCAAACGGGGGAGGGGGCTTCGGCTGCG
```

```
CUR1_4              GCTCTTGCTTTGTGCTTCTGACCTATCAACTAGATGGCAGCGTAACGGAC
HA4_1               GCTCTTGCTTTGTGCTTCTGACCTATCAACTAGACGGCAGCGTAATGGAC
CR19_1              GCTCTTGCTTTGTGCTTCTGACCTATCAACTAGATGGCAGCGTAACGGAC
Pan2                GCTCTTGCTTTGTGCTTCTGACCTATCAACTAGATGGCAGCGTAACGGAC
CR8_1               GCTCTTGCTTTGTGCTTCTGACCTATCAACTAGATGGCAGCGTAACGGAC
Fr_K7               GCTCTTGCTGTGTGCTTCTGACCTATCAACTAGATGGCAGCGTAAGGGAC
Fr_K15              GCTCTTGCTGTGTGCTTCTGACCTATCAACTAGATGGCAGCGTAAGGGAC
CR10                GCTCTTGCTGTGTGCTTCTGACCTATCAACTAGACGGCAGCGTAATGGAC
It_K61              GCTCTTGCGAAGTGCTTCTGACCTATCAACTAGACGGCAGCGTAACGGAC
Fr_K10              GCTCTTGCGAGGTGCTTCTGACCTATCAACTAGATAGCAGCGTAACGGAC
It_K66              GCTCTTGCGAGGTGCTTCTGACCTATCAACTAGATGGCAGCGTAACGGAC
Uk_K79              GCTCTTGCGAGGTGCTTCTGACCTATCAACTAGATGGCAGCGTAACGGAC
Pr3_1               GCTCTTGCGATGTGCTTCTGACCTATCAACTAGATGGCAGCGTAACGGAC
It_K71              GCTCTTGCGGTGTGCTTCTGACCTATCAACTAGACGGCAGCGTAAGGGAC
Fr_K18              GCTCTTGCTGTGTGCTTCTGACCTATCAACTAGACGGCAGCGTAAGGGAC
Fr_K62              GCTCTTGCGGTGTGCTTCTGACCTATCAACTAGACGGCAGCGTAAGGGAC
No_K94              GCTCTTGCAATGTGCTTCTGACCTATCAACTAGATGGCAGCGTAACGGAC
Cur_1               GCTCTTGCAAGGTGCTTCTGACCTATCAACTAGATGGCAGCGTAACGGAC
Az4_1               GCTCTTGCACGGCGCTTCTGACCTATCAACTAGATGGCAGCGTAAGGGAC
PR_1                GCTCTTGCAAGGTGCTTCTGACCTATCAACTAGATGGCAGCGTAACGGAC
Butricularis        GCTCTTGCTGTGTGCTTCTGACCTATCAACTAGATGGCAGCGTAACGGAC
NY_1                GCTCTAGCACGGTGCTTCTGACCTATCAACTAGATGGCAGCGTAACGGAC
Poblonga            GCTCTTGCAATGTGCTTCTGACCTATCAACTAGATGGCAGCGTAAGGGAC
Idn2                GGCCTTGCTGTGTGCTTCTGACCTATCAACTAGATGGCAGCGTAAAGGAC
CJ1_1               GGCCTTGCTGTGTGCTTCTGACCTATCAACTAGATGGCAGCGTAATGGAC
CR_1                GGCCTTGCTGTGTGCTTCTGACCTATCAACTAGATGGCAGCGTAAAGGAC
Pdidermoides        GGTCTTGCTGTGTGCTTCTGACCTATCAACTAGATGGCAGCGTAAAGGAC
UFF1                GCTCTTGCTGTGTGCTTCTGACCTATCAACTAGATGGCAGCGTAACGGAC
CJA3                GCTCTTGCAAGGTGCTTCTGACCTATCAACTAGATGGCAGCGTAACGGAC
Prigidum            GCTCTCGCTGTGTGCTTCTGACCTATCAACTAGATGGCAGCGTAAGGGAC
C1                  GCTCTCGCTTTGTGCTTCTGACCTATCAACTAGATGGCAGCGTAAGGGAC
PpolycephalumWis1   GCTCTCGCTGTGTGCTTCTGACCTATCAACTAGATGGCAGCGTAACGGAC
Sflavogenita        GCCCCTGCAACGGGCTTCTGACCTATCAACTAGACGGCAGCGTAAGGGAC

CUR1_4              ATGCTATGGTTACAACGGGTACAGAGGATAAGGGTTCGATCCTGGAGAGT
HA4_1               ATGCTATGGTTACAACGGGTACAGAGGATTAGGGTTCGATCCTGGAGAGT
CR19_1              ATGCTATGGTTACAACGGGTACAGAGGATAAGGGTTCGATCCTGGAGAGT
Pan2                ATGCTATGGTTACAACGGGTACAGAGGATAAGGGTTCGATCCTGGAGAGT
CR8_1               ATGCTATGGTTACAACGGGTACAGAGGATAAGGGTTCGATCCTGGAGAGT
Fr_K7               ATGCTATGGTGACAACGGGTACAGAGGATTAGGGTTCGATCCTGGAGAGT
Fr_K15              ATGCTATGGTGACAACGGGTACAGAGGATTAGGGTTCGATCCTGGAGAGT
CR10                ATGCTATGGTTACAACGGGTACAGAGGATTAGGGTTCGATCCTGGAGAGT
It_K61              ATGCCATGGTTACAACGGGTACAGAGGATTAGGGTTCGATCCTGGAGAGT
Fr_K10              ATGCCATGGTTACAACGGGTACAGAGGATTAGGGTTCGATCCTGGAGAGT
It_K66              ATGCCATGGTTACAACGGGTACAGAGGATTAGGGTTCGATCCTGGAGAGT
Uk_K79              ATGCCATGGTTACAACGGGTACAGAGGATTAGGGTTCGATCCTGGAGAGT
Pr3_1               ATGCTATGGTTGCAACGGGTACAGAGGATTAGGGTTCGATCCTGGAGAGT
It_K71              ATGCTATGGTTACAACGGGTACAGAGGATAAGGGTTCGATCCTGGAGAGT
Fr_K18              ATGCTATGGTTACAACGGGTACAGAGGATTAGGGTTCGATCCTGGAGAGT
Fr_K62              ATGCTATGGTTACAACGGGTACAGAGGATAAGGGTTCGATCCTGGAGAGT
No_K94              ATGCTATGGTTACAACGGGTACAGAGGATTAGGGTTCGATCCTGGAGAGT
Cur_1               ATGCTATGGTGACAACGGGTACAGAGGATAAGGGTTCGATCCTGGAGAGT
Az4_1               ATGCTATGGTGACAACGGGTACAGAGGATAAGGGTTCGATCCTGGAGAGT
PR_1                ATGCTATGGTGACAACGGGTACAGAGGATAAGGGTTCGATCCTGGAGAGT
Butricularis        ATGCTATGGTGACAACGGGTACAGAGGATAAGGGTTCGATCCTGGAGAGT
NY_1                ATGCTATGGTGACAACGGGTACAGAGGATAAGGGTTCGATCCTGGAGAGT
Poblonga            ATGCTATGGTGACAACGGGTACAGAGGATAAGGGTTCGATCCTGGAGAGT
Idn2                ATGCTATGGTGACAACGGGTACAGAGGATAAGGGTTCGATCCTGGAGAGT
CJ1_1               ATGCTATGGTGACAACGGGTACAGAGGATAAGGGTTCGATCCTGGAGAGT
CR_1                ATGCTATGGTGACAACGGGTACAGAGGATAAGGGTTCGATCCTGGAGAGT
Pdidermoides        ATGCTATGGTGACAACGGGTACAGAGGATAAGGGTTCGATCCTGGAGAGT
UFF1                ATCCTATGGTGACAACGGGTACAGAGGATAAGGGTTCGATCCTGGAGAGT
CJA3                ATGCTATGGTGACAACGGGTACAGAGGATAAGGGTTCGATCCTGGAGAGT
Prigidum            ATGCTATGGTAACAACGGGTACAGAGGATAAGGGTTCGATCCTGGAGAGT
C1                  ATGCTATGGTGACAACGGGTACAGAGGATAAGGGTTCGATCCTGGAGAGT
PpolycephalumWis1   ATGCCATGGTAACAACGGGTACAGAGGATAAGGGTTCGATCCTGGAGAGT
Sflavogenita        ATGCTATGGTAACAACGGGTACAGAGGATTAGGGTTCGATCCTGGAGAGT
```

```
CUR1_4              GGGCCTGAGAGATTGCTCATACTTCTAAGGAAGGCAGCAGGCGCGCAACG
HA4_1               GGGCCTGAGAGATTGCTCATACTTCTAAGGAAGGCAGCAGGCGCGCAACG
CR19_1              GGGCCTGAGAGATTGCTCATACTTCTAAGGAAGGCAGCAGGCGCGCAACG
Pan2                GGGCCTGAGAGATTGCTCATACTTCTAAGGAAGGCAGCAGGCGCGCAACG
CR8_1               GGGCCTGAGAGATTGCTCATACTTCTAAGGAAGGCAGCAGGCGCGCAACG
Fr_K7               GGGCCTGAGAGATTGCTCATACTTCTAAGGAAGGCAGCAGGCGCGCAACG
Fr_K15              GGGCCTGAGAGATTGCTTATACTTCTAAGGAAGGCAGCAGGCGCGCAACG
CR10                GGGCCTGAGAGATTGCTCATACTTCTAAGGAAGGCAGCAGGCGCGCAACG
It_K61              GGGCCTGAGAGATTGCTCATACTTCTAAGGAAGGCAGCAGGCGCGCAACG
Fr_K10              GGGCCTGAGAGATTGCTCATACTTCTAAGGAAGGCAGCAGGCGCGCAACG
It_K66              GGGCCTGAGAGATTGCTCATACTTCTAAGGAAGGCAGCAGGCGCGCAACG
Uk_K79              GGGCCTGAGAGATTGCTCAYACTTCTAAGGAAGGCAGCAGGCGCGCAACG
Pr3_1               GGGCCTGAGAGATTGCTCATACTTCTAAGGAAGGCAGCAGGCGCGCAACG
It_K71              GGGCCTGAGAGATTGCTCATACTTCTAGGGAAGGCAGCAGGCGCGCAACG
Fr_K18              GGGCCTGAGAGATTGCTCATACTTCTAAGGAAGGCAGCAGGCGCGCAACG
Fr_K62              GGGCCTGAGAGATTGCTCATACTTCTAAGGAAGGCAGCAGGCGCGCAACG
No_K94              GGGCCTGAGAGATTGCTCATACTTCTAAGGAAGGCAGCAGGCGCGCAACG
Cur_1               GGGCCTGAGAGATTGCTCACACTTCTAAGGAAGGCAGCAGGCGCGCAACG
Az4_1               GGGCCTGAGAGATTGCTCACACTTCTAAGGAAGGCAGCAGGCGCGCAACG
PR_1                GGGCCTGAGAGATTGCTCACACTTCTAAGGAAGGCAGCAGGCGCGCAACG
Butricularis        GGGCCTGAGAGATTGCTCACACTTCTAAGGAAGGCAGCAGGCGCGCAACG
NY_1                GGGCCTGAGAGATTGCTCACACTTCTAAGGAAGGCAGCAGGCGCGCAACG
Poblonga            GGGCCTGAGAGATTGCTCACACNTCTAAGGAAGGCAGCAGGCGCGCAACG
Idn2                GGGCCTGAGAGATTGCTCACACTTCTAAGGAAGGCAGCAGGCGCGTAACG
CJ1_1               GGGCCTGAGAGATTGCTCACACTTCTAAGGAAGGCAGCAGGCGCGTAACG
CR_1                GGGCCTGAGAGATTGCTCACACTTCTAAGGAAGGCAGCAGGCGCGTAACG
Pdidermoides        GGGCCTGAGAGATTGCTCACACTTCTAAGGAAGGCAGCAGGCGCGTAACG
UFF1                GGGCCTGAGAGATTGCTCACACTT-TAAGGAAGGCAGCAGGCGCGCAACG
CJA3                GGGCCTGAGAGATTGCTCACACTTCTAAGGAAGGCAGCAGGCGCGCAACG
Prigidum            GGGCCTGAGAGATTGCTCACACTTCTAAGGAAGGCAGCAGGCGCGCAACG
C1                  GGGCCTGAGAGATTGCTCACACTTCTAAGGAAGGCAGCAGGCGCGTAACG
PpolycephalumWis1   GGGCCTGAGAGATTGCTCACACTTCTAAGGAAGGCAGCAGGCGCGCAACG
Sflavogenita        GGGCCTGAGAGATTGCTCATACTTCTAAGGAAGGCAGCAGGCGCGCAACG

CUR1_4              TTCCCCTTGTGCAATGCATGAGGGCGTTAGGGGACATATGAATGCCTGCC
HA4_1               TTCCCCTTGTGCAATGCATGAGGGCGTTAGGGGACATATGAATGCCTGCC
CR19_1              TTCCCCTTGTGCAATGCATGAGGGCGTTAGGGGACATATGAATGTCTGCC
Pan2                TTCCCCTTGTACAATGTATGAGGGCGTTAGGGGACATATGAATGTCTGCC
CR8_1               TTCCCCTTGTACAATGTATGAGGGCGTTAGGGGACATATGAATGTCTGCC
Fr_K7               TTCCCATTGGACATCGTCCGAGGGCGTTAGGGGACATATGAATGTCTGCC
Fr_K15              TTCCCATTGGACATCGTCCGAGGGCGTTAGGGGACATATGAATGTCTGCC
CR10                TTCCCCTTGTGCAATGCATGAGGGCGTTAGGGGACATATGGATGCCTGCC
It_K61              TTCCCCTTGGGCAATGCCCGAGGGCGTTAGGGGACATATGAATGGTTGCC
Fr_K10              TTCCCCTTGGACAATGTCCGAGGGCGTTAGGGGACATATGAATGGTTGCC
It_K66              TTCCCCTTGGACAATGTCCGAGGGCGTTAGGGGACATATGAATGGTTGCC
Uk_K79              TTCCCCTTGGACAATGTCCGAGGGCGTTAGGGGACATATGAATGGTTGCC
Pr3_1               TTCCCCTTGGACAATGTTCGAGGGCGTTAGGGGACATATGAATGCTTGCC
It_K71              TTCCCCTTGGGCAATGCTCGAGGGCGTTAGGGGACATATGAATGCCTGCC
Fr_K18              TTCCCCTTGGGCAATGCCCGAGGGCGTTAGGGGACATATGAATGCCTGCC
Fr_K62              TTCCCCTTGGGCAATGCTCGAGGGCGTTAGGGGACATATGAATGCCTGCC
No_K94              TTCCCCTTGGGCAATGCCCGAGGGCGTTAGGGGACATATGAATGCCTGCC
Cur_1               TTCCCCTTGGGCAATGCTCGAGGGCGTTAGGGGACATATGAATGCCTGCC
Az4_1               TTCCCCTTGGGCAATGCTCGAGGGCGTTAGGGGACATATGAATGTCTGCC
PR_1                TTCCCCTTGGGCAATGCTCGAGGGCGTTAGGGGACATATGAATGCTTGCC
Butricularis        TTCCCATTGGGCAAAGCTCGAGGGCGTTAGGGGACATATGAATGCCTGCC
NY_1                TTCCCATTGGACAATGTCCGAGGGCGTTAGGGGACATATGAATGTCTGCC
Poblonga            TTCCCATTGGACTCCGTCCGAGGGCGTTAGGGGAAATATGAATGTCTGCC
Idn2                TTCCCATTGGGCAAAGCCTGAGGGCGTTAGGGGACATATGAATGCTTGCC
CJ1_1               TTCCCATTGGGCAAAGCCTGAGGGCGTTAGGGGACATATGAATGCTTGCC
CR_1                TTCCCATTGGGCAAAGCCTGAGGGCGTTAGGGGACATATGAATGCTTGCC
Pdidermoides        TTCCCATTGGGCAAAGCCTGAGGGCGTTAGGGGACATATGAATGCTTGCC
UFF1                TTCCCATTGGGCAAAGCTCGAGGGCGTTAGGGGACATATGAATGCCTGCC
CJA3                TTCCCCTTGGGCAATGCTCGAGGGCGTTAGGGGACATATGAATGCCTGCC
Prigidum            TTCCCATTGGGCACAGCTCGAGGGCGTTAGGGGACATATGAATGCCCATC
C1                  TTCCCCTTGGGCAATGCTCGAGGGCGTTAGGGGACATATGAATGCCTGCC
PpolycephalumWis1   TTCCCATTGGGCAAAGCTCGAGGGCGTTAGGGGACATATGAATGCCTGCC
Sflavogenita        TTCCCCTTGGGCAAAGCTCGAGGGCGTTATGGGAAATATGAATGCTTGCC
```

```
CUR1_4              CTTTGGGTGGGCAATTCAAATGGGTCTGTTGTAAACAGGCTCTCGAGTAA
HA4_1               CAATGGGTGGGCAATTCAAATGGGTCTGTTGTAAACAGGCTCTCGAGTAA
CR19_1              CTATGGGTGGACAATTCAAATGGGTCTGTTGTAAACAGGCTCTCGAGTAA
Pan2                CTATGGGTGGACAATTCAAATGGGTCTGTTGTAAACAGGCTCTCGAGTAA
CR8_1               CTATGGGTGGACAATTCAAATGGGTCTGTTGTAAACAGGCTCTCGAGTAA
Fr_K7               CATTGGGTGGACAATTCAAATGGGTCTGTTCTAAACAGGCTCTCGAGTAA
Fr_K15              CATTGGGTGGACAATTCAAATGGGTCTGTTCTAAACAGGCTCTCGAGTAA
CR10                CAATGGGTGGGCAATTCAAATGGGTCTGTTGTAAACAGGCTCTCGAGTAA
It_K61              TTTTAGGTGGCCAATTCAAATGGGTCTGTTCTAAACAGTCTCTCGAGTAA
Fr_K10              TTTTAGGTGGCCAATTCAAATGGGTCTGTTCTAAACAGTCTCTCGAGTAA
It_K66              TTTTAGGTGGCCAATTCAAATGGGTCTGTTCTAAACAGTCTCTCGAGTAA
Uk_K79              TTTTAGGTGGCCAATTCAAATTGGTCTGTTCTAAACAGTCTCTCGAGTAA
Pr3_1               ATT--GGTAGGCAATTCAAATGGGTCTGTTCTAAACAGCCTCTCGAGTAA
It_K71              ATATTGGTGGGCAATTCAAATGGGTTTGTTCTAAACAGCCTTTCGAGTAA
Fr_K18              ATATTGGTGGGCAATTCAAATGGGTCTGTTCTAAACAGCCTCTCGAGTAA
Fr_K62              ATATTGGTGGGCAATTCAAATGGGTTTGTTCTAAACAGCCTTTCGAGTAA
No_K94              CTATGGGTGGGCAATTCAAATGGGTTTGTTGTAAACAGCCCTTCGAGTAA
Cur_1               ATTT-GGTGGGCAATTCAAATGGGTTCGTTCTAAACAGCCCTTCGAGTAA
Az4_1               TTTT-GGTGGACAATTCAAATGGGTCTGTTCTAAACAGCCTCTCGAGTAA
PR_1                ATTT-GGTGGGCAATTCAAATGGGTTCGTTCTAAACAGCCCTTCGAGTAA
Butricularis        TTAT-GGTGGGCAATTCAAATGGGACTGTTTTAAACATCCTATCGAGTAA
NY_1                TTAT-GGTGGACAATTCAAATGGGTTTGTTCTAAACAACCTTTCGAGTAA
Poblonga            TTAT-GGTGGACAATTCAAATGGGACTGTTCTAAACAGCCCATCGNGTAA
Idn2                ATTT-GGTAGGCAATTCAAATGGGTTTGTTCTAAACAGCCCTTCGAGTAA
CJ1_1               ATAT-GGTAGGCAATTCAAATGGGTTCGTTCTAAACAACCCTTCGAGTAA
CR_1                ATAT-GGTAGGCAATTCAAATGGGTTCGTTCTAAACAACCCTTCGAGTAA
Pdidermoides        TTAT-GGTAGGCAATTCAAATGGGTTTGTTCTAAACAGCCTATCGAGTAA
UFF1                TTT--GGTGGGCAATTCAAATGGGACTGTTTTAAACATCCTATCGAGTAA
CJA3                ATTT-GGTGGGCAATTCAAATGGGTTCGTTCTAAACAGCCCTTCGAGTAA
Prigidum            TTAATGGTGGGTAATTCAAATGGGGCCGTTTTAAATATCCTATCGAGTAA
C1                  TTTT-GGTGGGCAATTCAAATGGGTCTGTTCTAAACAGCCTCTCGAGTAA
PpolycephalumWis1   TTAT-GGTGGGCAATTCAAATGGGACTGTTTTAAACATCCTATCGAGTAA
Sflavogenita        TTTACGGTGGGCAATTCAAATGGGTTTGTCGTAAACAGGCTCTCGAGTAA

CUR1_4              CAATTAGAGGACAAGTCTGGTGCCAGCACCCGCGGTAATTCCAGCTCTAA
HA4_1               CAATTAGAGGACAAGTCTGGTGCCAGCACCCGCGGTAATTCCAGCTCTAA
CR19_1              CAATTAGAGGACAAGTCTGGTGCCAGCACCCGCGGTAATTCCAGCTCTAA
Pan2                CAATTAGAGGACAAGTCTGGTGCCAGCACCCGCGGTAATTCCAGCTCTAA
CR8_1               CAATTAGAGGACAAGTCTGGTGCCAGCACCCGCGGTAATTCCAGCTCTAA
Fr_K7               CAATTAGAGGACAAGTCTGGTGCCAGCACCCGCGGTAATTCCAGCTCTAA
Fr_K15              CAATTAGAGGACAAGTCTGGTGCCAGCACCCGCGGTAATTCCAGCTCTAA
CR10                CAATTAGAGGACAAGTCTGGTGCCAGCACCCGCGGTAATTCCAGCTCTAA
It_K61              CAATTAGAGGACAAGTCTGGTGCCAGCACCCGCGGTAATTCCAGCTCTAA
Fr_K10              CAATTAGAGGACAAGTCTGGTGCCAGCACCCGCGGTAATTCCAGCTCTAA
It_K66              CAATTAGAGGACAAGTCTGGTGCCAGCACCCG---TAATTCCAGCTCTAA
Uk_K79              CAATTAGAGGACAAGTCTGGT-CCAGCACCCGCGGTAATTCCAGCTCTAA
Pr3_1               CAATTAGAGGACAAGTTTGGTGCCAGCACCCGCGGTAATTCCAGCTCTAA
It_K71              CAATTAGAGGACAAGTCTGGTGCCAGCACCCGCGGTAATTCCAGCTCTAA
Fr_K18              CAATTAGAGGACAAGTCTGGTGCCAGCACCCGCGGTAATTCCAGCTCTAA
Fr_K62              CAATTAGAGGACAAGTCTGGTGCCAGCACCCG-GGTAATTCCAGCTCTAA
No_K94              CAATTAGAGGACAAGTCTGGTGCCAGCACCCGCGGTAATTCCAGCTCTAA
Cur_1               CAATTAGAGGACAAGTCTGGTGCCAGCACCCGCGGTAATTCCAGCTCTAA
Az4_1               CAATTAGAGGACAAGTCTGGTGCCAGCACCCGCGGTAATTCCAGCTCTAA
PR_1                CAATTAGAGGACAAGTCTGGTGCCAGCACCCGCGGTAATTCCAGCTCTAA
Butricularis        CAATTAGAGGACAAGTCTGGTGCCAGCACCCGCGGTAATTCCAGCTCTAA
NY_1                CAATTGGAGGACAAGTCTGGTGCCAGCACCCGCGGTAATTCCAGCTCTAA
Poblonga            CAATTAGAGGACAAGTCTGGTGCCAGCACCCGCGGTAATTCCAGCTCTAA
Idn2                CAATTAGAGGACAAGTCTGGTGCCAGCACCCGCGGTAATTCCAGCTCTAA
CJ1_1               CAATTAGAGGACAAGTCTGGTGCCAGCACCCGCGGTAATTCCAGCTCTAA
CR_1                CAATTAGAGGACAAGTCTGGTGCCAGCACCCGCGGTAATTCCAGCTCTAA
Pdidermoides        CAATTAGAGGACAAGTCTGGTGCCAGCACCCGCGGTAATTCCAGCTCTAA
UFF1                CAATTAGAGGACAAGTCTGGTGCCAGCACCCGCGGTAATTCCAGCTCTAA
CJA3                CAATTAGAGGACAAGTCTGGTGCCAGCACCCGCGGTAATTCCAGCTCTAA
Prigidum            CAATTAGAGGACAAGTCTGGTGCCAGCACCCGCGGTAATTCCAGCTCTAA
C1                  CAATTAGAGGACAAGTCTGGTGCCAGCACCCGCGGTAATTCCAGCTCTAA
PpolycephalumWis1   CAATTAGAGGACAAGTCTGGTGCCAGCACCCGCGGTAATTCCAGCTCTAA
Sflavogenita        CAATTAGAGGACAAGTCTGGTGCCAGCACCCGCGGTAATTCCAGCTCTAA
```

```
CUR1_4              TAGCATACGTTAAAGTTGTTGCGGTTAAAACGCTCGTAGTCGGCTTCAGA
HA4_1               TAGCATACGTTAAAGTTGTTGCGGTTAAAACGCTCGTAGTCGGCTACAGA
CR19_1              TAGCATACGTTAAAGTTGTTGCGGTTAAAACGCTCGAAGTCGGCATAAGA
Pan2                TAGCATACGTTAAAGTTGTTGCGGTTAAAACGCTCGTAGTCGGCTTAAGG
CR8_1               TAGCATACGTTAAAGTTGTTGCGGTTAAAACGCTCGTAGTCGGCTTAAGG
Fr_K7               TAGCATACGTTAAAGTTGTTGCGGTTAAAACGCTCGTAGTCGGCCTGAGA
Fr_K15              TAGCATACGTTAAAGTTGTTGCGGTTAAAACGCTCGTAGTCGGCCTGAGA
CR10                TAGCATACGTTAAAGTTGTTGCGGTTAAAACGCTCGTAGTCGGCTTCAGA
It_K61              TAGCATACGTTAAAGTTGTTGCGGTTAAAACGCTCGTAGTCGGCTTCAGA
Fr_K10              TAGCATACGTTAAAGTTGTTGCGGTTAAAACGCTCGTAGTCGGCTTAAGA
It_K66              TAGCATACGTTAAAGTTGTTGCGGTTAAAACGCTCGTAGTCGGCTTAAGA
Uk_K79              TAGCATACGTTAAAGTTGTTGCGGTTAAAACGCTCGTAGTCGGCTTAAGA
Pr3_1               TAGCGTACGTTAAAGTTGTTGCGGTTAAAACGCTCGTAGTCGGCTTCAGA
It_K71              TAGCATACGTTAAAGTTGTTGCGGTTAAAACGCTCGTAGTCGGCTTAAGA
Fr_K18              TAGCATACGTTAAAGTTGTTGCGGTTAAAACGCTCGTAGTCGGCTTAAGA
Fr_K62              TAGCATACGTTAAAGTTGTTGCGGTTAAAACGCTCGTAGTCGGCTTAAGA
No_K94              TAGCATACGTTAAAGTTGTTGCAGTTAAAACGCTCGTAGTCGGCTTCAGA
Cur_1               TAGCATACGTTAAAGTTGTTGCGGTTAAAACGCTCGTAGTCGGCGCCAGA
Az4_1               TAGCATACGTTAAAGTTGTTGCGGTTAAAACGCTCGTAGTCGGCGCCAGA
PR_1                TAGCATACGTTAAAGTTGTTGCGGTTAAAACGCTCGTAGTCGGCGCCAGA
Butricularis        TAGCATACGTTAAAGTTGTTGCGGTTAAAACGCTCGTAGTCGGCCACAGA
NY_1                TAGCATACGTTAAAGTTGTTGCGGTTAAAACGCTCGTAGTCTGCATAAGA
Poblonga            TAGCATACGTTAAAGTTGTTGCAGTTAAAACGCTCGTAGTCGGCACGAGA
Idn2                TAGCATACGTTAAAGTTGTTGCGGTTAAAACGCTCGTAGTCGGCGCCAGA
CJ1_1               TAGCATACGTTAAAGTTGTTGCGGTTAAAACGCTCGTAGTCGGCGCCAGA
CR_1                TAGCATACGTTAAAGTTGTTGCGGTTAAAACGCTCGTAGTCGGCGCCAGA
Pdidermoides        TAGCATACGTTAAAGTTGTTGCGGTTAAAACGCTCGTAGTCGGCAACAGA
UFF1                TAGCATACGTTAAAGTTGTTGCGGTTAAAACGCTCGTAGTCGGCAACAGA
CJA3                TAGCATACGTTAAAGTTGTTGCGGTTAAAACGCTCGTAGTCGGCGCCAGA
Prigidum            TAGCATACGTTAAAGTTGTTGCGGTTAAAACGCTCGTAGTCGGCTCCAGA
C1                  TAGCATACGTTAAAGTTGTTGCGGTTAAAACGCTCGTAGTCGGCTTAAGA
PpolycephalumWis1   TAGCATACGTTAAAGTTGTTGCGGTTAAAACGCTCGTAGTCGGCTCCAGA
Sflavogenita        TAGCATACGTTAAAGTTGTTGCGGTTAAAACGCTCGTAGTCGGCTTCAGA

CUR1_4              TCATCAGATGCCTGAGCTGCAATCCCGGGTCAGC-T--GCCTTTCGGGGC
HA4_1               CCATCAGAAGCCTGACCTGTGACCCCGGGACCGC-T--GCCTTTCGGGGC
CR19_1              TCGTCAGAATCCCGATCTGCAAACCTTGCTT-GT-T--GCCTTTCGGGGC
Pan2                CCATCAGATTCCTGATCTGCGAGTCCGGGTCCGC-T--GCCTTTCGGGGC
CR8_1               CCATCAGATTCCTGATCTGCGAGTCCGGGTCCGC-T--GCCTTTCGGGGC
Fr_K7               CCATCAGATGCCTGAGCAGGGAACCCGGGACTG--T--GCCTTTCGAGGT
Fr_K15              CCATCAGATGCCTGAGCGGGGAACCCGGGACTG--T--GCCTTTCGAGGT
CR10                TCATCAGATGCCAGAGTCGTAATCTCGGGTCCGC-T--GCCCTTCGGGGC
It_K61              TCATCAAACGCCTGAGACGGG--AACCTGGCTGC-T--GCCTTCAAAGGC
Fr_K10              TCATCAGAAGCTTGCGATGGA--ACCCTGGATGC-T--GCCTTCAATGGT
It_K66              TCATCAGAAGCTTGCGATGGA--ACCCTGGATGC-T--GCCTTCAATGGT
Uk_K79              TCATCAGAAGCTTGCGATGGA--ACCCTGGATGC-T--GCCTTCAATGGT
Pr3_1               TCGTCACGAAGCCGGAACGGGCCGGTCGGATTGT-C--GCCTC--AAGGC
It_K71              TCATCAGATGCCTGATCCGGGAGCCTGGGACCGC-T--GCTCTTCGGAGC
Fr_K18              TCGTCAGACGCCCGATCCGGGAGTCCAGGCCTGC-T--GCTCTTCGGAGC
Fr_K62              TCATCAGATGCCTGATCCGGGAGCCTGGGACCGC-T--GCTCTTCGGAGC
No_K94              CCGTCAGAAGCCTAAGCTGGGAATCCGGATCCGTGT--CCTTCACTGGGC
Cur_1               TCTTCAGAATCCAGCGTTGGGGACC-GG-TTCGTTGCCCTGGTCAAGGGC
Az4_1               TCTTCAGAGTTCTGCGTTGGGACCT-GGGNNNGNTGCTCCGGTCAAGGGC
PR_1                TCTTCAGAATCCAGCGTTGGGGACC-GG-TTCGTTGCCCTGGTCAAGGGC
Butricularis        CCTTCAAAAGCTTGATTCGGGAAACCGGGCCTGCTGTCTTCTCCAAGGGC
NY_1                TCCTCAAAGCCTTTCGCTAGGTCCTTAGGTTCGTCGCCCTGCCAAAGGGC
Poblonga            CCTTCAATGCTTCGGATCGTGATCCT--GTTTGCTGCTTTCTCAA-GCGT
Idn2                TCTTCAGAACCCTGATACGCGAAGC-GGGTCAGCTGCCCCGGTCAAGGGC
CJ1_1               TCTTCAGAACCTCGAGACACAAAGC-AGGTCAGTCGCTCCTGTCAAGGGC
CR_1                TCTTCAGAACCTTGAGACACGAAGC-AGGTCAGTCGCTCC-GTCAAGAGC
Pdidermoides        TCTTCAGAACCTCGATTCACGAAGC-GAGTCAGCTGCCCCGGTCAAGGGC
UFF1                TCGTCGTAACCTCGATTCGGGACCCTGGGCCAGCTGTTCTTTCTCGGAAC
CJA3                TCTTCAGAATCCAGCGTTGGGGACC-GG-TTCGTTGCCCTGGTCAAGGGC
Prigidum            CCTTCAGAAGCCTGAAGCGGGACGCTGGGTCAGCTATTTTCTCCGAGGAC
C1                  TCTTCAGATTCTTGCGCTGGGAGCC-GGGTCCGTTGCCCTCGTAAAGGGC
PpolycephalumWis1   CCTTCAGAGCCTTGATTCGGGACACTGGGTCAGCTGTCTTCTCCAAGAGC
Sflavogenita        CCATCAGAAGCCTGGCTCGGGGTCTCGGGTCTGTGC--CTCGCAAGGGGT
```

```
CUR1_4              GCGCTGGGGCC-GTGGCCGAGGGTGGGCGGTGGTCAAGCGTGCCTCTGGC
HA4_1               GCGCTGG-GAG-AACGGAGACGGTGGGCGGTGGTCAAGTGTACCTTTGGC
CR19_1              GCACTGG-GCC-AGTGGAGAGGGAGGGCGGCGGTCAAGCGTATCCTTGGC
Pan2                GCGCTGG-GCG-AGTGGTGATGGAGGGCGGTGGCCAAGCGTGCCTCTGGT
CR8_1               GCGCTGG-GCG-AGTGGTGATGGAGGGCGGTGGCCAAGCGTGCCTCTGGT
Fr_K7               GCGCTGG-GCG-ACTAACAAGGGCGGGCGGTAGTCAAGTGTGCCGTAGGC
Fr_K15              GCGCTGG-GCCTACTAGCAAGGGCGGGCGGTAGTCAAGTGTGCCGTAGGC
CR10                GCGCTGG-GTG-CGTGACGATGGCGGGCGGTGGTCAAGCGTGCCTCCGGT
It_K61              GCTCCGG-GCG-ACTAGCGAAGGCTGACGGTGGTCAAGTGTGCCTCAGGC
Fr_K10              GCTCTGG-GCG-ACTGACAAAGGCTGGCGGTGATCAAGCGTGCCTTAGGC
It_K66              GCTCTGG-GCG-ACTGACAAAGGCTGGCGGTGATCAAGCGTGCCTTAGGC
Uk_K79              GCTCTGG-GCG-ACTGACAAAGGCTGGCGGTGATCAAGCGTGCCTTAGGC
Pr3_1               GCGCCGG-CTG-GTCTGTGACGGTTGACGGCGGTCAAACGTGCCTCAGGC
It_K71              GCGCCAG-GTA-ACTAGAGAGGGTGGGCGGTGGTCAAGTGTGCCTTGGGC
Fr_K18              GCGCTGG-ATG-ACTAGAGAGGGTTGGCGGCGGTCAAGTGTGCCTTGGGC
Fr_K62              GCGCCAG-GTA-ACTAGAGAGGGTGGGCGGTGGTCAAGTGTGCCTTGGGC
No_K94              TGGCCGG-AGG-ACTGGCGAGGGTAGGCGGCGGTCAAGTGTGCCCCGGTT
Cur_1               ACGCTGGCGACCG--ACAACGGA-GGACGTCGGTCAACAGTGCCCGTGGC
Az4_1               ACGCCAGGAACTG--ACAAGGGA-GGACGTCGATCAAACGTACTCGTGGC
PR_1                ACGCTGGCGACCG--ACAACGGA-GGACGTCGGTCAACAGTGCCCGTAGC
Butricularis        GCGCCGGCTACTG--CTGATAGT-GGACGTCGGTCAAGTGTGCTCCAGGC
NY_1                ACACCTGGGGGTC-AGCAAGGGGGGGACGGGGGTCAACCGTACACTGGGC
Poblonga            GCATCGGAGGATCGAACTGGAGTTTGACGTCGGTCGCATGTGCCTAGGGC
Idn2                GCGCCGTGGAGTA--TTGAGGGG-GGACGTCGGTCAAACGCTTCTCGCTT
CJ1_1               ACGCTGTGGAGGG--TCGAGGGGCAGACGTCGATCAAACGCTTCGTGGTT
CR_1                ACGCTGTGGAGGG--TTGAGAGGCAGACGTCGATCAAACGCTTCGTGGTT
Pdidermoides        GCGTCGTGGAGAG--TTGAGAGGCAGACGTCGATCAAACGCTTCCTGGTT
UFF1                GCGCCAGCTACTG--ATGACGGG-AGACGTCGGTCAACTGCGCCCCAGGC
CJA3                ACGCTGGCGACCG--ACAACGGA-GGACGTCGATCAACAGTACCCGTAGC
Prigidum            GCGCCAGTTACTG--CCGAGGGT-GGACGTCGGTCAAGCGTGTCTCAAGC
C1                  ACGCCGGGGACTG--GCAACGGA-GGACGTTGGTCGAACGCATTTCAGGC
PpolycephalumWis1   GCGCCAACTACTG--ATGATAGG-GGACGTCGGTCAATCGTGCCCTGGGC
Sflavogenita        GCGCCTT--GGTCTTGGGCCGGGTGGGCGGCGGTCAATCGTGCCCAGTGG

CUR1_4              TGTCCTTCGACAG-CTCGGCTGGGGGTGCCACTTACCATGATTAAACTGG
HA4_1               TGTCCTTCGACAG-TTCGGCTGGGGGTACCGATCAGATACCCCAAACTGG
CR19_1              TGTCTTTCGACAG-CTCGGCTGGGGGTACCAATTACCATGACTAAACTGG
Pan2                CGTCTTTCGACAG-CTCGGCTGGGGGTGCCACTTACCATGATTAAACTGG
CR8_1               CGTCTTTCGACAG-CTCGGCTGGGGGTGCCACTTACCATGATTAAACTGG
Fr_K7               TGTACCCTCACAGATTCGGCTTGCGGTACCACTTACCATAATCAAACTGG
Fr_K15              TGTACCCTCACAGATTCGGCTTGCGGTACCACTTACCATAATCAAACTGG
CR10                CGTCCTTCGACAG-TCTGGCTGGGGGTGCCACTTACCATGACCAAACTGG
It_K61              TGTGTCCTCACAGGTTCGGCTTGTGGTACCACTTACCATGACTAAACCGG
Fr_K10              TGTATCTTCACAGGTTCGGCTTGGGGTGCCACTTACCATGATCAAACCGG
It_K66              TGTATCTTCACAGGTTCGGCTTGGGGTGCCACTTACCATGATCAAACCGG
Uk_K79              TGTATCTTCACAGGTTCGGCTTGGGGTGCCACTTACCATGATCAAACCGG
Pr3_1               TGTCCTTCGACAGGCTCGGCTTGGGGTGCCACTTACCATGACTAAACCGG
It_K71              TGCCTCTCCGCAGGTCCGGCCTGGGGTACCATTTACTATGATTAAACTGG
Fr_K18              TGCCTCCTTGCAGGTTCGGCCTGAGGTGCCAGTTACCATGATTAAACTGG
Fr_K62              TGCCTCTCCGCAGGTCCGGCCTGGGGTACCATTTACTATGATTAAACTGG
No_K94              CCTCTCTCCACAGGCTCGGACCGAGGTACCACTTACCATGATCAAACTGA
Cur_1               TGCTCCCTCGCAGGTCCGGCTCGGG-TGCCATCTACCATGATTAAACCGG
Az4_1               TGCTCCCCTGCAGGTCCGGTCCGGG-TACCGTTTACCATGACTAAACCGG
PR_1                TGCTCCCTTGCAGGTCCGGCTCGGG-TGCCATCTACCATGATTAAACCGG
Butricularis        TGTCTCCTCACAGGTTCGGCTTGGGGTGCCTATCACCATGATTAAACCGT
NY_1                CGTCTCCCGACAGGTCTGGTCTGGG-TACCATCTACCATGACTAAACTGG
Poblonga            CACTCTCCGAAAAGCCCGGTCCGCG-CGCCATTTACCATGATTAAACTGG
Idn2                CACCTTCGGAAAGGTCTGGAGCGGGTTGCCATTCACTATGATTAAACTAT
CJ1_1               CACTCCTTCAAAAGCCTGGACTGCGTTGCCACTCACTATGATTAAACTAT
CR_1                CACTCCTCCAAAAGCCTGGACTGCGTCGCCACTCACTATGATTAAACTAT
Pdidermoides        CACTCCTTCAAAAGTCTGGACTGGGTCGCCAATCACTATGATTAAACTAT
UFF1                TGTCTTCTTACAGGCTCGGCTTGGGGCGCCCATCACCATGATCAAACCGT
CJA3                TGCTCCCTCGCAGGTCCGGCTCGGG-TGCCATCTACCATGATTAAACCGG
Prigidum            TGTTGCCCCACAGGCTCGGCTTGGGTCACCTATCACCATGATTAAACTGT
C1                  TGCCTCCTCGCAGGTTCGGCCTGGA-TGCCATTTACCATGATTAAACCGG
PpolycephalumWis1   TGTCTCCCCACAGGCTCGGCTCGGGGTACCAATCACCATGATTAAACCGT
Sflavogenita        CGTGTCGCGACAGGTCTGGCTCTGGGTACCAATTACCATGATTAAACCGG
```

```
CUR1_4             GTTGATCAAGTAAGGTCTTTCGA-TCT-TGCACAGCACAGCATGGTATAA
HA4_1              GTTGATCAAGTAAGGTCGTTCGA-TCT-TGCACAGCACAGCATGGCATAA
CR19_1             GTTGATCAAGTAAGGCCATTCGG-TCT-TGCACAGCACAGCATGGTATAA
Pan2               GTTGATCAAGTAAGGTCGTTCGAATCT-TGCACAGCACAGCATGGTATAA
CR8_1              GTTGATCAAGTAAGGTCGTTCGAATCT-TGCACAGCACAGCATGGTATAA
Fr_K7              GGTGATCAAAGCAGGTCGTCAGA-CCT-TGCACAGCACAGTATGGCGTAA
Fr_K15             GGTGATCAAAGCAGGTCGTCAGA-CCT-TGCACAGCACAGTATGGCATAA
CR10               GTTGATCAAGTAAGGTCGTTCGA-TCT-TGCACAGCACAGCATGGCATAA
It_K61             CTCGATCAAGAATGGTCACGACGATCT-TGTACGGCGCAGCATGGCATAA
Fr_K10             CTCGATCAAGAAAGGTCACGACGATCT-TGCACGGCTTAGCATGGAATAA
It_K66             CTCGATCAAGAAAGGTCACGACGATCT-TGCACGGCTTAGCATGGAATAA
Uk_K79             CTCGATCAAGAAAGGTCACGACGATCT-TGCACGGCTTAGCATGGAATAA
Pr3_1              ACCGATTAAGAGAGGTCTCT-CGATCT-TGTACGGCTCAGCATGGGATAA
It_K71             AGTGATCAAAGCAGGTCGATCGA-CCT-TGCACAGCACAGCATAGCATAA
Fr_K18             GGTGATCAAAGCAGGTCGTCAGA-CCT-TGCACAGCACAGCATGGCACAA
Fr_K62             AGTGATCAAAGCAGGTCGATCGA-CCT-TGCACAGCACAGCATAGCATAA
No_K94             GGTGATCAAAGCAGGTCATTCGA-CCT-TGCACAGCACAGCATGGCATAA
Cur_1              GGTGATCAAAGCAGGTCT-TT-CGACA-CGTACGGCGCAGCATGGTATAT
Az4_1              GGTGATCAAAGCAGGTCT-TT-CGACA-CGTACGGCGCAGCATGGCATAA
PR_1               GGTGATCAAAGCAGGTCT-TT-CGACA-CGTACGGCGCAGCATGGTATAT
Butricularis       AGTGACCAAAGCACGTCG-TT-AGACG-GGCACGGCACAGCATGGCACGA
NY_1               AGTGATCAAAGCATGTC--TTTCGACA-CGTACAGCGCAGCATGGA----
Poblonga           GGTGATCGAAGCATGACGCTTACGTCG-CGTACAGGATAGCATGGGT---
Idn2               AGTGATCAAAGCGGGTCA-TTACGACA-CGCATAGCACAGCATAGTATGA
CJ1_1              AGTGATCAAAGCGGGTCA-TT-CGACA-CGCATAGCACAGCATAGTATGA
CR_1               AGTGATCAAAGCGGGTCA-TT-CGACA-CGCATAGCACAGCATAGTATGA
Pdidermoides       AGTGATCAAAGCGGGTCA-TTACGACA-CGCATAGCACAGCATAGTATGA
UFF1               AGTGATCGAGGCACGTCG-CA-AGACG-GGCACGGCACAGCATGGGATGA
CJA3               GGTGATCAAAGCAGGTCT-TT-CGACA-CGTACGGCGCAGCATGGTATAT
Prigidum           AGTGATCAAAGCACGTCT-TT-CGATG-GGTGCAGCGTAGCATGGTACGG
C1                 GGTGATCAAAGCAGGTCT-TT-CGACA-CGTACGGCGCAGCATGGTATAC
PpolycephalumWis1  AGTGACCAAAGCACGTCT-TT-AGACG-GGCACGGCACAGCATGGGACGA
Sflavogenita       AGTGATCAAAGCAAACCGCTGCGGTTACTGCACGGATTAGCATGGCATAA

CUR1_4             G-TCTAGCCTGTTCGGTCCTTCCTC-GTGGGGGC-TGGGTGG-GCAGAAG
HA4_1              A-ACTAACCGGTCAGGCCTTCCCTC-GTGGGGGT-CGGGCTG-GTAAAAG
CR19_1             A-ATCTGCCTGTTTGA-CCTCTCTC-GTGGGGGT-CAGGTGG-GTAAAAG
Pan2               A-TTTAGCCTGTTTGGTCCTTCCTC-GCGGGGGC-TGGGTGG-GCAGAAG
CR8_1              A-TTTAGCCTGTTTGGTCCTTCCTC-GCGGGGGC-TGGGTGG-GCAGAAG
Fr_K7              G-TCTTACTGGCCGG-----CACTT-GCGAGGTG-CCGGTCG-GTAGAAG
Fr_K15             G-TCTTACTGGCCGG-----CACTT-GCGAGGTG-CCGGTCG-GTAGAAG
CR10               A-GGTAACTGGCCTGGTCCTTCTCG-CGAGGGGC-TGGGCTG-GTAAAAG
It_K61             G-CATAACTGGTCCGG-GATT-TCT-TGCGGTCC-TGGATCG-GTAGAAG
Fr_K10             G-TTAAACTGGCCCGG-GATT-CTC-GCGGATCC-TGGGTTG-GTAGAAG
It_K66             G-TTAAACTGGCCCGG-GATC-CTC-GCGGATCC-TGGGTTG-GTAGAAG
Uk_K79             G-TTAAACTGGCCCGG-GATT-CTC-GCGGATCC-TGGGTTG-GTAGAAG
Pr3_1              T-TAACACTGGTCCGG-GGCTACTT-GCGGGCCC-CGGGTTG-GTAAAAG
It_K71             G-CAAAACTGGCTTGG--CCCTATT-ACGGGGGC-TGGGTTG-GTAGAAG
Fr_K18             G-CGAAACCGGTCTGG--CTCTATT-ACGGGGGC-TGGGCTG-GTAGTAG
Fr_K62             G-CAAAACTGGCTTGG--CCCTATT-ACGGGGGC-TGGGTTG-GTAGAAG
No_K94             G-CCAAACCGGCTCGGGGCTCCCTC-GCGGGTCC-CGGGTCG-GTAGAAG
Cur_1              --CTTCACCGGTCTTGCCCTAGTT--GCGGGGGC-TTGGCTG-GTAAGAG
Az4_1              --GCCCACTGGCCTTGCCCTATTT--GCGGGGGC-TTGGCTG-GTAAAGG
PR_1               --CTTCACCGGTCTTGCCCTAGTT--GCGGGGGC-TTGGCTG-GTAAGAG
Butricularis       A-TCGCACCGGGCTCGCCTTTTTTTTGCGGGGGCTGAGACTCGGTAAAAG
NY_1               --ATAAGCTCACCCATCT---GGCCCGCGAAA---CGGGCTG-GCGAGCA
Poblonga           --ATAAGCTCGACTGGCTTGGGAGTTGCGAAC---CAGGCTA-GTAATCG
Idn2               --TCAAGCTGGCCGAGCCCGAGTT--GCA-AGGGCTGAGCTG-GCAGGCG
CJ1_1              --TCAAGCTGGCCGAGCCCTCGTT--GCGTGGGGCTGAGCTG-GCAGGCG
CR_1               --TCAAGCTGGCCGAGCCTTCGTT--GCATGGGGCTGAGCTG-GCAGGCG
Pdidermoides       --ACAAGCTGGCCGAGTCCTAGTT--GCG-GGGATTGAGCTAAGCAGTTG
UFF1               A-AATAACCGGGCTCGCTTCGTTTT-GCGGGAGCAGAGACCTGGTAAACG
CJA3               --CTTCACCGGTCTTGCCCTAGTT--GCGGGGGC-TCGGCTG-GTAAGAG
Prigidum           G-GCA-ACCCAGCTCACCTTATTTT-GCGGGGGA-GAGACTGGGTAGATG
C1                 --TCTAACCGGCCGGGCTCTA-TT--GCGGGGGC-CCGGCTG-GTAGAAG
PpolycephalumWis1  A-ACGCACCGGGCTCGCCTTTTTTTTGCGGGGGC-GTGACTCGGTAAAAG
Sflavogenita       GGATGCGTCGGGTTGG---TCTCGTAGCGGGCCTCCTCGGCAATT—CCG
```

```
CUR1_4             CGATAGGGATGTTCGAGGGTGACCGAATTGCCGGGCGAGTGGTGAAATAC
HA4_1              CGAAAGGGGTGTTCGAGGGTGACCGAATTGCCGGGC-AGTGGTGAAATAC
CR19_1             CGATAGGGGTGTTCGAGGGTGACCGAATTGCCGGGCGAGTGGTGAAATAC
Pan2               CGATAGGGGTGTTCGAGGGTGACCGAATTGCCGGGCGAGTGGTGAAATAC
CR8_1              CGATAGGGGTGTTCGAGGGTGACCGAATTGCCGGGCGAGTGGTGAAATAC
Fr_K7              CGATAGGGACGTTCGAGGGTGACCGAATTGCTGGGCGAGTGGTGAAATAC
Fr_K15             CGATAGGGACGTTCGAGGGTGACCGAATTGCTGGGCGAGTGGTGAAATAC
CR10               CGATAGGGGTGTTCGAGGGTGACCGAATTGCCGGGCGAGTGGTGAAATAC
It_K61             CAAAAGGGGTGTTCGAGGGTGACCGAATTGCTGGGCGAGTGGTGAAATAC
Fr_K10             CAAAAGGGGTGTTCGAGGGTGACCGAATTGCTGGGCGAGTGGTGAAATAC
It_K66             CAAAAGGGGTGTTCGAGGGTGACCGAATTGCTGGGCGAGTGGTGAAATAC
Uk_K79             CAAAAGGGGTGTTCGAGGGTGACCGAATTGCTGGACGAGTGGTGAAATAC
Pr3_1              CAAAAGGGGTGTTCGAGGGGGACCGAATTTCCGGGCGAGTGGTGAAATAC
It_K71             CGAAAGGGGTGTTCGAGGGTGACCGAATTGCTGGGCGAGTGGTGAAATAC
Fr_K18             CGAAAGGGGTGTTCGAGGGTGACCGAATTGCCGGGCGAGTGGTGAAATAC
Fr_K62             CGAAAGGGGTGTTCGAGGGTGACCGAATTGCTGGGCGAGTGGTGAAATAC
No_K94             CGAAAGGGGTGTTCGAGGGTGACCGAATTGCCGGGCGAGTGGTGAAATAC
Cur_1              CGAAAGGGATGTTCGAGGGTGACCGAATTGCCGGGCGAGTGGTGAAATAC
Az4_1              CGAAAGGGATGTTCGAGGGTGACCGAATTGCCGGGCGAGTGGTGAAATAC
PR_1               CGAAAGGGATGTTCGAGGGTGACCGAATTGCCGGGCGAGTGGTGAAATAC
Butricularis       CGAAAGGGATGTTCGAGGGTGACCGAATTGCCGGGCGAGTGGTGAAATAC
NY_1               GAAATAGGATGTTCGAGGGTGACCGAATTGCTGGGCGAGTGGTGAAATAC
Poblonga           CGAAGAGGATGTTCGAGGGTGACCGAATTGCCGGGCGAGCGGTGAAATGC
Idn2               CGAAAGGGATGTTCGAGGGTGACCGAATTGCAGGGCGAGTGGTGAAATAC
CJ1_1              CGAAAGG-ATGTTCGAGGGTGACCGAATTGCTTGGCGAGTGGTGAAATAC
CR_1               CGAAAGGGATGTTCGAGGGTGACCGAACTGCTTGGCGAGTGGTGAAATAC
Pdidermoides       CGAAAGGGATGTTCGAGGGTGACCGAATTGCCGGGCGAGTGGTGAAATAC
UFF1               CGAAAGGGATGTTCGAGGGTGACCGAATTGCTGGGCGAGTGGTGAAATAC
CJA3               CGAAAGGGATGTTCGAGGGTGACCGAATTGCCGGGCGAGTGGTGAAATAC
Prigidum           CAATAGGGATGTTCGAGGGTGACCGAATTGCTGGGCGAGTGGTGAAATAC
C1                 CGAAAGGGATGTTCGAGGGTGACCGAATTGCTGGGCGAGTGGTGAAATAC
PpolycephalumWis1  CGAAAGGGATGTTCGAGGGTGACCGAATTGCTGGGCGAGTGGTGAAATAC
Sflavogenita       CGAAAGGGGTGTTCGGGGGCAGCCGAATTGCCGGGCTAGTGGTGAAATAC

CUR1_4             GTTGACCCTGGCAAGTCGACCAAAGGCGAAAGCAGTCATCAAGGGCATAC
HA4_1              GTTGACCCTGGCAAGTCGACCAAAGGCGAAAGCAGT-ATCAAGGGCACGC
CR19_1             GTTGACCCTGGCAAGTCGACCAAAGGCGAAAGCAGTTATCAAGGGCACAC
Pan2               GTTGACCCTGGCAAGTCGACCAAAGGCGAAAGCAGTCATCAAGGGCACAC
CR8_1              GTTGACCCTGGCAAGTCGACCAAAGGCGAAAGCAGTCATCAAGGGCACAC
Fr_K7              GTTGACCCTAGCAAGTCGACCAAAGGCGAAAGCAGTCATCAAGGGCATAC
Fr_K15             GTTGACCCTAGCAAGTCGACCAAAGGCGAAAGCAGTCATCAAGGGCATAC
CR10               GTTGACCCTGGCAAGTCGACCAAAGGCGAAAGCAGTCATCAAGGGCACGC
It_K61             GTTGACCCTAGCAAGTCGACCAAAGGCGAAAGCAGTCATCAAGGGCATTC
Fr_K10             GTTGACCCTAGCAAGTCGACCAAAGGCGAAAGCAGTCATCAAGGGCATTC
It_K66             GTTGACCCTGGCAAGTC-ACCAAAGGCGAAAGCAGTCATCAAGGGCATTC
Uk_K79             GTTGACCCTAGCAAGTCGACCAAAGGCGAAAGCAGTCATCAAGGGCATTC
Pr3_1              GTTGACCCTGGAAAGTCGACCAAAGGCGAAAGCAGTCCTCAAGGGCATTC
It_K71             GTTGACCCTAGCAAGTCGACCAAAGGCGAAAGCAGTCATCAAGGGCACAC
Fr_K18             GTTGACCCTGGCAAGTCGACCAAAGGCGAAAGCAGTCATCAAGGGCATTC
Fr_K62             GTTGACCCTAGCAAGTCGACCAAAGGCGAAAGCGGTCATCAAGGGCACAC
No_K94             GTTGACCCTGGCAAGTCGACCCAAGGCGAAAGCAGTCATCAAGGGCACAC
Cur_1              GTTGACCCTGGCAAGTCGACCAAAGGCGAAAGCAGTCATCAAGGGCATAC
Az4_1              GTTGACCCTGGCAAGTCGACCAAAGGCGAAAGCAGTCATCAAGGGCATAC
PR_1               GTTGACCCTGGCAAGTCGACCAAAGGCGAAAGCAGTCATCAAGGGCATAC
Butricularis       GTTGACCCTGGCAAGTCGACCAAAGGCGAAAGCAGTCATCAAGGGCATAC
NY_1               GTTGACCCTAGCAAGTCGACCAAAGGCGAAAGCAGTCATCAAGGGCATAC
Poblonga           GTTGACCCTGGCAAGTCGACCAAAGGCGAAAGCAGTCATCAAGGGCATAC
Idn2               GTTGACCCTTGCAAGTCGACCAAAGGCGAAAGCAGTCATCAAGGGCATGC
CJ1_1              GGTGACCCTAGCAAGTCGACCAAAGGCGAAAGCAGTCATCAAGGGCATGC
CR_1               GGTGACCCTA-CAAGTCGACCAAAGGCGAAAGCAGTCATCAAGGGCATGC
Pdidermoides       GTTGACCCTGGCAAGTCGACCAAAGGCGAAAGCAGTCATCAAGGGCATGC
UFF1               GGTGACCCTAGCAAGTCGACCAAAGGCGAAAGCAGTCATCAAGGGCATAC
CJA3               GTTGACCCTGGCAAGTCGACCAAAGGCGAAAGCAGTCATCAAGGGCATAC
Prigidum           GTTGACCCTAGCAAGTCGACCAATGGCGAAAGCAGTCATCAAGGGCATAC
C1                 GTTGACCCTAGCAAGTCGACCAAAGGCGAAAGCAGTCATCAAGGGCATAC
PpolycephalumWis1  GTTGACCCTAGCAAGTCGACCAAAGGCGTAAGCAGTCATCAAGGGCATTC
Sflavogenita       GTTGACCCTGGCAAGTCGACCGAAA-CGAAAGCAGCTGTCAAGGGCACAC
```

```
CUR1_4             CCGTTGATCAAGAGCGAAAGTTAAGGGTTCGAAGACGATCAGATACCGTC
HA4_1              CCGTTGATCAAGAGCGAAAGTTAAGGGTTCGAAGAC-ATCAGATACCGTC
CR19_1             CCGTTGATCAAGAGCGAAAGTTAAGGGTTCGAAGACGATCAGATACCGTC
Pan2               CCGTTGATCAAGAGCGAAAGTTAAGGGTTCGAAGACGATCAGATACCGTC
CR8_1              CCGTTGATCAAGAGCGAAAGTTAAGGGTTCGAAGACGATCAGATACCGTC
Fr_K7              CCGTTGATCAAGAGCGAAAGTTAAGGGTTCGAAGACGATCAGATACCGTC
Fr_K15             CCGTTGATCAAGAGCGAAAGTTAAGGGTTCGAAGACGATCAGATACCGTC
CR10               CCGTTGATCAAGAGCGAAAGTTAAGGGTTCGAAGACGATCAGATACCGTC
It_K61             CCGTTGATCAAGAGCGAAAGTTAAGGGTTCGAAGACGATCAGATACCGTC
Fr_K10             CCGTTGATCAAGAGCGAAAGTTAAGGGTTCGAAGACGATCAGATACCGTC
It_K66             CCGTTGATCAAGAGCGAAAGTTAAGGGTTCGAAGACGATCAGATACCGTC
Uk_K79             CCGTTGATCAAGAGCGAAAGTTAAGGGTTCGAAGACGATCAGATACCGTC
Pr3_1              CCGTTGATCAAGAGCGAAAGTTAAGGGTTCGAAGACGATCAGATACCGTC
It_K71             CCGTTGATCAAGAGCGAAAGTTAAGGGTTCGAAGACGATCAGATACCGTC
Fr_K18             CCGTTGATCAAGAGCGAAAGTTAAGGGTTCGAAGACGATCAGATACCGTC
Fr_K62             CCGTTGATCAAGAGCGAAAGTTAAGGGTTCGAAGACGATCAGATACCGTC
No_K94             CCGTTGATCAAGAGCGAAAGTTAAGGGTTCGAAGACGATCAGATACCGTC
Cur_1              CCGTTGATCAAGAGCGAAAGTTAAGGGTTCGAAGACGATCAGATACCGTC
Az4_1              CCGTTGATCAAGAGCGAAAGTTAAGGGTTCGAAGACGATCAGATACCGTC
PR_1               CCGTTGATCAAGAGCGAAAGTTAAGGGTTCGAAGACGATCAGATACCGTC
Butricularis       CCGTTGATCAAGAGCGAAAGTTAAGGGTTCGAAGACGATCAGATACCGTC
NY_1               CCGTTGATCAAGAGCGAAAGTTAAGGGTTCGAAGACGATCAGATACCGTC
Poblonga           CTGTCGATCAAGAGCGAAAGTCAAGGGCTCGAAGACGATCAGATACCGTC
Idn2               CCGTTGATCAAGAGCGAAAGTTAAGGGTTCGAAGACGATCAGATACCGTC
CJ1_1              CCGTTGATCAAGAGCGAAAGTTAAGGGTTCGAAGACGATCAGATACCGTC
CR_1               CCGTTGATCAAGAGCGAAAGTTAAGGGTTCGAAGACGATCAGATACCGTC
Pdidermoides       CCGTTGATCAAGAGCGAAAGTTAAGGGTTCGAAGACGATCAGATACCGTC
UFF1               CCGTTGATCAAGAGCGAAAGTTAAGGGTTCGAAGACGATCAGATACCGTC
CJA3               CCGTTGATCAAGAGCGAAAGTTAAGGGTTCGAAGACGATCAGATACCGTC
Prigidum           CCGTTGATCAAGAGCGAAAGTTAAGGGTTCGAAGACGATCAGATACCGTC
C1                 CCGTTGATCAAGAGCGAAAGTTAAGGGTTCGAAGACGATCAGATACCGTC
PpolycephalumWis1  CCGTTGATCAAGAGCGAAAGTTAAGGGTTCGAAGACGATCAGATACCGTC
Sflavogenita       CCGTTGATCAAGAGCGAAAGTTAAGGGTTCGAAGACGATCAGATACCGTC

CUR1_4             GTAGTCTTAACTATAAATGATACTGACCAGGGATAGGACTCTGTCCATCT
HA4_1              GTAGTCTTAACTATAAATGATACTGACCAGGGATAGGACTCTGTCCATCT
CR19_1             GTAGTCTTAACTATAAATGATACTGACCAGGGATTGGACCCTGTCCATCT
Pan2               GTAGTCTTAACTATAAATGATACTGACCAGGGAT-AGACTCTGTCCATCT
CR8_1              GTAGTCTTAACTATAAATGATACTGACCAGGGAT-AGACTCTGTCCATCT
Fr_K7              GTAGTCTTAACTATAAATGATACTGACTAGGGATAGGACACCGTCCATCT
Fr_K15             GTAGTCTTAACTATAAATGATACTGACTAGGGATAGGACACCGTCCATCT
CR10               GTAGTCTTAACTATAAATGATACTGACCAGGGATAGGACTCTGTCCATCT
It_K61             GTAGTCTTAACTATAAATGATACTGACCAGAGATAGGATTCTGTCCATCT
Fr_K10             GTAGTCTTAACTATAAATGATACTGACCAGAGATAGGATTCTGTCCATCT
It_K66             GTAGTCTTAACTATAAATGATACTGACCAGAGATAGGATTCTGTCCATCT
Uk_K79             GTAGTCTTAACTATAAATGATACTGACCAGAGATAGGATTCTGTCCATCT
Pr3_1              GTAGTCTTAACTATAAATGATACTGACCAGAGATAGGATTCTGTCCATCT
It_K71             GTAGTCTTAACTATAAATGATACTGACCAGGGATAGGATACTGTCCATCT
Fr_K18             GTAGTCTTAACTATAAATGATACTGACCAGGGATAGGATACTGTCCATCT
Fr_K62             GTAGTCTTAACTATAAATGATACTGACCAGGGATAGGATACTGTCCATCT
No_K94             GTAGTCTTAACTATAAATGATACTGACCAGGGATAGGATACTGTCCATCT
Cur_1              GTAGTCTTAACTATAAATGATGCAAACCAGGGATAGGACACTGTCCATCT
Az4_1              GTAGTCTTAACTATAAATGATGCAAACCAGGGATAGGACACTGTCCATCT
PR_1               GTAGTCTTAACTATAAATGATGCAAACCAGGGATAGGACACTGTCCATCT
Butricularis       GTAGTCTTAACTATAAATGATGCAAACCAGGGATAGGACAGTGTCCATCT
NY_1               GTAGTCTTAACTATAAATGATACAGACTAGGGATAGGACACTGTCCATCT
Poblonga           GTAGTCTTGACTATAAACGATACAGACCAGGGATAGGACTCTGTCCATCC
Idn2               GTAGTCT-AACTATAAATGATACAAACCAGGGATGAGACACTGTCCATTC
CJ1_1              GTAGTCTTAACTATAAATGATACAAACCAGGGATGAGACACTGTCCATCT
CR_1               GTAGTCTTAACTATAAATGATACAAACCAGGGATGAGACACTGTCCATCT
Pdidermoides       GTAGTCTTAACTATAAATGATACAAACCAGGGATGAGACACTGTCCATCT
UFF1               GTAGTCTTAACTATAAACGATACAGACCAGGGATAGGACAGTGTCCATCT
CJA3               GTAGTCTTAACTATAAATGATGCAAACCAGGGATAGGACACTGTCCATCT
Prigidum           GTAGTCTTAACTATAAATGATGCAGACCAGGGATAGGACAGTGTCCATCT
C1                 GTAGTCTTAACTATAAATGATGCAAACCAGAGATAGGACCCTGTCCATCT
PpolycephalumWis1  GTAGTCTTAACTATAAATGATGCAGACCAGGGATAGGACAGTGTCCATCT
Sflavogenita       GTAGTCTTAACTATAAATGATACTGGCCAGGGATAGGGCAATGTCCATCT
```

```
CUR1_4             CGACTCCTTCTGGACCTTGGAGAAATTA-CAGTCTTTGAGTTCTGGGGGG
HA4_1              CGACTCCTCCTGAACCTTGGAGAAATTA-CAGTCTTTGAGTTCTGGGGGG
CR19_1             CGACTCCTTCAAGACCTTGGAGAAATTA-CAGTCTTTGAGTTCTGGGGGG
Pan2               CGACTTCTTCTGGACCTTGGAGAAATTA-CAGTCTTTGAGTTCTGGGGGG
CR8_1              CGACTTCTTCTGGACCTTGGAGAAATTA-CAGTCTTTGAGTTCTGGGGGG
Fr_K7              CGACTCCTCCTGGACCTTAGAGAAATCA-AAGTCTTTGAGTTCTGGGGGG
Fr_K15             CGACTCCTCCTGAACCTTAGAGAAATCA-AAGTCTTTGAGTTCTGGGGGG
CR10               CGACTTCTCCTGGACCTTAGAGAAATTA-CAGTCTTTGAGTTCTGGGGGG
It_K61             CGACTTCTCCTGGATCTTTGAGAAATTA-TAGTCTTTGAGTTCTGGGGGG
Fr_K10             CGACTTCTCCTGGATCTTAGGGAAACTA-TAGTCTTTGAGTTCTGGGGGG
It_K66             CGACTTCTCCTGGATCTTAGGGAAACTA-TAGTCTTTGAGTTCTGGGGGG
Uk_K79             CGACTTCTCCTGGATCTTAGGGAAACTA-TAGTCTTTGAGTTCTGGGGGG
Pr3_1              CGACTTCTCCTGGATCTTAGGGAAACTA-GAGTCTTTGAGTTCTGGGGGG
It_K71             CGACTCTTCCCGAACCTTAGAGAAATCA-GAGTCTTTGAGTTCTGGGGGG
Fr_K18             CGACTCTTCCCGGACCTTAGAGAAATCA-GAGTCTTTGAGTTCTGGGGGG
Fr_K62             CGACTCTTCCCGAACCTTAGAGAAATCA-GAGTCTTTGAGTTCTGGGGGG
No_K94             CGACTCTTCCCGGACCTTAGAGAAATCA-CAGTCTTTGAGTTCTGGGGGG
Cur_1              CGACTCCTCCTGGACCTTAGAGAAATTA-CAGTCTTTGGGTTCTGGGGGG
Az4_1              CGACTCCTCCTGGACCTTATAGAAATCATCAGTCTTTGGGTTCTGGGGGG
PR_1               CGACTCCTCCTGGACCTTAGAGAAATTA-CAGTCTTTGGGTTCTGGGGGG
Butricularis       CGACTCCTCCCGAACCTTGGAGAAATTAC-AGTCTATGGGTTCTGGGGGG
NY_1               CGACTCCTCCTGAATCTTTGAGAAATTACCAGTCTTTGGGTTCTGGGGGG
Poblonga           CGACTTCTCCTGAATCTTGAAGAAATTACCAGTCTTTGGGTTCCGGGGGG
Idn2               CGACTCCTCTCGAACCTTAGAGAAATCA-GAGTCTTTGGGTTCTGGGGGG
CJ1_1              CGACTCCTTTCGAACCTTAGAGAAATCA-GAGTCTTTGGGTTCTGGGGGG
CR_1               CGACTCCTTTCGAACCTTAGAGAAATCA-GAGTCTTTGGGTTCTGGGGGG
Pdidermoides       CGACTCCTTTCGAACCTTAGAGAAATCA-GAGTCTTTGGGTTCTGGGGGG
UFF1               CGACTCCTCCCGGACCTTGAAGAAATCATGAGTCTTTGGGTTCTGGGGGG
CJA3               CGACTCCTCCTGGACCTTAGAGAAATTA-CAGTCTTTGGGTTCTGGGGGG
Prigidum           CGACTCCTCCTGGACCTCGGAGAAATT-CGAGTCTTTGGGTTCTGGGGGG
C1                 CGACTCCTTCTGGATCCTGGAGAAATTA-GAGTCTTTGGGTTCTGGGGGG
PpolycephalumWis1  CGACTCTTCC-GGACCTTGGAGAAATCACGAGTCTATGGGTTCTGGGGGG
Sflavogenita       CGACTCCCCCCGGACTTTTGGGAAACCA-AAGCCTATGAGTTCTGGGGGG

CUR1_4             AGTATGGTCGCAAGGCTGAAACTTAAAGGAATTGACGGAAGGGCACACAA
HA4_1              AGTATGGTCGCAAGGCTGAAACTTAAAGGAATTGACGGAAGGGCACACAA
CR19_1             AGTATGGTCGCAAGGCTGAAACTTAAAGGAATTGACGGAAGGGCACACAA
Pan2               AGTATGGTCGCAAGGCTGAAACTTAAAGGAATTGACGGAAGGGCACACAA
CR8_1              AGTATGGTCGCAAGGCTGAAACTTAAAGGAATT-----------------
Fr_K7              AGTATGGTCGCAAGGCTGAAACTTAAAGGAATTGACGGAAGGGCACACAA
Fr_K15             AGTATGGTCGCAAGGCTGAAACTTAAAGGAATTGACGGAAGGGCACACAA
CR10               AGTATGGTCGCAAGGCTGAAACTTAAAGGAATTGACGGAAGGGCACACAA
It_K61             AGTATGGTCGCAAGGCTGAAACTTAAAGGAATTGACGGAAGGGCACACAA
Fr_K10             AGTATGGTCGCAAGGCTGAAACTTAAAGGAATTGACGGAAGGGCACACAA
It_K66             AGTATGGTCGCAAGGCTGAAACTTAAAGGAATTGACGGAAGGGCACACAA
Uk_K79             AGTATGGTCGCAAGGCTGAAACTTAAAGGAATTGACGGAAGGGCACACAA
Pr3_1              AGTATGGTCGCAAGGCTGAAACTTAAAGGAATTGACGGAAGGGCACACAA
It_K71             AGTATGGTCGCAAGGCTGAAACTTAAAGGAATTGACGGAAGGGCACACAA
Fr_K18             AGTATGGTCGCAAGGCTGAAACTTAAAGGAATTGACGGAAGGGCACACAA
Fr_K62             AGTATGGTCGCAAGGCTGAAACTTAAAGGAATTGACGGAAGGGCACACAA
No_K94             AGTATGGTCGCAAGGCTGAAACTTAAAGGAATTGACGGAAGGGCACACAA
Cur_1              AGTATGGTCGCAAGGCTGAAACTTAAAGGAATTGACGGAAGGGCACACAA
Az4_1              AGTATGGTCGCAAGGCTGAAACTTAAAGGAATTGACGGAAGGGCACACAA
PR_1               AGTATGGTCGCAAGGCTGAAACTTAAAGGAATTGACGGAAGGGCACACAA
Butricularis       AGTATGGTCGCAAGGCTGAAACTTAAAGGAATTGACGGAAGGGCACACAA
NY_1               AGTATGGTCGCAAGGCTGAAACTTAAAGGAATTGACGGAAGGGCACACAA
Poblonga           AGTATGGTCGCAAGGCTGAAACTTAAAGGAATTGACGGAAGGGCACACAA
Idn2               AGTATGGTCGCAAGGCTGAAACTTAAAGGAATTGACGGAAGGGCACACAA
CJ1_1              AGTATGGTCGCAAGGCTGAAACTTAAAGGAATTGACGGAAGGGCACACAA
CR_1               AGTATGGTCGCAAGGCTGAAACTTAAAGGAATTGACGGAAGGGCACACAA
Pdidermoides       AGTATGGTCGCAAGGCTGAAACTTAAAGGAATTGACGGAAGGGCACACAA
UFF1               AGTATGGTCGCAAGGCTGAAACTTAAAGGAATTGACGGAAGGGCACACAA
CJA3               AGTATGGTCGCAAGGCTGAAACTTAAAGGAATTGACGGAAGGGCACACAA
Prigidum           AGTATGGTCGCAAGGCTGAAACTTAAAGGAATTGACGGAAGGGCACACAA
C1                 AGTATGGTCGCAAGGCTGAAACTTAAAGGAATTGACGGAAGGGCACACAA
PpolycephalumWis1  AGTATGGTCGCAAGGCTGAAACTTAAAGGAATTGACGGAAGGGCACACAA
Sflavogenita       AGTATGGTCGCAAGGCTGAAACTTAAAGGAATTGACGGAAGGGCACACAA
```

```
CUR1_4             AGAGTGGAACCTGCGGCTTAATTTGACTCAACACGGGAAAACTCACCAGG
HA4_1              AGAGTGGAACCTGCGGCTTAATTTGACTCAACACGGGAAAACTCACCAGG
CR19_1             AGAGTGGAACCTGCGGCTTAATTTGACTCAACACGGGAAAACTCACCAGG
Pan2               AGAGTGGAACCTGCGGCTTAATTTGACTCAACACGGGAAAACTCACCAGG
CR8_1              -------------------AATTTGACTCAACACGGGAAAACTCACCAGG
Fr_K7              AGAGTGGAACCTGCGGCTTAATTTGACTCAACACGGGAAAACTCACCAGG
Fr_K15             AGAGTGGAACCTGCGGCTTAATTTGACTCAACACGGGAAAACTCACCAGG
CR10               AGAGTGGAACCTGCGGCTTAATTTGACTCAACACGGGAAAACTCACCAGG
It_K61             AGAGTGGAACCTGCGGCTTAATTTGACTCAACACGGGAAAACTCACCAGG
Fr_K10             AGAGTGGAACCTGCGGCTTAATTTGACTCAACACGGGAAAACTCACCAGG
It_K66             AGAGTGGAACCTGCGGCTTAATTTGACTCAACACGGGAAAACTCACCAGG
Uk_K79             AGAGTGGAACCTGCGGCTTAATTTGACTCAACACGGGAAAACTCACCAGG
Pr3_1              AGAGTGGAACCTGCGGCTTAATTTGACTCAACACGGGAAAACTCACCAGG
It_K71             AGAGTGGAACCTGCGGCTTAATTTGACTCAACACGGGAAAACTCACCAGG
Fr_K18             AGAGTGGAACCTGCGGCTTAATTTGACTCAACACGGGAAAACTCACCAGG
Fr_K62             AGAGTGGAACCTGCGGCTTAATTTGACTCAACACGGGAAAACTCACCAGG
No_K94             AGAGTGGAACCTGCGGCTTAATTTGACTCAACACGGGAAAACTCACCAGG
Cur_1              AGAGTGGAACCTGCGGCTTAATTTGACTCAACACGGGAAAACTCACCAGG
Az4_1              AGAGTGGAACCTGCGGCTTAATTTGACTCAACACGGGAAAACTCACCAGG
PR_1               AGAGTGGAACCT-CGGCTTAATTTGACTCAACACGGGAAAACTCACCAGG
Butricularis       AGAGTGGAACCTGCGGCTTAATTTGACTCAACACGGGAAAACTCACCAGG
NY_1               AGAGTGGAACCTGCGGCTTAATTTGACTCAACACGGGAAAACTCACCAGG
Poblonga           AGAGTGGAACCTGCGGCTTAATTTGACTCAACACGGGAAAACTCACCAGG
Idn2               AGAGTGGAACCTGCGGCTTAATTTGACTCAACACGGGAAAACTCACCAGG
CJ1_1              AGAGTGGAACCTGCGGCTTAATTTGACTCAACACGGGAAAACTCACCAGG
CR_1               AGAGTGGAACCTGCGGCTTAATTTGACTCAACACGGGAAAACTCACCAGG
Pdidermoides       AGAGTGGAACCTGCGGCTTAATTTGACTCAACACGGGAAAACTCACCAGG
UFF1               AGAGTGGAACCTGCGGCTTAATTTGACTCAACACGGGAAAACTCACCAGG
CJA3               AGAGTGGAACCTGCGGCTTAATTTGACTCAACACGGGAAAACTCACCAGG
Prigidum           AGAGTGGAACCTGCGGCTTAATTTGACTCAACACGGGAAAACTCACCAGG
C1                 AGAGTGGAACCTGCGGCTTAATTTGACTCAACACGGGAAAACTCACCAGG
PpolycephalumWis1  AGAGTGGAACCTGCGGCTTAATTTGACTCAACACGGGAAAACTCACCAGG
Sflavogenita       AGAGTGGAACCTGCGGCTTAATTTGACTCAACACGGGAAAACTCACCGGG

CUR1_4             TCCAGATACACGTATGAAAGACAAGCTGAAAGACTTTTCTCAATGATGTA
HA4_1              TCCAGATACACGTATGAAAGACAAGCTGAAAGACTTTTCTCAATGATGTA
CR19_1             TCCAGATACACGTATGAAAGACAAGCTGAAAGACTTTTCTCAATGATGTA
Pan2               TCCAGATACACGTATGAAAGACAAGCTGAAAGACTTTTCTCAATGATGTA
CR8_1              TCCAGATACACGTATGAAAGACAAGCTGAAAGACTTTTCTCAATGATGTA
Fr_K7              TCCGGATACACGTATGAAAGACAAGCTGAAAGACTTTTCTCAATGATGTA
Fr_K15             TCCGGATACACGTATGAAAGACAAGCTGAAAGACTTTTCTCAATGATGTA
CR10               TCCAGATACACGTATGAAAGACAAGCTGAAAGACTTTTCTCAATGATGTA
It_K61             TCCGGATACACGTATGAAAGACAAGCTGAAAGACTTTTCTCAATGATGTA
Fr_K10             TCCAGATACACGTATGAAAGACAAGCTGAAAGACTTTTCTCAATGATGTA
It_K66             TCCAGATACACGTATGAAAGACAAGCTGAAAGACTTTTCTCAATGATGTA
Uk_K79             TCCAGATACACGTATGAAAGACAAGCTGAAAGACTTTTCTCAATGATGTA
Pr3_1              TCCAGATACACGTATGAAAGACAAGCTGAAAGACTTTTCTCAATGATGTA
It_K71             TCCGGATACACGTATGAAAGACAAGCTGAAAGACTTTTCTCAATGATGTA
Fr_K18             TCCGGATACACGTATGAAAGACAAGCTGAAAGACTTTTCTCAATGATGTA
Fr_K62             TCCGGATACACGTATGAAAGACAAGCTGAAAGACTTTTCTCAATGATGTA
No_K94             TCCAGATACACGGATGAAAGACAAGCTGACAGACTTTTCTCAATGATGTA
Cur_1              TCCGGATACACGTATGAAAGTCAAGCTGAAAGACTTTACTCAATGATGTA
Az4_1              TCCGGATACACGTATGAAAGTCAAGCTGAAAGACTTTACTCAATGATGTA
PR_1               TCCGGATACACGTATGAAAGTCAAGCTGAAAGCCTTTACTCAATGATGTA
Butricularis       TCCGGATACACGTATGAAAGTCAAGCTGAAAGACTTTACTCAATGATGTA
NY_1               TCCGGATACACGTATGAAAGTCAAGCTGAAAGACTTTACTCAATGATGTA
Poblonga           TCCAGATACACGTATGAAAGTCAAGCTGAAAGACTTTACTCAATGATGTA
Idn2               TCCGGATACACGTATGAAAGTCAAGCTGAAAGACTTTACTCAATGATGTA
CJ1_1              TCCGGATACACGTATGAAAGTCAAGCTGAAAGACTTTACTCAATGATGTA
CR_1               TCCGGATACACGTATGAAAGTCAAGCTGAAAGACTTTACTCAATGATGTA
Pdidermoides       TCCGGATACACGTATGAAAGTCAAGCTGAAAGACTTTACTCAATGATGTA
UFF1               TCCAGATACACGTATGAAAGTCAAGCTGAAAGACTTTACTCAATGATGTA
CJA3               TCCGGATACACGTATGAAAGTCAAGCTGAAAGACTTTACTCAATGATGTA
Prigidum           TCCGGATACACGTATGAAGGTCAAGCTGAATGACTTTACTCAATGATGTA
C1                 TCCAGATACACGCATGAAAGTCAAGCTGAAAGACTTTACTCAATGATGTA
PpolycephalumWis1  TCCGGATACACGTATGAAAGTCAAGCTGAAAGACTTTACTCAATGATGTA
Sflavogenita       TCCAGATACATGGATGAAAGACAAGCTGATAGACTTTTCTCAATTATGTA
```

```
CUR1_4              AGTGGTGGTGCATGGTCGTT-CTTAGTTCGTGGATTGATTTGTCTGGTCT
HA4_1               AGTGGTGGTGCATGGTCGTT-CTTAGTTCGTGGATTGATTTGTCTGGTCT
CR19_1              AGTGGTGGTGCATGGTCGTT-CTTAGTTCGTGGATTGATTTGTCTGGTCT
Pan2                AGTGGTGGTGCATGGTCGTT-CTTAGTTCGTGGATTGATTTGTCTGGTCT
CR8_1               AGTGGTGGTGCATGGTCGTT-CTTAGTTCGTGGATTGATTTGTCTGGTCT
Fr_K7               AGTGGTGGTGCATGGTCGTT-CTTAGTTCGTGGAGTGGTTTGTCTGGTTT
Fr_K15              AGTGGTGGTGCATGGTCGTT-CTTAGTTCGTGGAGTGGTTTGTCTGGTTT
CR10                AGTGGTGGTGCATGGTCGTT-CTTAGTTCGTGGATTGATTTGTCTGGTCT
It_K61              AGTGGTGGTGCATGGTCGTT-CTTAGTTCGTGGATTGATTTGTCTGGTCT
Fr_K10              AGTGGTGGTGCATGGTCGTT-CTTAGTTCGTGGATTGATTTGTCTGGTCT
It_K66              AGTGGTGGTGCATGGTCGTTTCTTAGTTCGTGGATTGATTTGTCTGGTCT
Uk_K79              AGTGGTGGTGCATGGTCGTT-CTTAGTTCGTGGATTGATTTGTCTGGTCT
Pr3_1               AGTGGTGGTGCATGGTCNTT-CGTAGTTCGTGGATTGATTTGTCTGGTCT
It_K71              AGTGGTGGTGCATGGTCGTT-CTTAGTTCGTGGAGTGGTTTGTCTGGTCT
Fr_K18              AGTGGTGGTGCATGGTCGTT-CTTAGTTCGTGGAGTGGTTTGTCTGGTCT
Fr_K62              AGTGGTGGTGCATGGTCGTT-CTTAGTTCGTGGAGTGGTTTGTCTGGTCT
No_K94              AGTGGTGGTGCATGGTCGTT-CTTAGTTCGTGGAGTGATTTGTCTGGTCT
Cur_1               AGTGGTGGTGCATGGTCGTTCT-TAGTTCGTGGATTGATTTGTCTGGTCT
Az4_1               AGTGGTGGTGCATGGTCGTTCT-TAGTTCGTGGATTGATTTGTCTGGTCT
PR_1                AGTGGTGGTGCATGGTCGTTCT-TAGTTCGTGGATTGATTTGTCTGGTCT
Butricularis        AGTGGTGGTGCATGGTCGTTCT-TAGTTCGTGGATTGATTTGTCTGGTCT
NY_1                AGTGGTGGTGCATGGTCGTTCT-TAGTTCGTGGATTGATTTGTCTGGTCT
Poblonga            AGTGGTGGTGCATGGTCGTTCG-TAGTTCGTGGATTGATTTGTCTGGTCT
Idn2                AGTGGTGGTGCATGGTCGTTCG-TAGTTCGTGGATTGATTTGTCTGGTTT
CJ1_1               AGTGGTGGTGCATGGTCGTTCG-TAGTTCGTGGATTGATTTGTCTGGTTT
CR_1                AGTGGTGGTGCATGGTCGTTCGGTAGTTCGTGGATTGATTTGTCTGGTTT
Pdidermoides        AGTGGTGGTGCATGGTCGTTCG-TAGTTCGTGGATTGATTTGTCTGGTTT
UFF1                AGTGGTGGTGCATGGTCGTTCT-TAGTTCGTGGATTGATTTGTCTGGTCT
CJA3                AGTGGTGGTGCATGGTCGTTCT-TAGTTCGTGGATTGATTTGTCTGGTCT
Prigidum            AGTGGTGGTGCATGGTCGTTCT-TAGTTCGTGGATTGATTTGTCTGGTCT
C1                  AGTGGTGGTGCATGGTCGTTCT-TAGTTCGTGGATTGATTTGTCTGGTCT
PpolycephalumWis1   AGTGGTGGTGCATGGTCGTTCT-TAGTTCGTGGATTGATTTGTCTGGTTT
Sflavogenita        AGTGGTGGTGCATGGCCGTT-CTTAGTTCGTGGAGTGGTTTGTCTGGTCT

CUR1_4              ATTCCGATAACGAGCGAGACCCCTACGTTCCTAATAGGGGCGGCAGCCCG
HA4_1               ATTCCGATAACGAGCGAGACCCCGACGTTCCTAATAGGGGTGGCAGCCCG
CR19_1              ATTCCGATAACGAGCGAGACCCCTACGTTCCTACTAGGGGTGGCAGCCCG
Pan2                ATTCCGATAACGAGCGAGACCCCTACGTTCCTAATAGGGGCGGCAGCCCG
CR8_1               ATTCCGATAACGAGCGAGACCCCTACGTTCCTAATAGGGGCGGCAGCCCG
Fr_K7               ATTCCGATAACGAGCGAGACCCCGGCGTTCCTAATAGGGGTGGCAGCCCG
Fr_K15              ATTCCGATAACGAGCGAGACCCCGGCGTTCCTAATAGGGGTGGCAGCCCG
CR10                ATTCCGATAACGAGCGAGACCCCAACGTTCCTAATAGGGGCGGCAGCCCG
It_K61              ATTCCGATAACGAGCGAGACCCCGGCGTTCCTAATAGGGGTGGCAGCCCG
Fr_K10              ATTCCGATAACGAGCGAGACCCCTCCGTTCCTACTAGGGGTGGCAGCCCG
It_K66              ATTCCGATAACGAGCGAGACCCCTCCGTTCCTACTAGGGGTGGCAGCCCG
Uk_K79              ATTCCGATAACGAGCGAGACCCCTCCGTTCCTACTAGGGGTGGCAGCCCG
Pr3_1               ATTCCGATAACGAGCGAGACCCCGGCGTTCCTAATAGGGGCGGCAGCCCG
It_K71              ATTCCGATAACGA-----------------------------------AG
Fr_K18              ATTCCGATAACGAGCGAGACCCCGGCGTTCCTAATAGGGGCAGCAGCCCG
Fr_K62              ATTCCGATAACGAGCGAGACCCCGACGTTCCTAATAGGGGCGGCAGCCCG
No_K94              ATTCCGATAACGAGCGAGACCCCGACGTTCCTAATAGGGGTAGCAGCCCG
Cur_1               ATTCCGATAACGAGCGAGACCCCATCGTTCCTAATAGGGGCGTTAGCCCG
Az4_1               ATTCCGATAACGAGCGAGACCCCATCGTTCCTAATAGGGGCGTTAGCCCG
PR_1                ATTCCGATAACGAGCGAGACCCCATCGTTCCTAATAGGGGCGTTAGCCCG
Butricularis        ATTCCGATAACGAGCGAGACCCCGGCGTTCCTAATAGGGGCGGCAGCCAA
NY_1                ATTCCGATAACGAGCGAGACCCCAGCGTTCCTAGTAGGGCCAACAGCCCG
Poblonga            ATTCCGATAACGAGCGAGACCCCAACGTTCCTAATAGGGGCGACAGCCCG
Idn2                ATTCCGATAACGAGCGAGACCCCAACGTTCCTAATAGGAGCGTTAGCCCG
CJ1_1               ATTCCGATAACGAGCGAGACCCCAACGTTCCTAATAGGGGTGTCGGCCCG
CR_1                ATTCCGATAACGAGCGAGACCCCAACGTTCCTAATAGGGGTGTCGGCCCG
Pdidermoides        ATTCCGATAACGAGCGAGACCCCAACGTTCCTAATAGGGGCTTTAGCCCA
UFF1                ATTCCGATAACGAGCGAGACCCCGACGTTCCTAATAGGGGCGGCAGCCAA
CJA3                ATTCCGATAACGAGCGAGACCCCATCGTTCCTAATAGGGGCGTTAGCCCC
Prigidum            ATTCCGATAACGAGCGAGACCCCAACGTTCCTAATAGGGGTGGCAGCCAA
C1                  ATTCCGATAACGAGCGAGACCCC-ATGTTCCTAATAGGGGCGGTAGCCCG
PpolycephalumWis1   ATTCCGATAACGAGCGAGACCCCGGCGTTCCTAATAGGGGTGGCAGCCAG
Sflavogenita        ATTCCGATAACGAGCGAGACCCC-ACGGTCTAAATAGGGGCCGCAGCCCG
```

```
CUR1_4             GCTTTCCCCCAGCA-GCCGCCTAATTAG----CTTCTTAGACG-TATCAG
HA4_1              GCTCGTCCGCAGCA-GCCGCCTAATTAG----CTTCTTAGACG-TATCAG
CR19_1             GCTTTACCAGAGCA-GCCACCTTTTTAG---CTTCTTAGACG-TATCAG
Pan2               GCTTTGCCCTAGCA-GCCGCCTAATTAA----CTTCTTAGACG-TATCAG
CR8_1              GCTTTGCCCTAGCA-GCCGCCTAATTAA----CTTCTTAGACG-TATCAG
Fr_K7              GCTCGCGCTTAGCACGCCACCTCGCAATTG--CTTCTTAGACG-TATCAG
Fr_K15             GCTCGCGCCTAGCACGCCACCTCGCAATTG--CTTCTTAGACG-TATCAG
CR10               GCTCGCCTGTAGCA-GCCGCCTTATTAG----CTTCTTAGACG-TATCAG
It_K61             GCTCGTCCCTAGCA-GCCACTTAAGATGTA--CTTCTTAGACG-TATCAG
Fr_K10             GCTCGCGCCCAGCA-GCTACTTAAGATTTA--CTTCTTAGACG-TATCAG
It_K66             GCTCGCACCCAGCA-GCTACTTAAGATTTA--CTTCTTAGACG-TATCAG
Uk_K79             GCTCGCGCCCAGCA-GCTACTTAAGATTTA--CTTCTTAGACG-TATCAG
Pr3_1              GCTCGCCGCAAGCA-GCCGCTTAAGATTTA--CTTCTTAGACG-TATCAG
It_K71             GGTGTTCGCTAGCA-GCCGCCTCACAATATG-CTTCTTAGACG-TATCAG
Fr_K18             GCTTTCGCCCAGCACGCTGCCTCACAATATG-CTTCTTAGACG-TATCAG
Fr_K62             GCTTTCGCCCAGCA-GCCGCCTCACAATATG-CTTCTTAGACG-TATCAG
No_K94             GCTCGGTCGCAGCC-GCTACCTCATACAAG--CTTCTTAGACG-TATCAG
Cur_1              CCGTTCGAAAAGCT-TCCGCTTAAGATTAA--CTTCTTAGACG-TATCAG
Az4_1              CCGGTCGCAAAGCT-TCCGCTTAAGATTAA--CTTCTTAGACGGTATCAG
PR_1               CCGTTCGAAAAGCT-TCCGCTTAAGATTAA--CTTCTTAGACG-TATCAG
Butricularis       GCCGGTCGCAAGCTCGCCGCCTGAAGTTAT-ACTTCTTAGACG-TATCAG
NY_1               ACTAGCCGCAAGCTGGCGGCTTAACATAAG--CTTCTTAGACG-TATCAG
Poblonga           GCTTGCCGCAAGC-AGTTGCCTAAGATTCA--CTTCTTAGACG-TATCAG
Idn2               GCGCGTCGCAAGCT-ATCGCTTAA-GATTTG-CTTCTTAGACG-TATCAG
CJ1_1              GCTTGCCTCGAGCC-TTCGCCTTAATGTATG-CTTCTTAGACG-TATCAG
CR_1               GCTTGCCTTGAGCC-TTCGCCTTAATGTATG-CTTCTTAGACG-TATCAG
Pdidermoides       GTGCGTCGCAAGCT-ATCGCTTAAAGATTTG-CTTCTTAGACG-TATCAG
UFF1               GCCGGCCGCAAGCTCGCTGCTTAGAAGTTTTACTTCTTAGACG-TATCAG
CJA3               GCCGTTCGAAAGCT-TCCGCTTAAGATTAA--CTTCTTAGACG-TATCAG
Prigidum           GCCGGCCGCAAGCTCGCCGCCTGAAGTTTTACTTCTTAGACG-TATCAG
C1                 CTGCCGCAAGAGCT-GCTGCTTAAGATTTG--CTTCTTAGACG-TATCAG
PpolycephalumWis1  ACCGGTCGCAAGCTCGCCACCTGAAGTTATG-CTTCTTAGACG-TATCAG
Sflavogenita       GCTCGGCTCGAGCAGCGCGCTTCGAAAGCAAGCTTCTTAGACG-GATCGG

CUR1_4             AGCCGATAAGGTTCTTGAAATGGGTTAATAACAGGTCAGTCATGCCCTTA
HA4_1              AGCCGATAAGGTTCTTGAAATGGGTTAATAACAGGTCAGTCATGCCCTTA
CR19_1             AGCCGATAAGGTTCTTGAAATGGGTTAATAACAGGTCAGTCATGCCCTTA
Pan2               AGCCGATAAGGTTCTTGAAATGGGTTAATAACAGGTCAGTCATGCCCTTA
CR8_1              AGCCGATAAGGTTCTTGAAATGGGTTAATAACAGGTCAGTCATGCCCTTA
Fr_K7              AGCCGATAAGGTTCTTGAAATGGGTTAATAACAGGTCAGTCATGCCCTTA
Fr_K15             AGCCGATAAGGTTCTTGAAATGGGTTAATAACAGGTCAGTCATGCCCTTA
CR10               AGCCGAAAAGGTTCTTGAAATGGGTTAATAACAGGTCAGTCATGCCCTTA
It_K61             AGCCGATAAGGTTCTTGAAATGGGTTAATAACAGGTCAGTCATGCCCTTA
Fr_K10             AGCCGATAAGGTTCTTGAAATGGGTTAATAACAGGTCAGTCATGCCCTTA
It_K66             AGCCGATAAGGTTCTTGAAATGGGTTAATAACAGGTCAGTCATGCCCTTA
Uk_K79             AGCCGATAAGGTTCTTGAAATGGGTTAATAACAGGTCAGTCATGCCCTTA
Pr3_1              AGCCGATAAGGTTCTTGAAATGGGTTAATAACAGGTCAGTCATGCCCTTA
It_K71             AATCGAAAAGGTTCTTGAAATGGGTTAATAACAGGTCAGTCATGCCCTTA
Fr_K18             AGCCGATAAGGTTCTTGAAATGGGTTAATAACAGGTCAGTCATGCCCTTA
Fr_K62             AATCGAAAAGGTTCTTGAAATGGGTTAATAACAGGTCAGTCATGCCCTTA
No_K94             AGCCGATAAGGTTCTTGAAATGGGTTAATAACAGGTCAGTCATGCCCTTA
Cur_1              AGCCGATAAGGTTCTTGAAATGGGTTAATAACAGGTCAGTCATGCCCTTA
Az4_1              AGCCGATAAGGTTCTTGAAATGGGTTAATAACAGGTCAGTCATGCCCTTA
PR_1               AGCCGATAAGGTTCTTGAAATGGGTTAATAACAGGTCAGTCATGCCCTTA
Butricularis       AGCCGACAAGGTTCTTGAAATGGGTTAATAACAGGTCAGTCATGCCCTTA
NY_1               AGCCGATAAGGTTCTTGAAATGGGTTAATAACAGGTCAGTCATGCCCTTA
Poblonga           GGCCGATAAGGTCCTTGAAATGGGTTAATAACAGGTCAGTCATGCCCTTA
Idn2               AGCCGATAAGGCGCTTGAAATGGGTTAATAACAGGTCAGTGATGCCCTTA
CJ1_1              AGCCGATAAGGCGCTTGAAATGGGTTAATAACAGGTCAGTGATGCCCTTA
CR_1               AGCCGATAAGGCGCTTGAAATGGGTTAATAACAGGTCAGTGATGCCCTTA
Pdidermoides       AGCCGATAAGGCGCTTGAAATGGGTTAATAACAGGTCAGTGATGCCCTTA
UFF1               GGCCGATAAGGTCCTTGAAATGGGTTAATAACAGGTCAGTCATGCCCTTA
CJA3               AGCCGATAAGGTTCTTGAAATGGGTTAATAACAGGTCAGTCATGCCCTTA
Prigidum           AGCCGATAAGGTTCTTGAAATGGGTTAATAACAGGTCAGTCATGCCCTTA
C1                 AGCCGATAAGGTTCTTGAAATGGGTTAATAACAGGTCAGTCATGCCCTTA
PpolycephalumWis1  AGCCGATAAGGTTCTTGAAATGGGTTAATAACAGGTCAGTCATGCCCTTA
Sflavogenita       GGCCGATAAGGTCTCTGAAATGGGTTAATAACAGGTCAGTCATGCCCTTA
```

```
CUR1_4              GATGTTCTGGGCCGCACGCGCGTTACAATGGTATACATTAGAACGTGCTC
HA4_1               GATGTTCTGGGCCGCACGCGCGTTACAATGGTATACATTAGAACGTGCTC
CR19_1              GATGTTCTGGGCCGCACGCGCGTTACAATGGTATACATTAGAACGTGCTC
Pan2                GATGTTCTGGGCCGCACGCGCGTTACAATGGTATACAGTAGAACGTGCCC
CR8_1               GATGTTCTGGGCCGCACGCGCGTTACAATGGTATACAGTAGAACGTGCCC
Fr_K7               GATGTTCTGGGCCGCACGCGCGTTACAATGGTAGGC-TTAAACAGTGCCT
Fr_K15              GATGTTCTGGGCCGCACGCGCGTTACAATGGTAGGC-TTAAACAGTGCCT
CR10                GATGTTCTGGGCCGCACGCGCGTTACAATGGTATACATTAGAACGTGCTC
It_K61              GATGTTCTGGGCCGCACGCGCGTTACAATGGCATGT-ATACATAGTGCCT
Fr_K10              GATGTTCTGGGCCGCACGCGCGTTACAATGGCATGA-ATAAAAAGTGCCT
It_K66              GATGTTCTGGGCCGCACGCGCGTTACAATGGCATGA-ATAAAAAGTGCCT
Uk_K79              GATGTTCTGGGCCGCACGCGCGTTACAATGGTATGA-ATAAAAAGTGCCT
Pr3_1               GATGTTCTGGGCCGCACGCGCGTTACAATGGCACGT-ATAGAAAGTGCCT
It_K71              GATGTTCTGGGCCGCACGCGCGTTACAATGGCATGCTTTAGAAAGTGCTC
Fr_K18              GATGTTCTGGGCCGCACGCGCGTTACAATGGCATGCTTTAGAAAGTGCTC
Fr_K62              GATGTTCTGGGCCGCACGCGCGTTACAATGGCATGCTTTAGAAAGTGCTC
No_K94              GATGTTCTGGGCCGCACGCGCGTTACAATGGCATG-TAAAGAAAGTGCCT
Cur_1               GATGTTCTGGGCCGCACGCGCGTTACAATGGCATGT-AAAGCGAGTGCTT
Az4_1               GATGTTCTGGGCCGCACGCGCGTTACAATGGCACGT-AAAGCGAGTGCTT
PR_1                GATGTTCTGGGCCGCACGCGCGTTACAATGGCATGT-AAAGCGAGTGCTT
Butricularis        GATGTTCTGGGCCGCACGCGCGTTACAATGGCACGT-ATACCGAGTGCTT
NY_1                GATGTTCTGGGCCGCACGCGCGTTACAATGGCATGT-AAAGCGAGTGCCG
Poblonga            GATGTTCTGGGCCGCACGCGCGTTACAATGGCACGT-T--GCAAAACGTG
Idn2                GATGTTCTGGGCCGCACGCGCGTTACAATGGCATGT-AAAGCGAGTGCTT
CJ1_1               GATGTTCTGGGCCGCACGCGCGTTACAATGGCACGT-AAAGCGAGTGCTT
CR_1                GATGTTCTGGGCCGCACGCGCGTTACAATGGCACGT-AAAGCGAGTGCTT
Pdidermoides        GATGTTCTGGGCCGCACGCGCGTTACAATGGCATGT-AAAACGAGTGCTT
UFF1                GATGTTCTGGGCCGCACGCGCGTTACAATAGCATGT-AAAACGNGTGCTT
CJA3                GATGTTCTGGGCCGCACGCGCGTTACAATGGCATGT-AAAGCGAGTGCTT
Prigidum            GATGTTCTGGGCCGCACGCGCGTTACAATGGCATGA-AAAATGAGTGCTT
C1                  GATGTTCTGGGCCGCACGCGCGTTACAATGGCATGT-AAAGCAAGTGCTT
PpolycephalumWis1   GATGTTCTGGGCCGCACGCGCGTTACAATGGCATGT-AAAACGAGTGCTT
Sflavogenita        GATGTTCCGGGCCGCACGCGCGTTACAATGGGGGGC-AAAACGAGTGTCC

CUR1_4              TCAAGGCTTTTCCGCGGCCTACTGGCCGCGGTAATCCCTAGTCTCTCCCT
HA4_1               TCAAGGCGTTTCCGCGGCCGACGGGTCGCGGTAACCCCTAATCTCTCCCT
CR19_1              ACACGGCTTCTCCGCGGCCTACTGGTCGCGGTAATCCCTAATCTCTCCCT
Pan2                ATTCGGCTTCTCCGCGGCCTACTGGC----GTAATCCTTAATCTCTCCCT
CR8_1               ATTCGGCTTCTCCGCGGCCTACTGGC----GTAATCCTTAATCTCTCCCT
Fr_K7               TCTACGCGTTTCCGGGGCCGACGGGTCCCGGTAACCCCTAGTCTCTCCCT
Fr_K15              TTTACGCGTTTCCGGGGCCGACGGGTCCCGGTAACCCCTAGTCTCTCCCT
CR10                TTTAGGCGTCTCCACGGCCGACGGGTCGTGGTAACCCCTAATCTCTCCCT
It_K61              AAAAGGCGTATCCGGGGCCTAACGGCTTCGGTAACCCTTAGTCCCTGCCT
Fr_K10              AAAAGGCGTATCCGGGGCCTACAGGCTCTGGTAACCCCTAGTCCCTGCCT
It_K66              AAAAGGCGTATCCGGGGCCTACAGGCTCTGGTAACCCTTAGTCCCTGCCT
Uk_K79              AAAAGGCGTATCCGGGGCCTACAGGCTCTGGTAACCCCTAGTCCCTGCCT
Pr3_1               ACTAAGCGTATCCGGGGCCTAACGGTCCCGGTAACCCTTAGTCCCTGCCT
It_K71              AAAAGGCGTATCCGCGGCCTACAGGTCGCGGTAACCCCTAGTCCCTGCCT
Fr_K18              AAAAGGCGTCTCCGCGGCCTACAGGCTGCGGTAACCCCTAGTCCCTGCCT
Fr_K62              AAAAGGCGTATCCGCGGCCTACAGGTCGCGGTAACCCCTAGTCCCTGCCT
No_K94              ACAAGGCGTATCCGCGGCCTACGGGTCGCGGTAACCCKTAGTCCCTGCCT
Cur_1               GAAAAGCGTCTCCGGGGCCGAAAGGCCCTGGTAACCCTTAGTCCCTGCCT
Az4_1               GAAAAGCGTCTCCGGGGCCGAAAGGCCCTGGTAACCCTTAGTCCCTGCCT
PR_1                GAAAAGCGTCTCCGGGGCCGAAAGGCCCTGGTAACCCTTAGTCCCTGCCT
Butricularis        GAATAGCGTCCCCGGGGCCGAAAGGTCTCGGTAACCCTTAGTCCTTGCTT
NY_1                GTTAAGCATATCCGGGGCCGAAAGGTTCTGGTAACCATTAGTCCCTGCTT
Poblonga            CCTTCGTTTCCCCTCGGTCGAAAGGCCTTGGTAACCGCTAACCCTTGCTA
Idn2                ATCCAGCTTTGCCGGGGCCGAAAGGCTTCGGTAATCCTTAATCCCTGCCT
CJ1_1               CTCCAGCTTTGCCGGGGCCGAAAGGTCCTGGTAATCCTTAATCCCTGCCT
CR_1                CTCCAGCTTTGCCGGGGCCGAAAGGTCCTGGTAATCCCTAATCCCTGCCT
Pdidermoides        CTCCAGCTTTGCCGGGGCTGAAAGGTCTCGGTAATCCTTAATCCCTGCCT
UFF1                GAATAGCGTCTCCGGGGCCGAAAGGTCCCGGTAACCCTTAGTCCTTGCTT
CJA3                GA-AAGCGTCTCCGGGGCCGAAAGGCCCTGGTAACCCTTAGTCCCTGCCT
Prigidum            GCATAGCGTCCCCTGGGCCGAAAGGTCTGGGTAACCCTTAGTCCCTGCTT
C1                  TTCAAGCGTCTCCGGGGCCGAAAGGCTCCGGTAACCCTTAGTCCCTGCCT
PpolycephalumWis1   GAACAGCGTCCC-ACGGCCGAAAGGTCGTGGTAACCCTTAGTCCCTGCTT
Sflavogenita        GTCAGGCTTTTCCGCCGCCTACCGGTGGTGGTAACCCGTAGGGCCCCCCT
```

```
CUR1_4              TGACTGGGCCAGACTATTGCAATTATTGGTCTCCAACGAGGAATTTTTAG
HA4_1               TGACTGGGACAGACTATTGCAATTATTGGTCTCCAACGAGGAATTTTTAG
CR19_1              TGACTGGGACAGACTATTGCAATTATTGGTCTCAAACGAGGAATTTTTAG
Pan2                TGACTGGGACAGACTATTGCAATTATTGGTCTCCAACGAGGAATTTTTAG
CR8_1               TGACTGGGACAGACTATTGCAATTATTGGTCTCCAACGAGGAATTTTTAG
Fr_K7               TGACTGGGCCAGACTATTGCAATTATTGGTCTCAAACGAGGAATTTTTAG
Fr_K15              TGACTGGGCCAGACTATTGCAATTATTGGTCTCAAACGAGGAATTTTTAG
CR10                TGACTGGGACAGACTATTGCAATTATTGGTCTCCAACGAGGAATTTTTAG
It_K61              TGACTGGGACAGACTATTGCAATTATTGGTCTCAAACGAGGAATTTTTAG
Fr_K10              TGACTGGGACAGACTATTGCAATTATTGGTCTCAAACGAGGAATTTTTAG
It_K66              TGACTGGGACAGACTATTGCAATTATTGGTCTCAAACGAGGAATTTTTAG
Uk_K79              TGACTGGGACAGACTATTGCAATTATTGGTCTCAAACGAGGAATTTTTAG
Pr3_1               TGACTGGGACAGACTATTGCAATTATTGGTCTCAAACGAGGAATTTTTAG
It_K71              TGACTGGGACAGACTATTGCAATTATTGGTCTCAAACGAGGAATTTTTAG
Fr_K18              TGACTGGGACAGACTATTGCAATTATTGGTCTCAAACGAGGAATTTTTAG
Fr_K62              TGACTGGGACAGACTATTGCAATTATTGGTCTCAAACGAGGAATTTTTAG
No_K94              TGACTGGGACAGACTATTGTAATTATTGGTCTCAAACGAGGAATTTTTAG
Cur_1               TGACTGGGACAGATCATTGCAATTATTGGTCTCCAACGAGGAATTTT-AG
Az4_1               TGACTGGGACAGATCTTTGCAATTATTGGTCTCCAACGAGGAATTTTTAG
PR_1                TGACTGGGACAGATCATTGCAATTATTGGTCTCCAACGAGGAATTTTTAG
Butricularis        TGACTGGGACAGATCTTTGCAATTATTGGTCTCAAACGAGGAATTTTTAG
NY_1                AGACTGGGACAGATCATTGCAATTATTGGTCTCAAACGAGGAATTTTTAG
Poblonga            TGACTGGGATAGATCATTGCAATTATTGGTCTCTAACGAGGAATTTTTAG
Idn2                TGACTGGGATAGATCATTGCAATTATTGGTCTCAAACGAGGAATTTTTAG
CJ1_1               TGACTGGGATAGATCATTGCAATTATTGGTCTCCAACGAGGAATTTTTAG
CR_1                TGACTGGGATAGATCATTGCAATTATTGGTCTCCAACGAGGAATTTTTAG
Pdidermoides        TGACTGGGATAGATCATTGCAATTATTGGTCTCCAACGAGGAATTTTTAG
UFF1                TGACTGGGACAGATCTTTGCAACTATTGGTCTCTAACGAGGAATTTTTAG
CJA3                TGACTGGGACAGATCATTGCAATTATTGGTCTCCAACGAGGAATTTTTAG
Prigidum            TGACTGGGACAGATCATTGCAATTATTGGTCTCAAACGAGGAATTTTTAG
C1                  TGACTGGGACAGATCTTTGCAATTATTGGTCTCAAACGAGGAATTTTTAG
PpolycephalumWis1   TGACTGGGACAGATCTTTGCAATTATTGGTCTCAAACGAGGAATTTTTAG
Sflavogenita        TGACTGGGCTAGTCTCTTGCAATTATTGGTCTTCAACGAGGAATTTTTAG

CUR1_4              TAGTCGCAGGTCATTAACCTGCGACGAATGCGTCCCTGCCCTTTGTACAC
HA4_1               TAGTCGCAGGTCATTAACCTGCGACGAATGCGTCCCTGCCCTTTGTACAC
CR19_1              TAGTCGCAGGTCATTAACCTGCGGCGAATGCGTCCCTGCCCTTTGTACAC
Pan2                TAGTCGCAGGTCATTAACCTGCGACGAATGCGTCCCTGCCCTTTGTACAC
CR8_1               TAGTCGCAGGTCATTAACCTGCGACGAATGCGTCCCTGCCCTTTGTACAC
Fr_K7               TAATCGCAGGTCATTAACCTGCGATGAATGCGTCCCTGCCCTTTGTACAC
Fr_K15              TAATCGCAGGTCATTAACCTGCGATGAATGCGTCCCTGCCCTTTGTACAC
CR10                TAGTCGCAGGTCATTAACCTGCGACGAATGCGTCCCTGCCCTTTGTACAC
It_K61              TAGTCGTAGGTCATTAACCTGCGACGAATGCGTCCCTGCCCTTTGTACAC
Fr_K10              TAGTCGTAGGTCATTAACCTGCGACGAATGCGTCCCTGCCCTTTGTACAC
It_K66              TAGTCGTAGGTCATTAACCTGCGACGAATGCGTCCCTGCCCTTTGTACAC
Uk_K79              TAGTCGTAGGTCATTAACCTGCGACGAATGCGTCCCTGCCCT--------
Pr3_1               TAGTCGCAGGTCATTAACCTGCGACGAATGCGTCCCT-----TTGTACAC
It_K71              TAGTCGCAGGTCATTAACCTGCGACGAATGCGTCCCTGCCCTTTGTACAC
Fr_K18              TAGTCGCAGGTCATTAACCTGCGACGAATGCGTCCCTGCCCTTTGTACAC
Fr_K62              TAGTCGCAGGTCATTAACCTGCGACGAATGCGTCCCTGCCCTTTGTACAC
No_K94              TAGTCGCAGGTCATYAGCCTGCGACGAATGCGTCCCTGCCCTTTGTACAC
Cur_1               TAGTCGCAGGTCATTAACCTGCGACGAATGCGTCCCTGCCCTTTGTACAC
Az4_1               TAGTCGCAGGTCATTAACCTGCGACGAATGCGTCCCTGCCCTTTGTACAC
PR_1                TAGTCGCAGGTCATTAACCTGCGACGAATGCGTCCCTGCCCTTTGTACAC
Butricularis        TAATCGTAGGTCATTAACCTGCGTTGAATGCGTCCCTGCCCTTTGTACAC
NY_1                TAAGCATAGGTCATTAACCTTTGCTGAATGAGTCCCTGCCTTTTGTACAC
Poblonga            TAATCGCAGGTCATTAACCTGCGATGAATCTGTCCCTGCCCTTTGTACAC
Idn2                TAGGCGCAGGTCATTAACCTGCGTCGAATGCGTCCCTGCCCTTTGTACAC
CJ1_1               TAGGCGCAGGTCATTAACCTGCGTCGAATGCGTCCCTGCCCTTTGTACAC
CR_1                TAGGCGCAGGTCATTAACCTGCGTCGAATGCGTCCCTGCCCTTTGTACAC
Pdidermoides        TAGGCGCAGGTCATTAACCTGCGTCGAATGCGTCCCTGCCCTTTGTACAC
UFF1                TAATCGTAGGTCATTAACCTGCGTTGAATGCGTCCCTGCCCTTTGTACAC
CJA3                TAGTCGCAGGTCATTAACCTGCGACGAATGCGTCCCTGCCCTTTGTACAC
Prigidum            TAATCGTAGGTCATTAACCTGCGTTGAATGCGTCCCTGCCCTTTGTACAC
C1                  TAGTCGTAGGTCATTAACCTACGACGAATGCGTCCCTGCCCTTTGTACAC
PpolycephalumWis1   TAATCGCAGGTCATTAACCTGCGTTGAATGCGTCCCTGCCCTTTGTACAC
Sflavogenita        TAGGCGCGGGTCATTAACCCGTGCCGAATGCGTCCCTGCCCTTTGTACAC
```

```
CUR1_4             ACCGCCCGTCGCTGCTACCGATTGGGTTTTGCGGTTACGAGTTCGCAGGT
HA4_1              ACCGCCCGTCGCTGCTACCGATTGGGTTTTGCGGTTACGAGTTCANAGGC
CR19_1             ACCGCCCGTCGCTGCTACCGATTGGGTTTTGCGGTTACGAGTTCGGACGT
Pan2               ACCGCCCGTCGCTGCTACCGATTGGGTTTTGCGGTTACGAGTTCGGAGGC
CR8_1              ACCGCCCGTCGCTGCTACCGATTGGGTTTTGCGGTTACGAGTTCGGAGGC
Fr_K7              ACCGCCCGTCGCTGCTACCGATTGGGTTTTGCGGTTACGAGTTCCGAGGT
Fr_K15             ACCGCCCGTCGCTGCTACCGATTGGGTTTTGCGGTTACGAGTTCCGAGGT
CR10               ACCGCCCGTCGCTGCTACCGATTGGGTTTTGCGGTTACGAGTTCGGAGAC
It_K61             ACCGCCCGTCGCTGCTACCGATTGGGTTTTGCGGTTACGAGTTCAGAGGA
Fr_K10             ACCGCCCGTCGCTGCTACCGATTGGGTTTTGCGGTTACGAGTTCGGAAGA
It_K66             ACCGCCCGTCGCTGCTACCGATTGGGTTTTGCGGTTACGAGTTCGGAAGA
Uk_K79             -------GTCGCTGCTACCGATTAGGTTTTGCGGTTACGAGTTCGGAAGA
Pr3_1              ACCGCCCGTCGCTGCTACCGATTGGGTTTTGCGGTTACGCGTTCCGA-GA
It_K71             ACCGCCCGTCGCTGCTACCGATTGGGTCCTGCGGTTACGAGTTCGGAGGC
Fr_K18             ACCGCCCGTCGCTGCTACCGATTGGGTCCTGCGGTTACGAGTTCGGAGGC
Fr_K62             ACCGCCCGTCGCTGCTACCGATTGGGTCCTGCGGTTACGAGTTCGGAGGC
No_K94             ACCGCCCGTCGCTGCTACCGATTGGGTTTTGCGGTTACGAGTTCGGAGGT
Cur_1              ACCGCCCGTCGCTGCTACCGATTGGGTTTCACAGTTACGTGTTCGGAGGT
Az4_1              ACCGCCCGTCGCTGCTACCGATTGGGTTTTACAGTTACGTGTTTGGAGGC
PR_1               ACCGCCCGTCGCTGCTACCGATTGGGTTTCACAGTTACGTGTTCGGAGGT
Butricularis       ACCGCCCGTCGCTGCTACCGATTGGGTTTTACAGTTACGCGTTCGGAGGA
NY_1               ACCGCCCGTCGCTGCTACCGATTGGGTTTTACAGTTACGCGTTCGGAGGT
Poblonga           ACCGCCCGTCGCTGCTACCGATTGGGTTTTACAGTTACGCGTTTGGAGGT
Idn2               ACCGCCCGTCGCTGCTACCGATTGAATTTTCCAGTTACGCGTTCGGAGCA
CJ1_1              ACCGCCCGTCGCTGCTACCGATTGAATTTTCCAGTTACGCGTTCGGAGAA
CR_1               ACCGCCCGTCGCTGCTACCGATTGAATTTTCCAGTTACGCGTTCGGAGAA
Pdidermoides       ACCGCCCGTCGCTGCTACCGATTGAATTTTCCAGTTACGCGTTCAGAGCA
UFF1               ACCGCCCGTCGCTGCTACCGATTGGGTTTCGCAGTTACGCGTTCGGAGGA
CJA3               ACCGCCCGTCGCTGCTACCGATTGGGTTTCACAGTTACGTGTTCGGAGGT
Prigidum           ACCGCCCGTCGCTGCTACCGATTGGGTTTCACAGTTACGCGTTCAGAGGA
C1                 ACCGCCCGTCGCTGCTACCGATTGGGTTTTACAGTTACGTGCTTGGAGGC
PpolycephalumWis1  ACCGCCCGTCGCTGCTACCGATTGGGTTTTACAGTTACGCGTTCGGAGGA
Sflavogenita       ACCGCCCGTCGCTGCTACCGATTGGGTTTCGCGGTTACGTATTCGGAGAT

CUR1_4             CG-TGGTCATCG-C--CCGCAAGGGCGATGGC--TTGCAGCTGAAGTTC-
HA4_1              CG-TGGTTACCGCC--CTCA-CGGGTGGTGG---CCGTAGCTGAAGTTC-
CR19_1             GC-TGGTTATTGAC--CCGCAAGGGGCTTAGC--T-GCTGCCAAAGTTC-
Pan2               AG-CGGTTATCGCC--CCGCAAGGGCGATAGC--A-GCAGCCAAAGTTC-
CR8_1              AG-CGGTTATCGCC--CCGCAAGGGCGATAGC--A-GCAGCCAAAGTTC-
Fr_K7              CG-CGGCCGCCGCC--CCCGCAAGGGGGTGGTGGCTGCAGCCGAAGTCC-
Fr_K15             CG-CGGCCGCCGCC--CCCGCAAGGGGGTGGTGGCTGCAGCCGAAGTCC-
CR10               CG-TGGCTCCCGCC--TTCTTCGGGAGGTGGATACCGCGGTCGAAGTTCA
It_K61             CG-CGGCTCCTGCCTGCCTTCGGGTAGGCAGTAACTGTGACCAAAGTTC-
Fr_K10             TG-TGGCTCCTGCCTGCCTTCGGGTAGGTAGTAACTATGACTAAAGTTC-
It_K66             TG-TGGCTCCTGCCTGCCTTCGGGTAGGTAGTAACTATGACTAAAGTTC-
Uk_K79             TG-TGGCTCCTGCCTGCCTTCGGGTAGGTAGTAACTATGACTAAAGTTC-
Pr3_1              CT-AGGCGTCTGTCTGCCTTCGGGTAGGTAGTAGCC-TGGGATAAGTTC-
It_K71             TGGTGGTGCCCGCTTGCCTCACGGTAGGCGGGYGCCTCGG-TGAAGTTC-
Fr_K18             CGTCGGTGCCCGCTTGCCTCACGGTAGGCGGGCGCCATGGCCAAAGTTC-
Fr_K62             TGGTGGTGCCCGCTTGCCTCACGGTAGGCGGGTGCCTCGGCTGAAGTTC-
No_K94             CG-TGGCTCCCGTTTGT-TCACG-CAGGCGGTAACCATAGCCAAAGTCC-
Cur_1              GACGGCCCCCCGCTTGCCTCACGGCGGGCGGGAGCCGACGCCGAAGTTCC
Az4_1              GCTGGCCCCCCGCTTGCTTCACGGTAGGCGGGAGCCGGTACCAAAATCCA
PR_1               GACGGCCCCCCGCTTGCCTCACGGCGGGCGGGAGCCGACGCCGAAGTTCC
Butricularis       TGGTGCTTTCGGGCC--TTCAC-GGG-TCCGGGGCATCGTCCGAAGTTCT
NY_1               TGTGG--TTCTGCTTGCC-TTCGGGTAGGTAGGGCCGTGACTGAAGTTCT
Poblonga           CGTGGCTTTTTGCTTGCCCTTTGGGTAGGCATGACCGTGGCTGAAATTCT
Idn2               GGGGAGTACTGACCC-----GC-AAGGGTTAGTCTCTCGCGTAAAGTCCT
CJ1_1              TGGGGGGTCTGGCCC-----GC-AAGGGTTAGCCTCCTGAGCAAAGTCCT
CR_1               TGGGGGGTCTGGCCC-----GC-AAGGGTTAGCCTCCTGAGCAAAGTCCT
Pdidermoides       TGATGGTATCGACTC-----GC-AAGGGTTGGTCTATCGGGCAAAGTCCT
UFF1               TGGTGCCCTCGTGTCCCTTCACTGGGGTGCGGGGCGTCGTCCGAAGTTCT
CJA3               GACGGCCCCCCGCTTGCCTCACGGCGGGCGGGAGCCGACGCCGAAGTTCC
Prigidum           TGGTGCTCTCGGGT---TTCAC-GA--CCTGGGGCGTCGACCGAAGTTCT
C1                 CGTGGTTCCCTGCTTGCTCTCGGGTAGGCAGGTGCCGTAGCCGAAATTCN
PpolycephalumWis1  CGATGTTTTCGGGTC--TTAAC-GGGCTCCGGGGCGTCGGCCAAAGTTCT
Sflavogenita       TCTTTCAAC-TGCTG--CCGCAAGGCGGCGGGCGACTGGGTCTAAGTCTA
```

```
CUR1_4             TAATTACCGCACGGCCTAGAGTAACTATGACTCTCTTAAGGTAGCCAAAT
HA4_1              TAATTACCGCACGGCCTAGAGTAACTATGACTCTCTTAAGGTAGCCAAAT
CR19_1             TAATTACCGCACGGCCTAGAGTAACTATGACTCTCTTAAGGTAGCCAAAT
Pan2               TAATTACCGCACGGCCTAGAGTAACTATGACTCTCTTAAGGTAGCCAAAT
CR8_1              TAATTACCGCACGGCCTAGAGTAACTATGACTCTCTTAAGGTAGCCAAAT
Fr_K7              TAATTACCGCACGGCCTAGAGTAACTATGACTCTCTTAAGGTAGCCAAAT
Fr_K15             TAATTACCGCACGGCCTAGAGTAACTATGACTCTCTTAAGGTAGCCAAAT
CR10               TAATTACCGCACGGCCTAGAGTAACTATGACTCTCTTAAGGTAGCCAAAT
It_K61             TAATTACCGCACGGCCTAGAGTAACTATGACTCTCTTAAGGTAGCCAAAT
Fr_K10             TAATTACCGCACGGCCTAGAGTAACTATGACTCTCTTAAGGTAGCCAAAT
It_K66             TAATTACCGCACGGCCTAGAGTAACTATGACTCTCTTAAGGTAGCCAAAT
Uk_K79             TAATTACCGCACGGCCTAGAGTAACTATGACTCTCTTAAGGTAGCCAAAT
Pr3_1              CAATTACCGCACGGTCTAGAGTAACTATGACTCTCTTAAGGTAGCCAAAT
It_K71             TAATTACCGCATGGCCTAGAGTAACTATGACTCTCTTAAGGTAGCCAAAT
Fr_K18             TAATTACCGCATGGCCTAGAGTAACTATGACTCTCTTAAGGTAGCCAAAT
Fr_K62             TAATTACCGCA-GGCCTAGAGTAACTATGACTCTCTTAAGGTAGCCAAAT
No_K94             TAATTACCGCACGGCCTAGAGTAACTATGACTCTCTTAAGGTAGCCAAAT
Cur_1              -AACTACTGTGCGGCCTAGAGTAACTATGACTCTCTTAAGGTAGCCAAAT
Az4_1              -AATTACTGTACGGCCTAGAGTAACTATGACTCTCTTAAGGTAGCCAAAT
PR_1               -AACTACTGTGCGGCCTAGAGTAACTATGACTCTCTTAAGGTAGCCAAAT
Butricularis       -AATTACTGTACGGCCTAGAGTAACTATGACTCTCTTAAGGTAGCCAAAT
NY_1               -AATTACTGTACGGCCTAGAGTAACTATGACTCTCTTAAGGTAGCCAAAT
Poblonga           -AACTACTGTACGGCCTAGAGTAACTATGACTCTCTTAAGGTAGCCAAAT
Idn2               -AATTACTGCACGGTTTAGAGTAACTATGACTCTCTTAAGGTAGCCAAAT
CJ1_1              -AATTACTGCACGGTTTAGAGTAACTATGACTCTCTTAAGGTAGCCAAAT
CR_1               -AATTACTGCACGGTTTAGAGTAACTATGACTCTCTTAAGGTAGCCAAAT
Pdidermoides       -AATTACTGCACGGTTTAGAGTAACTATGACTCTCTTAAGGTAGCCAAAT
UFF1               -AACTACTGCGCGGCCTAGAGTAACTATGACTCTCTTAAGGTAGCCAAAT
CJA3               -AATTACTGTGCGGCCTAGAGTAACTATGACTCTCTTAAGGTAGCCAAAT
Prigidum           TAATTACTGTGCGGCCTAGAGTAACTATGACTCTCTTAAGGTAGCCAAAT
C1                 -AATTACTGTACGGCCTAGAGTAACTATGACTCTCTTAAGGTAGCCAAAT
PpolycephalumWis1  -AACTACTGTACGGCCTAGAGTAACTATGACTCTCTTAAGGTAGCCAAAT
Sflavogenita       -AATTACCGCACGGCCTAGAGTAACTATGACTCTCTTAAGGTAGCCAAAT

CUR1_4             GCCTCGTCATTTAATTTGTGACGCGCATGAATGGATTAATGAGATTCCCA
HA4_1              GCCTCGTCATTTAATTTGTGACGCGCATGAATGGATTAATGAGATTCCCA
CR19_1             GCCTCGTCATTTAATTTGTGACGCGCATGAATGGATTAATGAGATTCCCA
Pan2               GCCTCGTCATTTAATTTGTGACGCGCATGAATGGATTAATGAGATTCCCA
CR8_1              GCCTCGTCATTTAATTTGTGACGCGCATGAATGGATTAATGAGATTCCCA
Fr_K7              GCCTCGTCATTTAATTTGTGACGCGCATGAATGGATTAATGAGATTCCCA
Fr_K15             GCCTCGTCAT--AATTTGTGACGCGCATGAATGGATTAATGAGATTCCCA
CR10               GCCTCGTCATTTAATTTGTGACGCGCATGAATGGATTAATGAGATTCCCA
It_K61             GCCTCGTCATTTAATTTGTGACGCGCATGAATGGATTAATGAGATTCCCA
Fr_K10             GCCTCGTCATTTAATTTGTGACGCGCATGAATGGATTAATGAGATTCCCA
It_K66             GCCTCGTCATTTAATTTGTGACGCGCATGAATGGATTAATGAGATTCCCA
Uk_K79             GCCTCGTCATTTAATTTGTGACGCGCATGAATGGATTAATGAGATTCCCA
Pr3_1              GCCTCGTCATTTAATTTGTGACGCGCATGAATGGATTAATGAGATTCCCA
It_K71             GCCTCGTCATTTAATTTGTGACGCGCATGAATGGATTAATGAGATTCCCA
Fr_K18             GCCTCGTCATTTAATTTGTGACGCGCATGAATGGATTAATGAGATTCCCA
Fr_K62             GCCTCGTCATTTAATTTGTGACGCGCATGAATGGATTAATGAGATTCCCA
No_K94             GCCTCGTCATTTAATTTGTGACGCGCATGAATGGATTAATGAGATTCCCA
Cur_1              GCCTCGTCATTTAATTTGTGACGCGCATGAATGGATTAATGAGATTCCCA
Az4_1              GCCTCGTCATTTAATTTGTGACGCGCATGAATGGATTAATGAGATTCCCA
PR_1               GCCTCGTCATTTAATTTGTGACGCGCATGAATGGATTAATGAGATTCCCA
Butricularis       GCCTCGTCATTTAATTTGTGACGCGCATGAATGGATTAATGAGATTCCCA
NY_1               GCCTCGTCATTTAATTTGTGACGCGCATGAATGGATTAATGAGATTCCCA
Poblonga           GCCTCGTCAT----------ACGCGCATGAATGGATTAATGAGATTCCCA
Idn2               GCCTCGTCATTTAATTTGTGACGCGCATGAATGGATTAATGAGATTCCCA
CJ1_1              GCCTCGTCAT--AATTTGTGACGCGCATGAATGGATTAATGAGATTCCCA
CR_1               GCCTCGTCATTTAATTTGTGACGCGCATGAATGGATTAATGAGATTCCCA
Pdidermoides       GCCTCGTCATT-AATTTGTGACGCGCATGAATGGATTAATGAGATTCCCA
UFF1               GCCTCGTCATTTAATTTGTGACGCGCATGAATGGATTAATGAGATTCCCA
CJA3               GCCTCGTCATTTAATTTGTGACGCGCATGAATGGATTAATGAGATTCCCA
Prigidum           GCCTCGTCATTTAATTTGTGACGCGCATGAATGGATTAATGAGATTCCCA
C1                 GCCTCGTCAT--AATTTGTGACGCGCATGAATGGATTAATGAGATTCCCA
PpolycephalumWis1  GCCTCGTCATTTAATTTGTGACGCGCATGAATGGATTAATGAGATTCCCA
Sflavogenita       GCCTCGTCAT-TAATTTGTGACGCGCATGAATGGATTAATGAGATTCCCA
```

```
CUR1_4             CTGTCCCTACCTACTATCTAGCGAAACCACAGCCAAGGGAACGGGCTTGG
HA4_1              CTGTCCCTACCTACTATCTAGCGAAACCACAGCCAAGGGAACGGGCTTGG
CR19_1             CTGTCCCTACCTACTATCTAGCGAAACCACAGCCAAGGGAACGGGCTTGG
Pan2               CTGTCCCTACCTACTATCTAGCGAAACCACAGCCAAGGGAACGGGCTTGG
CR8_1              CTGTCCCTACCTACTATCTAGCGAAACCACAGCCAAGGGAACGGGCTTGG
Fr_K7              CTGTCCCTACCTACTATCTAGCGAAACCACAGCCAAGGGAACGGGCTTGG
Fr_K15             CTGTCCCTACCTACTATCTAGCGAAACCACAGCCAAGGGAACGGGCTTGG
CR10               CTGTCCCTACCTACTATCTAGCGAAACCACAGCCAAGGGAACGGGCTTGG
It_K61             CTGTCCCTACCTACTATCTAGCGAAACCACAGCCAAGGGAACGGGCTTGG
Fr_K10             CTGTCCCTACCTACTATCTAGCGAAACCACAGCCAAGGGAACGGGCTTGG
It_K66             CTGTCCCTACCTACTATCTAGCGAAACCACAGCCAAGGGAACGGGCTTGG
Uk_K79             CTGTCCCTACCTACTATCTAGCGAAACCACAGCCAAGGGAACGGGCTTGA
Pr3_1              CTGTCCCTACCTACTATCTAGCGAAACCACAGCCAAGGGAACGGGCTTGG
It_K71             CTGTCCCTACCTACTATCTAGCGAAACCACAGCCAAGGGAACGGGCTTGG
Fr_K18             CTGTCCCTACCTACTATCTAGCGAAACCACAGCCAAGGGAACGGGCTTGG
Fr_K62             CTGTCCCTACCTACTATCTAGCGAAACCACAGCCAAGGGAACGGGCTTGG
No_K94             CTGTCCCTACCTACTATYTAGCGAAACCACAGCCAAGGGAACGGGCTTGG
Cur_1              CTGTCCCTACCTACTATCTAGCGAAACCACAGCCAAGGGAACGGGCTTGG
Az4_1              CTGTCCCTACCTACTATCTAGCGAAACCACAGTCAAGGGAACGGTCTTGA
PR_1               CTGTCCCTACCTACTATCTAGCGAAACCACAGCCAAGGGAACGGGCTTGG
Butricularis       CTGTCCCTACCTACTATCTAGCGAAACCACAGCCAAGGGAACGGGCTTGG
NY_1               CTGTCCCTACCTACTATCTAGCGAAACCACAGCCAAGGGAACGGGCTTGG
Poblonga           CTGTCCCTACCTACTATCTAGCGAAACCACAGCCAAGGGAACGGGCTTGG
Idn2               CTGTCCCTACCTACTATCTAGCGAAACCACAGCCAAGGGAACGGGCTTGG
CJ1_1              CTGTCCCTACCTACTATCTAGCGAAACCACAGTCAAGGGAACGGGCTTGA
CR_1               CTGTCCCTACCTACTATCTAGCGAAACCACAGTCAAGGGAACGGGCTTGA
Pdidermoides       CTGTCCCTACCTACTATCTAGCGAAACCACAGCCAAGGGAACGGGCTTGG
UFF1               CTGTCCCTACCTACTATCTAGCGAAACCACAGCCAAGGGAACGGGCTTGG
CJA3               CTGTCCCTACCTACTATCTAGCGAAACCACAGCCAAGGGAACGGGCTTGG
Prigidum           CTGTCCCTACCTACTATCTAGCGAAACCACAGCCAAGGGAACGGGCTTGG
C1                 CTGTCCCTACCTACTATCTAGCGAAACCACAGCCAAGGGAACGGGCTTGG
PpolycephalumWis1  CTGTCCCTACCTACTATCTAGCGAAACCACAGCCAAGGGAACGGGCTTGG
Sflavogenita       CTGTCCCTACCTACTATCTAGCGAAACCACAGCCAAGGGAACGGGCTTGG

CUR1_4             CACAATTAGCGGGGAAAGAAGACCCTGTTGAGCTTGACTCTAGGCACAAA
HA4_1              CACAATTAGCGGGGAAAGAAGACCCTGTTGAGCTGGACTCTAGGCACAAA
CR19_1             CACAATTAGCGGGGAAAGAAGACCCTGTTGAGCTTGACTCTAGGCACAAA
Pan2               CACAATTAGCGGGGAAAGAAGACCCTGTTGAGCTTGACTCTAGGCACAAA
CR8_1              CACAATTAGCGGGGAAAGAAGACCCTGTTGAGCTTGACTCTAGGCACAAA
Fr_K7              CCCAATTAGCGGGGAAAGAAGACCCTGTTGAGCTTGACTCTAGGCACAGA
Fr_K15             CCCAATTAGCGGGGAAAGAAGACCCTGTTGAGCTTGACTCTAGGCACAGA
CR10               CACAATTAGCGGGGAAAGAAGACCCTGTTGAGCTTGACTCTAGGCACAAA
It_K61             CACAATTAGCGGGGAAAGAAGACCCTGTTGAGCTTGACTCTAGGCACAGA
Fr_K10             CACAATTAGCGGGGAAAGAAGACCCT-TTGAGCTTGACTCTAGGCACAGA
It_K66             CACAATTAGCGGGGAAAGAAGACCCTGTTGAGCTTGACTCTAGGCACAGA
Uk_K79             CACAATTAGCGGGGAAAGAAGACCCTGTTGAGCTTGACTCTAGGCACAGA
Pr3_1              CACAATTAGCGGGGAAAGAAGACCCTGTTGAGCTTGACTCTAGGCACAGA
It_K71             CACAATTAGCGGGGAAAGAAGACCCTGTTGAGCTTGACTCTAGGCACAGA
Fr_K18             CACAATTAGCGGGGAAAGAAGACCCTGTTGAGCTTGACTCTAGGCACAGA
Fr_K62             CACAATTAGCGGGGAAAGAAGACCCTGTTGAGCTTGACTCTAGGCACAGA
No_K94             CACAATTAGCGGGGAAAGAAGACCCTGTTGAGCTTGACTCTAGGCACAAA
Cur_1              CACAATTAGCGGGGAAAGAAGACCCTGTTGAGCTTGACTCTAGGCACAGA
Az4_1              CACAATTTGCGGGGAAAGAAGACCCTGTTGAGCTTGACTCTAGGCATAGA
PR_1               CACAATTAGCGGGGAAAGAAGACCCTGTTGAGCTTGACTCTAGGCACAGA
Butricularis       CACAATTAGCGGGGAAAGAAGACCCTGTTGAGCTTGACTCTAGGCATAGA
NY_1               CACAATTAGCGGGGAAAGAAGACCCTGTTGAGCTTGACTCTAGGCACAGA
Poblonga           CACAATTAGCGGGGAAAGAAGACCCTGTTGAGCTTGACTCTAGGCACAAA
Idn2               CACAATTAGCGGGGAAAGAAGACCCTGTTGAGCTTGACTCTAGGCATAGA
CJ1_1              CACAATTAGCAGGGAAAGAAGACCCTGTTGAGCTTGACTCTAGGCATAGA
CR_1               CACAATTAGCGGGGAAAGAAGACCCTCTTAATCTTGACTCTAGGCATAGA
Pdidermoides       CACAATTAGCGGGGAAAGAAGACCCTGTTGAGCTTGAGTCTAGGCATAGA
UFF1               CACAATTAGCGGGGAAAGAAGACCCTGTTGAGCTTGACTCTAGGCATAGA
CJA3               CACAATTAGCGGGGAAAGAAGACCCTGTTGAGCTTGACTCTAGGCACAGA
Prigidum           CACAATTAGCGGGGAAAGAAGACCCTGTTGAGCTTGACTCTAGGCATAGA
C1                 CACAATTAGCGGGGAAAGAAGACCCTGTTGAGCTTGACTCTAGGCACAAA
PpolycephalumWis1  CACAATTAGCGGGGAAAGAAGACCCTGTTGAGCTTGACTCTAGGCATAGA
Sflavogenita       CACAATTAGC-GGGAAAGAAGACCCTGTTGAGCTTGACTCTAGGCACAGA
```

```
CUR1_4              CGCGAGGTGATTCTAAAGGTGTAGCATAGGTGGGAGGGCCTGGCCCGACC
HA4_1               CGCGAGGTGATTCTAAAGGTGTAGCATAGGTGGGAGGGCCTGGCCCGACC
CR19_1              CGCGAGGTGATTCTAAAGGTGTAGCATAGGTGGGAGGACCTGGTCCGACC
Pan2                CGCGAGGTGATTCTAAAGGTGTAGCATAGGTGGGAGG-CCTGGCCCGACC
CR8_1               CGCGAGGTGATTCTAAAGGTGTAGCATAGGTGGGAGG-CCTGGCCCGACC
Fr_K7               AGCGAGGTGATTCTAAAGGTGTAGCATAGGTGGGAGGGCCTGGCCCGACC
Fr_K15              AGCGAGGTGATTCTAAAGGTGTAGCATAGGTGGGAGGGCCTGGCCCGACC
CR10                AGCGAGGTGATTCTAAAGGCGTAGCATAGGTGGGAGGACCTGGTCCGACC
It_K61              AGCGAGGTGATTCTAAAGGTGTAGCATAGGTGGGAGGGCCTGGCCCGACC
Fr_K10              AGCGAGGTGATTCTAAAGGTGTAGCATAGGTGGGAGGGCCTGGCCCGACC
It_K66              AGCGAGGTGATTCTAAAGGTGTAGCATAGGTGGGAGGGCCTGGCCCGACC
Uk_K79              AGCGAGGTGATTCTAAAGGTGTAGCATAGGTGGGAGGGCCTGGCCCGACC
Pr3_1               AGCGAGGTGATTCTAAAGGTGTAGCATAGGTGGGAGGGCCTGGCCCGACC
It_K71              AGCGAGGTGATTCTAAAGGTGTAGCATAGGTGGGAGGACCTGGTCCGCCC
Fr_K18              AGCGAGGTGATTCTAAAGGTGTAGCATAGGTGGGAGGACCTGGTCCGCCC
Fr_K62              AGCGAGGTGATTCTAAAGGTGTAGCATAGGTGGGAGGACCTGGTCCGCCC
No_K94              AGCGAGGTGATTCTAAAGGTGTAGCATAGGTGGGAGGGCCTGGCCCGACC
Cur_1               CGCGAGGTGATTCTAAAGGTGTAGCATAGGTGGGAGGGCCCAGCCCGACC
Az4_1               CGCGAGGTGATTCTAAAGGTGTAGCATAGGTGGGAGGGCCCAGCCCGATC
PR_1                CGCGAGGTGATTCTAAAGGTGTAGCATAGGTGGGAGGGCCCAGCCCGACC
Butricularis        AGCGAGGTGATTCTAAAGGTGTAGCATAGGTGGGAGGGTCCATCCCGACA
NY_1                AGCGAGGTGATTCTAAAGGTGTAGCATAGGTGGGAGGGCCCAG-CCGTCC
Poblonga            CGCGAGGTGATTCTAAAGGTGTAGCATAGGTGGGAGGGCCCATCCCGACC
Idn2                CGCGAGGTGATTCTAAAGGTGTAGCATAGGTGGGAGGGCCCAGCCCGATC
CJ1_1               CGCGAGGTGATTCTAAAGGTGTAGCATAGGTGGGAGGGCCCAGCCCGATC
CR_1                CGCCAGGTGATTCTAAAGGTGTAGCATAGGTGGGAGGGCCCAGCCCGATC
Pdidermoides        CGCGAGGTGATTCTAAAGGTGTAGCATAGGTGGGAGGGCCCGATC----C
UFF1                AGCGAGGTGATTCTAAAGGTGTAGCATAGGTGGGAGAGCACATCTCGCCA
CJA3                CGCGAGGTGATTCTAAAGGTGTAGCATAGGTGGGAGGGCCCAGCCCGACC
Prigidum            CGCGAGGTGATTCTAAAGGTGTAGCATAGGTGGGAGGGCCCCTCCCGTCC
C1                  CGCGAGGTGATTCTAAAGGTGTAGCATAGGTGGGAGGGCACAGCCCGACC
PpolycephalumWis1   CGCGAGGTGATTCTAAAGGTGTAGCATAGGTGGGAGGGCCCATCCCGTCA
Sflavogenita        TGCGAGGTGATTCTAAAGGTGTAGCATAG-CGGGAGGGCTCTCTCCGACC

CUR1_4              TTGAAATACCACCACTTTCGACATTGCCTTGCTAATGCTGTAACAAATGG
HA4_1               TTGAAATACCACCACTTTCGACATTGCTTTGCTAATGCTGTAATGAATGA
CR19_1              TTGAAATACCACCACTTTCGACATTGCCTTGCTAATGCTGTAAAAAGTGA
Pan2                TTGAAATACCACCACTTTCGACATTGCCTTGCTAATGCTGTAATAAGTAA
CR8_1               TTGAAATACCACCACTTTCGACATTGCCTTGCTAATGCTGTAATAAGTAA
Fr_K7               TTGAAATACCACCACTTTCGACATCGCTTTGCTAATGCTGTAACGAATGG
Fr_K15              TTGAAATACCACCACTTTCGACATCGCTTTGCTAATGCTGTAACGAATGG
CR10                CTGAAATACCACCACTTTCGACATTGCCTTGCTAATGCTGTAACGAATGA
It_K61              TTGAAATACCACCACTTTCGACATCGCTTTGCTAATGCTGTAACGAACGA
Fr_K10              TTGAAATACCACCACTTTCGACATCGCTTTGCTAATGCTGTAACGAACGA
It_K66              TTGAAATACCACTACTTTCGACATCGCTTTGCTAATGCTGTAACGAACGA
Uk_K79              TTGAAATACCACCACTTTCGACATCGCTTTGCTAATGCTGTAACGAACGA
Pr3_1               TTGAAATACCACCACTTGAGACATTGCTTTGCTAATGCTGTAACGAACGA
It_K71              TTGAAATACCACCACTTTCGACATTGCTTTGCTAATGCTGTAACGAACGA
Fr_K18              TTGAA-TACCACCACTTTCGACATTGCTTTGCTAATGCTGTAACGAACGA
Fr_K62              TTGAAATACCACCACTTTCGACATTGCTTTGCTAATGCTGTAACGAACGA
No_K94              TTGAAATACCACCACTTTCGACATTGCTTTGCTAATGCTGTAACGAACGA
Cur_1               TTGAAATACCACCACTTTCGACATCGCTTTGCTAATGCTGTAACGAACGA
Az4_1               CTGAAATACCACCACTTTCGACATCGCTTTGCTAATGCTGTAACGAATGA
PR_1                TTGAAATACCACCACTTTCGACATCGCTTTGCTAATGCTGTAACGAACGA
Butricularis        ATGAAATACCACCACTTTCGACATCGCTTTGCTAATGCTGTAACGAACGA
NY_1                ATGAAATACCACCACTTTCGACATTGCTTTGCTAATGCTGTAACGAACGA
Poblonga            TTGAAATACCACCACTTTAGACATTGCTTTGCTAATGCTGTAACGAATGA
Idn2                CTGAAATACCACCACTTTCGACATCGCTTTGCTAATGCTGTAACGAATGA
CJ1_1               CTGAAATACCACCACTTTCGACATCGCTTTGCTAATGCTGTAACGAATGA
CR_1                CTGAAATACCACCACTTTCGACATCGCTTTGCTAATGCTGTAACGAATGA
Pdidermoides        CTGAAATACCACCACTTTCGACATCGCTTTGCTAATGCTGTAACGAATGA
UFF1                ATGAAATACCACCACTTTCGATATTGCTTTGCTAATGCTGTAACGAACGA
CJA3                TTGAAATACCACCACTTTCGACATCGCTTTGCTAATGCTGTAACGAACGA
Prigidum            ATGAAATACCACCACTTTCGACATCGCTTTGCTAATGCTGTAACGAACGA
C1                  TTGAAATACCACCACTTTCGACATTGCTTTGCTAATGCTGTAACGAACAA
PpolycephalumWis1   ATGAAATACCACCACTTTCGACATCGCTTTGCTAATGCTGTAACGAACGA
Sflavogenita        CTGAAATACCGCCACTTTCGACATTGCTTTGCTAATGCTGTAACGAACGA
```

```
CUR1_4              ACGGTCCCCCTTCGTGTCGGCTCCTCACGGTAAGATCATGTTCTGGCGTT
HA4_1               ACGACCCCCTATCGTGTCGGCTGCCTTCGGTAAGGTTATATTCTGGATTT
CR19_1              ACGGCCCCCCTTCTCGTTGGCTCCTCGCGGTAAGGCTATGTTCTGGCGTT
Pan2                ACGACCCCCCTTCGTGTCGGCCTTCACGGGTACGGTCATGTTCTGGCGTT
CR8_1               ACGACCCCCCTTCGTGTCGGCCTTCACGGGTACGGTCATGTTCTGGCGTT
Fr_K7               ACGGCCCCCCGATGACTCGGTTGCCTCCGGTAAGGCTTCTTTCTGGTGAT
Fr_K15              ACGGCCCCCCGATGACTCGGTTGCCTCCGGTAAGGCTTCTTTCTGGTGAT
CR10                ACGGCCCCCTGTTTGTCGTGCCTCACGGGGTAAGGTCATATTCTGGTGTT
It_K61              ACGGCCCCCGGGCCCGTCACCCGCAAGGGGTAAGGTCATATTCTAGCGCT
Fr_K10              ACGACCCCCAGGACCGTTGCCCGCAAGGGGTAAGGTCATATTCTAGCGCT
It_K66              ACGACCCCCAGGACCGTTGCCCGCAAGGGGTAAGGTCATATTCTAGCGCT
Uk_K79              ACGACCCCCAGGACCGTTGCCCGCAAGGGGTAAGGTCATATTCTAGCGCT
Pr3_1               ACGGCCCCCGGGCCGCCATCCGCAAGGGGGTACGGTCATATTCTAGCGCT
It_K71              ACGGCCCCCGGGCCGCTTGGGCCGCAAGGGTAAGGTCATATTCTAGCGCT
Fr_K18              ACGGCCCCCGGGCTGTTCAGGCCGCAAGGGTAAGGTCATATTCTAGCGCT
Fr_K62              ACGGCCCCCGGGCCGCTTGGGCCGCAAGGGTAAGGTCATATTCTAGCGCT
No_K94              ACGGCCCCCTGGACGTCGGTGCCCTCGCGGTAAGGTCATCTTCTAGCGCT
Cur_1               ACGGCCCCTCGGTGTCCCGCTCGAAAGGGGTAAGGTCACATTCTAGCGCT
Az4_1               ACGACCCCCCTTCTGAAGCCCGCAAGGGGGTAAGGTCATATTCTGGCGTC
PR_1                ACGGCCCCTCGGTGTCCCGCTCGAAAGGGGTAAGGTCACATTCTAGCGCT
Butricularis        ACGGAACCGCGTCCCTCTCGTCGAAAGGGGATAGGCCAAATTCTAGCGCT
NY_1                ACGACACCCAAGGGCTTTACGCACCTAGGGTAAG-TCATATTCTAGCGTT
Poblonga            ACGGCCCCCTGATATCCTGCTCGGAAGGGGTAAGGTCATATTCTAGTGCT
Idn2                ACGATCCCCCGTTCCAGAGGTCCGCAAGGGTAAGATCATATTCTGGCGTC
CJ1_1               ACGA-CCCCCCTTCTGAAGCCCGCAAGGGGTAAGGTCATATTCTGGCGTC
CR_1                ACGACCCCCGTTCTGAAGCCGCAAGGTGGGTAAGGTCATATTCTGGCGTC
Pdidermoides        ACGACCCCTCGTCCTGAAGCTCGCAAGAGGTAAGGTCAGATTCTGGTGTC
UFF1                ACGCAACCGCGTCCCTCTCGTCGAAAGAGGATAGGCCAAATTCTAGCGCT
CJA3                ACGACCCCTCGGTGTCCCGCTCGAAAGGGGTAAGGTCACATTCTAGCGCT
Prigidum            ACGGAACCGCGTCCCTCTCGTCGCAAGAGGATAGGCCAAATTCTAGCGCT
C1                  ACGGCCCCCTGGTTCCCCGTCCGCAAGGGGTAAGGTCATATTCTAGCGTT
PpolycephalumWis1   ACGGAACCGCGTCCCTCTCACCGTAAAAGGATAGGCCAAATTCTAGCGCT
Sflavogenita        ACGGCCCCCCGGGTTCGGCCGCCTTCACGGATCGGTCACATTCTGGCTTT
```

```
CUR1_4              TTCCCGGTTCGTCATGATGGCTTGGCTACCCTGGATAAGTACCCTAGGGC
HA4_1               TTCCTGGTTTGCCACGATGGTCTGGCTACCCTGGGTGAGTACCCTGGGAC
CR19_1              TTCCTTGCTGGTCACGATGGCTTGGCTACCTTGGGTTAGTACCTCGGGGC
Pan2                TTCCCTGCTGGTAATGATGGCGTGGCTACCTCGGAGAAGTACCCTGAGGC
CR8_1               TTCCCTGCTGGTAATGATGGCGTGGCTACCTCGGAGAAGTACCCTGAGGC
Fr_K7               TTCCCGGTCTGATACGGTGGTTTGGCCCCTCTGGGATAGTACCTCGAGAC
Fr_K15              TTCCCGGTCTGATACGGTGGTTTGGCCCCTCTGGGATAGTACCTCGAGAC
CR10                TTCCCGGTTGGTCATGATGGTCTGGCCCCTCTGGGCGAGTACCCTGGGAC
It_K61              TTCCTAGTCGGATACGATGGCTTGGCTCCTCTGGGTGAGTACCTTGGGAC
Fr_K10              TTCCTCGTTGGGTATGATGGTTTGGCTCCTCTGGGATAGTACTTCGAAGC
It_K66              TTCCTCGTTGGGTACGATGGTTTGGCTCCTCTGGGATAGTACTTCGGGAC
Uk_K79              TTCCTCGTTGGGTACGATGGTTTGGCTCCTCTGGGATAGTACTTCGGGAC
Pr3_1               TTCCTAGTCGAGTACGACGGCTTGGCTCCTCTGGGTGAGTACCCTGGGAC
It_K71              TTCCTGGCTATACAAGGTGGCTTGGCTCCTCTGGGTGAGTACTCTGGGAC
Fr_K18              TTCCTGGCTGGTCATGATGGCTTGGCTCCTCTGGGTGAGTACTCTGGGAC
Fr_K62              TTCCTGGCTAATCAAGACGGCTTGGCCCCTCTGGGTGAGTACTCTGGGAC
No_K94              TTCCTAGCTAGGTACGGTGGTTTGGCCCCTCTGGGTGAGTACCTTGGGAC
Cur_1               TTCCCGACTCG-TACGGTGGCCTGGCCCCTCCGGGTGAGTACCTCGTGAC
Az4_1               GTCCTCGATCG-CATGACGAGTCGGCTACCCTGGGATAGTACTTCNAATC
PR_1                TTCCCGACTCG-TACGGTGGCCTGGCCCCTCCGGGTGAGTACCTCGTGAC
Butricularis        TTCCGCGCTCG-CATGACGGATCGGCTCCTCTGGGTGAGTACTGTGGGAC
NY_1                TTCCAGGCACG-T-CGATGGGTCAAACCCTCTGGGTGAGTACCTCGAGAC
Poblonga            TTCCGAGCTCG-TATGATGGCTTGGGCTCTCTGGGATAGTACTTTGGGAC
Idn2                TTCCACGGTTG-CATGATGGGTTAGCTAACCTAGGATAGTACTTCGGATC
CJ1_1               GTCCTCGATCG-CATGACGAGTCGGCTACCCTGGGATAGTACTTCGAATC
CR_1                GTCCTCGATCG-TACGACGGGTTGGCTACCCTGGGATAGTACTTCGAATC
Pdidermoides        CTCCATAATCG-TACGATTGGTTGGCTAACCTAGGATAGTACTTCGAGTC
UFF1                TTCCGCGCCCG-CATGACGGATTGGCTCCCCTGGGTGAGTACTGCGAGAC
CJA3                TTCCCGACTCG-TACGGTGGCCTGGCCCCTCCGGGTGAGTACCTCGTGAC
Prigidum            TTCCACGCTCT-CATGATGTGGCGGCTCCCCTGGGTGAGTACCGCGAGAC
C1                  TTCCGGGTTCA-CACGATGGCTTGGCTCCTCTGGGTGAGTACTCTGGGAC
PpolycephalumWis1   TTCCGCGCTCA-TACGACGAGTTGGCTCCCCTGGGTGAGTACCGTGGAAC
Sflavogenita        TGCTCGGCTGGCTATGACTGGGTCCAGCCTGCGGGTGAGTACCTTGGGGC
```

```
CUR1_4             CGCAACGTGGGCTTCCGGGCTCGCAGCGCGTTGTTCTTGGGGGAACTGGG
HA4_1              CGTGAGTCGGGCTTCCGAGTCTGGCGAGCGCTGTTCTCGGGGGAACTAGG
CR19_1             TACAAGCCGGGCTTCCGGGCTCGGTGAGTATTGTTCTCGGGGGAACTGAG
Pan2               CGCAATCCAGGCTTTCGGGCTCGGTGAGCGCTGTTCTCGGGGGAACCGGG
CR8_1              CGCAATCCAGGCTTTCGGGCTCGGTGAGCGCTGTTCTCGGGGGAACCGGG
Fr_K7              CATGAGTTGGGCTTCCGAGCCCGGCGAGTGTTGTT-TTGGGGGAACCAGC
Fr_K15             CATGAGTTGGGCTTCCGAGCCCGGCGAGTGTTGTT-TTGGGGGAACCAGC
CR10               CGTGCGTTGGGCTTCCGAGTCTGACAAACGTTGCTCCCGGGGGAACCAGG
It_K61             CGCAAACTGGGCTTCCGGGTCTGGTGCGCGTTGTTTCCGGGGGAACCAGG
Fr_K10             TGCAAACTGGGCTTCCGGGTTCGGTGCGCAT-GTT-TTGGGGGAACCAGG
It_K66             TGCAAACTGGGCTTCCGGGTTCGGTGCGCATTGTT-CTGGGGGAACCAGG
Uk_K79             TGCAAACTGGGCTTCCGGGTTCGGTGCGCATTGTT-CTGGGGGAACCAGG
Pr3_1              CGCAAGTTGGGCTTCCGGGTCTGACGAGCGTTGCTCCTGGGGGAACCAGG
It_K71             CGCAAACTGGGCTTCCGGGTCTGGTGCGCGCTGCTCTCGGGGGAACCAGA
Fr_K18             CGCAAACTGGGCTTCCGGGTCTGGTGCGCGCTGCTCTTGGGGGAACCAGA
Fr_K62             CGCAAACTGGGCTTCCGGGTCTGGTGCGCGCTGCTCTCGGGGGAACCAGA
No_K94             CACGAGCTGGGCTTACGAGTCTGGTGAGTGTCGCTTCCGGGGGAACCAGG
Cur_1              CACAAACTGGGCCTCCGGGTCTGGTGCGTGTCGTTCTCGGGGGAACCGGA
Az4_1              CGTAATTCGGGCTTCCGGGTCTGGAGAGCGCTGTGTTTGGGGGAACTAGG
PR_1               CACAAACTGGGCCTCCGGGTCTGGTGCGTGTCGTTCTCGGGGGAACCGGA
Butricularis       CGCGAGTCGGGCTTCCGAGTCTGGCGAGTGCTGCTTCTGCGGGAACCAGG
NY_1               CACAAACTGGGCTTCCGGGTTTGGTGCGTGTTGCTCTTG-GGGAACTGGA
Poblonga           CGCAAACTGGGCTTCCGGGTTCGGTGCGTGTTGTTTCTGAGGGAACCAGA
Idn2               CACGAGACGGGCTTCCGGGTCTGGCGAGTGTTGCGTTCGGGGGAACTAGG
CJ1_1              CGTAATTCGGGCTTCCGGGTCTGGAGAGCGCTGTGTTTGGGGGAACTAGG
CR_1               CGCAATTCGGGCTTCCGGGTCTGGAGCGCGCTGTGTTTGGGGGAACCAGG
Pdidermoides       CGTGAGCTGGGCTTCCGGGTCTGGCAAGCGTTGTGCTTGGGGGAACTAGG
UFF1               CGTAAGTTGGGCTTCCGAGTCCGGCGCGCGTTGTTCTTGCGGGAACCAGG
CJA3               CACAAACTGGGCCTCCGGGTCTGGTGCGTGTCGTTCTCGGGGGAACCGGA
Prigidum           CGTACGCTGAGCTTCCGAGCCCGGTAAACGCTGTCCTCGTGGGAACCAGA
C1                 CACAAACTGGGCCTCCGGGTTCGGTGCGTGCTGCTTCTGGGGGAACCAGG
PpolycephalumWis1  CACGAGCTGGGCTTCCGAGTCCGGCGAGTGCTGTTTCCGCGGGAACCAGA
Sflavogenita       GGCAATTGGGGCTTCCGGGTTCCGAGCGCATTGTT-CTGGGGGAACCGTG

CUR1_4             GATC-GCTGGGTTGTCCTCT-GGCGGGCGTTAACGGTACATAAGGTGTAG
HA4_1              GCTT-GTTGGGCTGTCCTCT-GGTGGGCGTTAAAGGTACATAAGGTGTAG
CR19_1             GGTCCGCTGGGTTGTCCTCT-GGCTGGCTTCGAGGGTACACAAGGTGTAG
Pan2               GGTTCGTTGTGCTGTCCTCT-GGCCGGCGTAAAGGGTATACAAGGTGTAG
CR8_1              GGTTCGTTGTGCTGTCCTCT-GGCCGGCGTAAAGGGTATACAAGGTGTAG
Fr_K7              GACG-GTTGAGCTGTCCTCT-GTCGGACCTCGTAGGTACATAAGGTGTAG
Fr_K15             GACG-GTTGAGCTGTCCTCT-GTCGGACCTCGTAGGTACATAAGGTGTAG
CR10               GATG-GTTGGACTGTCCTCT-GTCTGGCCTCGGTGGTACATAAGGTGTAG
It_K61             GATG-GTTGGGCTGTCCTCT-GTCCGGCGAAATCGGTACGTAAGGTGTAG
Fr_K10             GATG-GTTGGACTGTCCTCT-GCCTGGCAATTTTGGTACGTAAGGTGTAG
It_K66             GATG-GTTGGACTGTCCTCT-GCCTGGCAATTTTGGTACGTAAGGTGTAG
Uk_K79             GATG-GTTGGACTGTCCTCT-GCCTGGCAATTTTGGTACGTAAGGTGTAG
Pr3_1              GATA-GTTGGGCTGTCCTCT-GCTTGGCGATTCGGGTACGTAAGGTGTAN
It_K71             GATG-GTTGGGCTGCCCTCT-GGATAGCATAAATGGTACGTAAGGTGTAG
Fr_K18             GATG-GTTGGGCTGCCCTCT-GTCTGGCGTCAAAGGTACGTAAGGTGTAG
Fr_K62             GATG-GTTGGGCTGCCCTCT-GTATAGCATAAATGGTACGTAAGGTGTAG
No_K94             GACG-GTTGGATCACCCTCT-GCCTGGCGTAATCGGTACGTTAGGTGTAG
Cur_1              GACGG-TTGGGCCGCCCTCT-GCGGGCTAAAAG-GGTACGTAAGGTGTAG
Az4_1              TGTGA-TGGCTGCGTCCTCT-GCGGTCTTTATT-GGTACATAGT--GTAG
PR_1               GACGG-TTGGGCCGCCCTCT-GCGGGCTAAAAG-GGTACGTAAGGTGTAG
Butricularis       GACGG-TCGAACTGTCCTCT-GCGGGCTTCAAGCGGTACGTAATGTGTAG
NY_1               GGTCGTGCGA-CTGTCCTCT-GCGGGCATTAAACGGTACGTAATGTGTAG
Poblonga           GATCGCTTGGGCTGTCCTCT-ATGGGGTCAAGGAGGTACATAAAGTGTAG
Idn2               CGTGA--GAGCTCATCCTCT-GCAGCCAAAAT--GGTACATAGTCAGTAG
CJ1_1              TGTGA--TGGCGCGTCCTCT-GCGGTCTTTATT-GGTACATAGTCAGTAG
CR_1               TGTGA--TAACACGTCCTCT-GCGGTCTTTATT-GGTACATAGTCAGTAG
Pdidermoides       CGTNA--TGACTGGTCCTCT-GCGGTTTAATATTGGTACATAGTCAGTAG
UFF1               GACGGGTCGAACCGTCCTCT-GTGGGCTTTAAATGGTACGTAATGTGTAG
CJA3               GACGG-TTGGGCTGCCCTCT-GCGGGCTAAAAG-GGTACGTAAGGTGTAG
Prigidum           GACGG-TCGTCGTGTCCTCT-GAGGGCTTCAAGTGGTACGTAAGGTGTAG
C1                 GATGG-TTGGGCTGTCCTCT-GTGGGCGAATATCGGTATGTAAGGTGTAG
PpolycephalumWis1  GACGG-TCTTCTCGGCCTCT-GTGGGCTTCATGCGGTACGTAATGTGTAG
Sflavogenita       GGTGTGGGGCTTGGTCCTCTAGCCGGCAATTTCAGGCACGTAACGTGTAG
```

```
CUR1_4             CAGACTATTT-GTGTTAGGGAGTTT-GGCTGGGGCGGAAAACTGCTACAC
HA4_1              CAGACTATTT-GTGTTGGGGAGTTT-GGCTGGGGCGGAAAACTGCTACAC
CR19_1             CAAACTATTTTGTGTTAGGGAGTTTTGGCTGGGGCGGAAAACTGCTACAC
Pan2               CAAACTATTT-GTGTTAGGGAGTTT-GGCTGGGGCGGAAAACTGCTACAC
CR8_1              CAAACTATTT-GTGTTAGGGAGTTT-GGCTGGGGCGGAAAACTGCTACAC
Fr_K7              CAAACCATCT-GTGTTGGGGAGTTT-GGCTGGGGCGGAAAACTGCTACAC
Fr_K15             CAAACCATCT-GTGTTGGGGAGTTT-GGCTGGGGCGGAAAATTGCTACAC
CR10               CAAACCATTT-GTGTTGGGGAGTTT-GGCTGGGGCGGAAAACTGCTACAC
It_K61             CAGACCATCT-GTGTTGGGGAGTTT-GGCTGGGGCGGAAAACTGCTACAC
Fr_K10             CAGACTATCT-GTGTTGGGGAGTTT-GGCTGGGGCGGAAAACTGCTACAC
It_K66             CAGACTATCT-GTGTTGGGGAGTTT-GGCTGGGGCGGAAAACTGCTACAC
Uk_K79             CAGACTATCT-GTGTTGGGGAGTTT-GGCTGGGGCGGAAAACTGCTACAC
Pr3_1              CAGACCATCT-GTGTTGGGGAGTTT-GGCTGGGGCGGAAAACTGCTACAC
It_K71             CAGACCATCT-GTGTTGGGGAGTTT-GGCTGGGGCGGAAAACTGCTACAC
Fr_K18             CAGACACTCT-GTGTTGGGGAGTTT-GGCTGGGGCGGAAAACTGCTACAC
Fr_K62             CAGACCATCT-GTGTTGGGGAGTTT-GGCTGGGGCGGAAAACTGCTACAC
No_K94             CAGACCATTT-GTGTTGGGGAGTTT-GGCTGGGGCGGAAAACTGCTACAC
Cur_1              CAAACCATC-TGTGTTGGGGAGTTT-GGCTGGGGCGGAAAACTGCTACAC
Az4_1              CAGACTATCCTATGTTGGGGAGTTT-GGCTGGGGCGGAAAACTGCTACAC
PR_1               CAAACCATC-TGTGTTGGGGAGTTT-GGCTGGGGCGGAAAACTGCTACAC
Butricularis       CAGACTATC-TATGTTGGGGAGTTT-GGCTGGGGCGGAAAACTGCTACAC
NY_1               CAGACACTC-TGTGTTGGGGAGTTT-GGCTGGGGCGGAAAACTGCTACAC
Poblonga           CAGACTCTT-TGTGTTAGGGAGTTT-GGCTGGGGCGGAAAACTGCTACAT
Idn2               CAGGCTATC-TATGTTGGGGAGTTT-GGCTGGGGCGGAAACCTACCACAC
CJ1_1              CAGACTATC-TATGTTGGGGAGTTT-GGCTGGGGCGGAAAACTGCTACAC
CR_1               CAGACTATC-TATGTTGGGGAGTTT-GGCTGGGGCGGAAAACTGCTACAC
Pdidermoides       CAGACTATC-TATGTTGGGGAGTTT-GGCTGGGGCGGAAAACTGCTACAC
UFF1               CAGACTATC-TATGTTGGGGAGTTT-GGCTGGGGCGGAAAACTGCTACAC
CJA3               CAAACCATC-TGTGTTGGGGAGTTT-GGCTGGGGCGGAAAACTGCTACAC
Prigidum           CAGACTATC-TATGTTGGGGAGTTT-GGCTGGGGCGGAAAACTGCTACAC
C1                 CAGACCATT-TGTGTTGGGGAGTTT-GGCTGGGGCGGAAAACTGCTACAC
PpolycephalumWis1  CAGACTATC-TATGTTGGGGAGTTT-GGCTGGGGCGGAAAACTGCTACAC
Sflavogenita       CAGAAACAC-TGTGTTGGGGAGTTT-GGCTGGGGCGGAAAACTGCTACAT

CUR1_4             GGCAACGGCAGTCTCCTAAGGTCCACTCAGAGACGACAGAAACGTCT-CG
HA4_1              GGCAACGGCAGTCTCCTAAGGTCCACTCAGAGACGACAGAAACGTCT-CG
CR19_1             GGCAACGGCAGTCTCCTAAGGTCCACTCAGAGACGACAGAAACGTCT-CG
Pan2               GGCAACGGCAGTCTCCTAAGGTTCACTCAGAGACGACAGAAACGTCT-CG
CR8_1              GGCAACGGCAGTCTCCTAAGGTTCACTCAGAGACGACAGAAACGTCT-CG
Fr_K7              GGCAACGGCAGTCTCCTAAGGTCCACTCAGAGACGACAGAAACGTCT-CG
Fr_K15             GGCAACGGCAGTCTCCTAAGGTCCACTCAGAGACGACAGAAACGTCT-CG
CR10               GGCAACGGCAGTCTCCTAAGGTCCACTCAGAGACGACAGAAACGTCT-CG
It_K61             GGCAACGGCAGTCTCCTAAGGTCCACTCAGAGACGACAGAAACGTCT-CG
Fr_K10             GGCAACGGCAGTCTCCTAAGGTCCACTCAGAGACGACAGAAACGTCT-CG
It_K66             GGCAACGGCAGTCTCCTAAGGTCCACTCAGAGACGACAGAAACGTCT-CG
Uk_K79             GGCAACGGCAGTCTCCTAAGGTCCACTCAGAGACGACAGAAACGTCT-CG
Pr3_1              GGCAACGGCAGTCTCCTAAGGTCCACTCAGAGACGACAGAAACGTCT-CG
It_K71             GGCAACGGCAGTCTCCTAAGGTCCGCTCAGAGACGACAGAAACGCCT-CG
Fr_K18             GGCAACGGCAGTCTCCTAAGGTCCGCTCAGAGACGACAGAAACGCCT-CG
Fr_K62             GGCAACGGCAGTCTCCTAAGGTCCGCTCAGAGACGACAGAAACGCCT-CG
No_K94             GGCAACGGCAGTCTCC-AAGGTCCACTCAGAGACGACAGAAACGTCT-CG
Cur_1              GGCAACGGCAGTCTCCTAAGGTCCACTCAGAGACGACAGAAACGTCTTCG
Az4_1              GGCAACGGCAGTCTCCTAAGGTCCACTCAGAGACGACAGAAACGTCT-CG
PR_1               GGCAACGGCAGTCTCCTAAGGTCCACTCAGAGACGACAGAAACGTCT-CG
Butricularis       GGCAACGGCAGTCTCCTAAGGTCCACTCAGAGACGACAGAAACGTCT-CG
NY_1               GGCAACGGCAGTCTCCTAAGGTCCACTCAGAGACGACAGAAACGTCT-CG
Poblonga           GGCAACGGCAGTCTCCTAAGGTCCACTCAGAGACGACAGAAAC-TCT-CG
Idn2               GGCAACGGCAGTCTCCTAAGGTCCACTCAGAGACGACAGAAACGTCT-CG
CJ1_1              GGCAACGGCAGTCTCCTAAGGTCCACTCAGAGACGACAGAAACGTCT-CG
CR_1               GGCAACGGCAGTCTCCTAAGGTCCACTCAGAGACGACAGAAACGTCT-CG
Pdidermoides       GGCAACGGCAGTCTCCTAAGGTCCACTCAGAGACGACAGAAACGTCT-CG
UFF1               GGCAACGGCAGTCTCCTAAGGTCCGCTCAGAGACGACAGAAACGTCT-CG
CJA3               GGCAACGGCAGTCTCCTAAGGTCCACTCAGAGACGACAGAAACGTCT-CG
Prigidum           GGCAACGGCAGTCTCCTAAGGTCCACTCAGAGACGACAGAAACGTCT-CG
C1                 GGCAACGGCAGTCTCCTAAGGTCCACTCAGAGACGACAGAAACGTCT-CG
PpolycephalumWis1  GGCAACGGCAGTCTCCTAAGGTCCACTCAGAGACGACAGAAACGTCT-CG
Sflavogenita       GGCAACGGCAGTCTCCTAAGGTCTGCTCAGAGGCGACAGAAACGTCT-CG
```

```
CUR1_4             TAGAGCATAAGGGCAAAAGTGGGCTTAACTCACATTTTCA-GTAGTAATG
HA4_1              TAGAGCATAAAGGCAAAAGTGGGCTTAACTTACATTTTCA-GTAGTAATG
CR19_1             TAGAGCATAAGGGCAAAAGTGGGCTTAACTTACATTTTCA-GTAGTAATG
Pan2               TAGAGCATAAGGGCAAAAGTGAGCTTAACTCACATTTTCA-GTAGTAATG
CR8_1              TAGAGCATAAGGGCAAAAGTGAGCTTAACTCACATTTTCA-GTAGTAATG
Fr_K7              TAGAGCATAAAGGCAAAAGTGGGCTTAACTTACATTTTCA-GTAGTAATG
Fr_K15             TAGAGCATAAAGGCAAAAGTGGGCTTAACTTACATTTTCA-GTAGTAATG
CR10               TAGAGCATAAAGGCAAAAGTGGGCTTAACTTACATTTTCA-GTAGTAATG
It_K61             TAGAGCATAAAGGCAAARGTGGGCTTAACTCGCATTTTCA-GTAGTAATG
Fr_K10             TAGAGCATAAAGGCAAAAGTGGGCTTAACTCGCATTTTCA-GTAGTAATG
It_K66             TAGAGCATAAAGGTAAAAGTGGGCTTAACTCGCATTTTCA-GTAGTAATG
Uk_K79             TAGAGCATAAAGGCAAA-GTGGGCTTAACTCGCATTTTCA-GTAGTAATG
Pr3_1              TAGAGCATAAAGGCAAAAGTGGGCTTAACTTACATTCTCA-GTAGTAATG
It_K71             TAGAGCATAAAGGCAAAAGCGGGCTTAACTTACATTTTCA-GTAGTAATG
Fr_K18             TAGAGCATAAAGGCAAAAGCGGGCTTAACTTACATTTTCA-GTAGTAATG
Fr_K62             TAGAGCATAAAGGCAAAAGCGGGCTTAACTTACATTTTCA-GTAGTAATG
No_K94             TAGAGCATAAAGGCAAAAGTGGGCTTAACTTACATTCTCA-GTAGTAATG
Cur_1              TAGAGCATAAAGGCAAAAGTGGGCTTAACTCGCATTTTCA-GTAGTAATG
Az4_1              TAGAGCATAAAGGCAAAAGTGGGCTTAACTCACGTTTTCA-GTAGTAATG
PR_1               TAGAGCATAAAGGCAAAAGTGGGCTTANGTCGCATTTTCA-GTAGTAATG
Butricularis       TAGAGCATAAAGGCAAAAGTGGGCTTAACTCGCATTTTCAAGTAGTAATG
NY_1               TAGAGCATAAAGGCAAAAGTGGGCTTAACTCACGTTTTCA-GTAGTAATG
Poblonga           TAGAGCATAAAGGCAAAAGTGGGCTTAACTCGCATTTT-A-GTAGTAATG
Idn2               TAGAGCATAAAGGCAAAAGTGGGCTTAACTCACGTTTTCA-GTAGTAATG
CJ1_1              TAGAGCATAAAGGCAAAAGTGGGCTTAACTCACGTTTTCA-GTAGTAATG
CR_1               TAGAGCATAAAGGCAAAAGTGGGCTTAACTCACGTTTTCA-GTAGTAATG
Pdidermoides       TAGAGCATAAAGGCAAAAGTGGGCTTAACTCACGTTTTCA-GTAGTAATG
UFF1               TAGAGCATAAAGGCAAAAGCGGGCTTAACTCGCATTTTCA-GTAGTAATG
CJA3               TAGAGCATAAAGGCAAAAGTGGGCTTAACTCGCATTTTCA-GTAGTAATG
Prigidum           TAGAGCATAAAGGCAAAAGTGGGCTTAACTCGCATTTTCA-GTAGTAATG
C1                 TAGAGCATAAAGGNAAAAGTGGGCTTAACTCGCATTTTCA-GTAGTAATG
PpolycephalumWis1  TAGAGCATAAAGGCAAAAGTGGGCTTAACTCGCATTTTCA-GTAGTAATG
Sflavogenita       TAGAACATAAAGGTAAAAGCAGGCTTAAATTACATTCTCA-GTAGTAATG

CUR1_4             TGAAGCAAGAAATTGAGGCCTAACGATCCTTAACGGCGGGTGCCAGCCCA
HA4_1              TGAAGCAAGAAATTGAGGCTTAACGATCCTTAACGTCGGGTGCCAGCCCA
CR19_1             TGAAGCAAGAAATTGAGGCCTAACGATCCTTAACGGCGGGTGCCAGCCCA
Pan2               TGAAGCAAGAAATTGAGGCCTAACGATCCTTAACGGCGGGTGCCAGCCCA
CR8_1              TGAAGCAAGAAATTGAGGCCTAACGATCCTTAACGGCGGGTGCCAGCCCA
Fr_K7              TGAAGCAAGAAATTGAGGCTTAACGATCCTTAGCGTCGGGTGCCAGCCCG
Fr_K15             TGAAGCAAGAAATTGAGGCTTAACGATCCTTAGCGTCGGGTGCCAGCCCG
CR10               TGAAGCAAGAAATTGAGGCTTAACGATCCTTAACGTCGGGTGCCAGCCCG
It_K61             TGAAGCAAGAAATTGAGGCTTAACGATCCTTAGCAGCGGGTGCCAGCCCA
Fr_K10             TGAAGCAAGAAATTGAGGCTTAACGATCCTTAGCAGCGGGTGCCAGCCCA
It_K66             TGAAGCAAGAAATTGAGGCTTAACGATCCTTAGCAGCGGGTGCCAGCCCA
Uk_K79             TGAAGCAAGAAATTGAGGCTTAACGATCCTTAGCAGCGGGTGCCAGCCCA
Pr3_1              TGAAGCAAGAAATTGAGGCTTAACGATCCTTAGCAGCGGGTGCCAGCCCA
It_K71             TGAAGCAAGAAATTGAGGCTTAACGATCCTTAGCGTCGGGTGCCAGCCCA
Fr_K18             TGAAGCAAGAAATTGAGGCTTAACGATCCTTAGCGTCGGGTGCCAGCCCA
Fr_K62             TGAAGCAAGAAATTGAGGCTTAACGATCCTTAGCGTCGGGTGCCAGCCCA
No_K94             TGAAGCAAGAAATTGAGGCTTAACGGTCCTTAACGTCGGGTGCCAGCCCG
Cur_1              TGAAGCAAGAAATTGAGGCTTAACGATCCTTAACGGCGGGTGCCAGCCCA
Az4_1              TGAAGCAAGAAATTGAGGCTTAACGATCCTTAGCGGCGGGTGCCAGCCCA
PR_1               TGAAGCAAGAAATTGAGGCTTAACGATCCTTAACGGCGGGTGCCAGCCCA
Butricularis       TGAAGCAAGAAATTGCGGCTTAACGATCCTTAGCGGCGGGTGCCAGCCCA
NY_1               TGAAGCAAGAAATTGCGGCTTAACGATCCTTAGCAGCGGGTGCCAGCCCA
Poblonga           TGAAGCAAGAAATTGAGGCTTAACGATCCTTAGCCGCGGGTGCCAGCCCA
Idn2               TGAAGCAAGAAATTGAGGCTTAACGATCCTTAGCGGCGGGTGCCAGCCCA
CJ1_1              TGAAGCAAGAAATTGAGGCTTAACGATCCTTAGCGGCGGGTGCCAGCCCA
CR_1               TGAAGCAAGAAATTGAGGCTTAACGATCCTTAGCGGCGGGTGCCAGCCCA
Pdidermoides       TGAAGCAAGAAATTGAGGCTTAACGATCCTTAGCGGCGGGTGCCAGCCCA
UFF1               TGAAGCAAGAAATTGCGGCTTAACGATCCTTAGCGGCGGGTGCCAGCCCA
CJA3               TGAAGCAAGAAATTGAGGCTTAACGATCCTTAACGGCGGGTGCCAGCCCA
Prigidum           TGAAGCAAGAAATTGCGGCTTAACGATCCTTAGCGGCGGGTGCCAGCCCA
C1                 TGAAGCAAGAAATTGAGGCTTAACGATCCTTAGCGGCGGGTGCCAGCCCA
PpolycephalumWis1  TGAAGCAAGAAATTGCGGCTTAACGATCCTTAGCGGCGGGTGCCAGCCCA
Sflavogenita       TGAAGCGAGAAATCGAGGCTTAACGATCCTTAGCGGCGGGTGCCAGCCCA
```

```
CUR1_4             CGTTTGAGGTGAGAGAAAAGTTACCACAGGGATAACTGGCTTGTGGCCGC
HA4_1              CGTTTGAGGTGAGAGAAAAGTTACCACAGGGATAACTGGCTTGTGGCCGC
CR19_1             CGTTTGAGGTGAGAGAAAAGTTACCACAGGGATAACTGGCTTGTGGCCGC
Pan2               CGTTTGAGGTGAGAGAAAAGTTACCACAGGGATAACTGGCTTGTGGCCGC
CR8_1              CGTTTGAGGTGAGAGAAAAGTTACCACAGGGATAACTGGCTTGTGGCCGC
Fr_K7              CGCTTGAGGTGAGAGAAAAGTTACCACAGGGATAACTGGCTTGTGGCCGC
Fr_K15             CGCTTGAGGTGAGAGAAAAGTTACCACAGGGATAACTGGCTTGTGGCCGC
CR10               CGTTTGAGGTGAGAGAAAAGTTACCACAGGGATAACTGGCTTGTGGCCGC
It_K61             TGCTTGAGGTGAGAGAAAAGTTACCACAGGGATAACTGGCTTGTGGCCGC
Fr_K10             TGCTTGAGGTGAGAGAAAAGTTACCACAGGGATAACTGGCTTGTGGCCGC
It_K66             TGCTTGAGGTGAGAGAAAAGTTACCACAGGGATAACTGGCTTGTGGCCGC
Uk_K79             TGCTTGAGGTGAGAGAAAAGTTACCACAGGGATAACTGGCTTGTGGCCGC
Pr3_1              TGCTTGAGGTGAGAGAAAAGTTACCACAGGGATAACTGGCTTGTGGCCGC
It_K71             CGCTTGAGGTGAGAGAAAAGTTACCACAGGGATAACTGGCTTGTGGCCGC
Fr_K18             CGCTTGAGGTGAGAGAAAAGTTACCACAGGGATAACTGGCTTGTGGCCGC
Fr_K62             CGCTTGAGGTGAGAGAAAAGTTACCACAGGGATAACTGGCTTGTGGCCGC
No_K94             CGTTTGAGGTGAGAGAAAAGTTACCACAGGGATAACTGGCTTGTGGCCGC
Cur_1              CGTTTGAGGTGAGAGAAAAGTTACCACAGGGATAACTGGCTTGTGGCCGC
Az4_1              CGCTTGAGGTGAGAGAAAAGTTACCACAGGGATAACTGGCTTGTGGCCGC
PR_1               CGTTTGAGGTGAGAGAAAAGTTACCACAGGGATAACTGGCTTGTGGCCGC
Butricularis       CGCTTGAGGTGAGAGAAAAGTTACCACAGGGATAACTGGCTTGTGGCCGC
NY_1               CGCTTGAGGTGAGAGAAAAGTTACCACAGGGATAACTGGCTTGTGGCCGC
Poblonga           CGCTTGAGGTGAGAGAAAAGTTACCACAGGGATAACTGGCTTGTGGCCGC
Idn2               CGCTTGAGGTGAGAGAAAAGCTACCACAGGGATAACTGGCTTGTGGCCGC
CJ1_1              CGCTTGAGGTGAGAGAAAAGTTACCACAGGGATAACTGGCTTGTGGCCGC
CR_1               CGCTTGAGGTGAGAGAAAAGTTACCACAGGGAT-ACTGGCTTGTGGCCGC
Pdidermoides       CGCTTGAGGTGAGAGAAAAGTTACCACAGGGATAACTGGCTTGTGGCCGC
UFF1               CGCTTGAGGTGAGAGAAAAGTTACCACAGGGATAACTGGCTTGTGGCCGC
CJA3               CGTTTGAGGTGAGAGAAAAGTTACCACAGGGATAACTGGCTTGTGGCCGC
Prigidum           CGCTTGAGGTGAGAGAAAAGTTACCACAGGGATAACTGGCTTGTGACCGC
C1                 CGCT-----------------------------AACTGGCTTGTGGCTGC
PpolycephalumWis1  CGCTTGAGGTGAGAGAAAAGTTACCACAGGGATAACTGGCTTGTGGCCGC
Sflavogenita       CGCTTGAGGTGAGAGAAAAGTTACCACAGGGATAACTGGCTTGTGGCCGC

CUR1_4             CAAGCGTTCATAGCGACGTGGC
HA4_1              CAAGCGTTCATAGCGACGTGGC
CR19_1             CAAGCGTTCATAGCGACGTGGC
Pan2               CAAGCGTTCATAGCGACGTGGC
CR8_1              CAAGCGTTCATAGCGACGTGGC
Fr_K7              CAAGCGTTCATAGCGACGTGGC
Fr_K15             CAAGCGTTCATAGCGACGTGGC
CR10               CAAGCGTTCATAGCGACGTGGC
It_K61             CAAGCGTTCATAGCGACGTGGC
Fr_K10             CAAGCGTTCACAGCGACGTGGC
It_K66             CAAGCGTTCATAGCGACGTGGC
Uk_K79             CAAGCGTTCATAGCGACGTGGC
Pr3_1              CAAGCGTTCATAGCGACGTGGC
It_K71             CAAGCGTTCATAGCGACGTGGC
Fr_K18             CAAGCGTTCATAGCGACGTGGC
Fr_K62             CAAGCGTTCATAGCGACGTGGC
No_K94             CAAGCGTTCATAGCGACGTGGC
Cur_1              CAAGCGTTA-------------
Az4_1              CAAGCGTTCATAGCGACGTGGC
PR_1               CAAGCGTTCATAGCGACGTGGC
Butricularis       CAAGCGTTCATAGCGACGTGGC
NY_1               CAAGCGTTCATAGCGACGTGGC
Poblonga           CAAGCGTTCATAGCGACGTGGC
Idn2               CAAGCGTTCATAGCGACGTGGC
CJ1_1              CAAGCGTTCATAGCGACGTGGC
CR_1               CAAGCGTTCATAGCGACGTGGC
Pdidermoides       CAAGCGTTCATAGCGACGTGGC
UFF1               CAAGCGTTCATAGCGACGTGGC
CJA3               CAAGCGTTCATAGCGACGTGGC
Prigidum           CAAGCGTTCATAGCGACGTGGC
C1                 CAAGCGTTCATAGCGACGTAGC
PpolycephalumWis1  CAAGCGTTCATAGCGACGTGGC
Sflavogenita       CAAGCGTTCATAGCGACGTGGC
```
