# Supplementary material for: Polyphyletic origin of the genus Physarum (Physarales, Myxomycetes) revealed by nuclear rDNA mini-chromosome analysis and group I intron synapomorphy
Source: BMC Evol Biol. 2012 Aug 31;12:166. doi: 10.1186/1471-2148-12-166 (PMC3511172; doi:10.1186/1471-2148-12-166)
Supplement: Additional file 6 — Figure S3. Alignment of the Didymiaceae LSU data set. [file 1471-2148-12-166-S6.pdf]

# Additional file Figure S3

LSU alignment (765 nt)

**Variable sites:** 200
**Parsimony informative characters:** 159

```
#NEXUS
BEGIN DATA;
dimensions ntax=37 nchar=765;
interleave=yes datatype=DNA gap=-;

matrix
Pan1_66        GAGTAACTATGACTCTCTTAAGGTAGCCAAATGCCTCGTCATTTAATTTG
Pan2           GAGTAACTATGACTCTCTTAAGGTAGCCAAATGCCTCGTCATTTAATTTG
Pan3_3         GAGTAACTATGACTCTCTTAAGGTAGCCAAATGCCTCGTCATTTAATTTG
Hon1_7         GAGTAACTATGACTCTCTTAAGGTAGCCAAATGCCTCGTCATTTAATTTG
CUR1_4         GAGTAACTATGACTCTCTTAAGGTAGCCAAATGCCTCGTCATTTAATTTG
HA4_1          GAGTAACTATGACTCTCTTAAGGTAGCCAAATGCCTCGTCATTTAATTTG
CR19_1         GAGTAACTATGACTCTCTTAAGGTAGCCAAATGCCTCGTCATTTAATTTG
CR8_1          GAGTAACTATGACTCTCTTAAGGTAGCCAAATGCCTCGTCATTTAATTTG
Fr_K7          GAGTAACTATGACTCTCTTAAGGTAGCCAAATGCCTCGTCATTTAATTTG
Fr_K15         GAGTAACTATGACTCTCTTAAGGTAGCCAAATGCCTCGTCAT--AATTTG
It_K64         GAGTAACTATGACTCTCTTAAGGTAGCCAAATGCCTCGTCATTTAATTTG
It_K80         GAGTAACTATGACTCTCTTAAGGTAGCCAAATGCCTCGTCATTTAATTTG
Uk_K77         GAGTAACTATGACTCTCTTAAGGTAGCCAAATGCCTCGTCATTTAATTTG
It_IG45        GAGTAACTATGACTCTCTTAAGGTAGCCAAATGCCTCGTCATTTAATTTG
CR10           GAGTAACTATGACTCTCTTAAGGTAGCCAAATGCCTCGTCATTTAATTTG
It_K61         GAGTAACTATGACTCTCTTAAGGTAGCCAAATGCCTCGTCATTTAATTTG
Uk_K78         GAGTAACTATGACTCTCTTAAGGTAGCCAAATGCCTCGTCATTTAATTTG
Uk_K93         GAGTAACTATGACTCTCTTAAGGTAGCCAAATGCCTCGTCATTTAATTTG
It_IG46        GAGTAACTATGACTCTCTTAAGGTAGCCAAATGCCTCG---TTTAATTTG
Pr3_1          GAGTAACTATGACTCTCTTAAGGTAGCCAAATGCCTCGTCATTTAATTTG
It_K56         GAGTAACTATGACCCTCTTAAGGTAGCCAAATGCCTCGTCATTTAATTTG
Fr_K10         GAGTAACTATGACTCTCTTAAGGTAGCCAAATGCCTCGTCATTTAATTTG
Fr_K12         GAGTAACTATGACTCTCTTAACGTAGCCAAATGCCTCGTCATTTAATTTG
Fr_M26         GAGTAACTATGACTCTCTTAAGGTAGCCAAATGCCTCGTCATTTAATTTG
It_K66         GAGTAACTATGACTCTCTTAAGGTAGCCAAATGCCTCGTCATTTAATTTG
Uk_K79         GAGTAACTATGACTCTCTTAAGGTAGCCAAATGCCTCGTCATTTAATTTG
Mx_K30         GAGTAACTATGACTCTCTTAAGGTAGCCAAATGCCTCGTCATTTAATTTG
It_IG50        GAGTAACTATGACTCTCTTAAGGTAGCCAAATGCCTCGTCATTTAATTTG
It_K68         GAGTAACTATGACTCTCTTAAGGTAGCCAAATGCCTCGTCATTTAATTTG
Uk_K86         GAGTAACTATGACTCTCTTAAGGTAGCCAAATGCCTCGTCATTTAATTTG
It_K52         GAGTAACTATGACTCTCTTAAGGTAGCCAAATGCCTCGTCATTTAATTTG
Fr_K18         GAGTAACTATGACTCTCTTAAGGTAGCCAAATGCCTCGTCATTTAATTTG
It_K71         GAGTAACTATGACTCTCTTAAGGTAGCCAAATGCCTCGTCATTTAATTTG
It_K62         GAGTAACTATGACTCTCTTAAGGTAGCCAAATGCCTCGTCATTTAATTTG
It_K63         GAGTAACTATGACTCTCTTAAGGTAGCCAAATGCCTCGTCATTTAATTTG
No_K94         GAGTAACTATGACTCTCTTAAGGTAGCCAAATGCCTCGTCATTTAATTTG
Ppolycephalum  GAGTAACTATGACTCTCTTAAGGTAGCCAAATGCCTCGTCATTTAATTTG
```

```
Pan1_66        TGACGCGCATG-AATGGATTAAT--AGATTCCCACTGTCCCTACCTACTA
Pan2           TGACGCGCATG-AATGGATTAAT--AGATTCCCACTGTCCCTACCTACTA
Pan3_3         TGACGCGCATG-AATGGATTAAT--AGATTCCCACTGTCCCTACCTACTA
Hon1_7         TGACGCGCATG-AATGGATTAAT--AGATTCCCACTGTCCCTACCTACTA
CUR1_4         TGACGCGCATG-AATGGATTAAT--AGATTCCCACTGTCCCTACCTACTA
HA4_1          TGACGCGCATG-AATGGATTAAT--AGATTCCCACTGTCCCTACCTACTA
CR19_1         TGACGCGCATG-AATGGATTAAT--AGATTCCCACTGTCCCTACCTACTA
CR8_1          TGACGCGCATG-AATGGATTAAT--AGATTCCCACTGTCCCTACCTACTA
Fr_K7          TGACGCGCATG-AATGGATTAAT-GAGATTCCCACTGTCCCTACCTACTA
Fr_K15         TGACGCGCATG-AATGGATTAAT-GAGATTCCCACTGTCCCTACCTACTA
It_K64         TGACGCGCATG-AATGGATTAAT-GAGATTCCCACTGTCCCTACCTACTA
It_K80         TGACGCGCATG-AATGGATTAAT-GAGATTCCCACTGTCCCTACCTACTA
Uk_K77         TGACGCGCATG-AATGGATTAAT-GAGATTCCCACTGTCCCTACCTACTA
It_IG45        TGACGCGCATG-AATGGATTAAT-GAGATTCCCACTGTCCCTACCTACTA
CR10           TGACGCGCATG-AATGGATTAAT-GAGATTCCCACTGTCCCTACCTACTA
It_K61         TGACGCGCATG-AATGGATTAAT-GAGATTCCCACTGTCCCTACCTACTA
Uk_K78         TGACGCGCATG-AATGGATTAAT-GAGATTCCCACTGTCCCTACCTACTA
Uk_K93         TGACGCGCATG-AATGGATTAAT-GAGATTCCCACTGTCCCTACCTACTA
It_IG46        TGACGCGCATG-AATGGATTAAT-GAGATTCCCACTGTCCCTACCTACTA
Pr3_1          TGACGCGCATG-AATGGATTAAT-GAGATTCCCACTGTCCCTACCTACTA
It_K56         TGACGCGCATG-AATGGATTAAT-GAGATTCCCACTGTCCCTACCTACTA
Fr_K10         TGACGCGCATG-AATGGATTAATTGAGATTCCCACTGTCCCTACCTACTA
Fr_K12         TGACGCGCATG-AATGGATTAAT-GAGATTCCCACTGTCCCTACCTACTA
Fr_M26         TGACGCGCATG-AATGGATTAAT-GAGATTCCCACTGTCCCTACCTACTA
It_K66         TGACGCGCATG-AATGGATTAAT-GAGATTCCCACTGTCCCTACCTACTA
Uk_K79         TGACGCGCATG-AATGGATTAAT-GAGATTCCCACTGTCCCTACCTACTA
Mx_K30         TGACGCGCATG-AATGGATTAAT-GAGATTCCCACTGTCCCTACCTACTA
It_IG50        TGACGCGCATG-AATGGATTAAT-GAGATTCCCACTGTCCCTACCTACTA
It_K68         TGACGCGCATG-AATGGATTAAT-GAGATTCCCACTGTCCCTACCTACTA
Uk_K86         TGACGCGCATG-AATGGATTAAT-GAGATTCCCACTGTCCCTACCTACTA
It_K52         TGACGCGCATG-AATGGATTAAT-GAGATTCCCACTGTCCCTACCTACTA
Fr_K18         TGACGCGCATG-AATGGATTAAT-GAGATTCCCACTGTCCCTACCTACTA
It_K71         TGACGCGCATG-AATGGATTAAT-GAGATTCCCACTGTCCCTACCTACTA
It_K62         TGACGCGCATG-AATGGATTAAT-GAGATTCCCACTGTCCCTACCTACTA
It_K63         TGACGCGCATGGAATGGATTAAT-GAGATTCCCACTGTCCCTACCTACTA
No_K94         TGACGCGCATG-AATGGATTAAT-GAGATTCCCACTGTCCCTACCTACTA
Ppolycephalum  TGACGCGCATG-AATGGATTAAT-GAGATTCCCACTGTCCCTACCTACTA
```

```
Pan1_66        TCTAGCGAAACCACAGCCAAGGGAACGGGCTTGGCACAATTAGCGGGGAA
Pan2           TCTAGCGAAACCACAGCCAAGGGAACGGGCTTGGCACAATTAGCGGGGAA
Pan3_3         TCTAGCGAAACCACAGCCAAGGGAACGGGCTTGGCACAATTAGCGGGGAA
Hon1_7         TCTAGCGAAACCACAGCCAAGGGAACGGGCTTGGCACAATTAGCGGGGAA
CUR1_4         TCTAGCGAAACCACAGCCAAGGGAACGGGCTTGGCACAATTAGCGGGGAA
HA4_1          TCTAGCGAAACCACAGCCAAGGGAACGGGCTTGGCACAATTAGCGGGGAA
CR19_1         TCTAGCGAAACCACAGCCAAGGGAACGGGCTTGGCACAATTAGCGGGGAA
CR8_1          TCTAGCGAAACCACAGCCAAGGGAACGGGCTTGGCACAATTAGCGGGGAA
Fr_K7          TCTAGCGAAACCACAGCCAAGGGAACGGGCTTGGCCCAATTAGCGGGGAA
Fr_K15         TCTAGCGAAACCACAGCCAAGGGAACGGGCTTGGCCCAATTAGCGGGGAA
It_K64         TCTAGCGAAACCACAGCCAAGGGAACGGGCTTGGCCCAATTAGCGGGGAA
It_K80         TCTAGCGAAACCACAGCCAAGGGAACGGGCTTGGCCCAATTAGCGGGGAA
Uk_K77         TCTAGCGAAACCACAGCCAAGGGAACGGGCTTGGCCCAATTAGCGGGGAA
It_IG45        TCTAGCGAAACCACAGCCAAGGGAACGGGCTTGGCACAATTAGCGGGGAA
CR10           TCTAGCGAAACCACAGCCAAGGGAACGGGCTTGGCACAATTAGCGGGGAA
It_K61         TCTAGCGAAACCACAGCCAAGGGAACGGGCTTGGCACAATTAGCGGGGAA
Uk_K78         TCTAGCGAAACCACAGCCAAGGGAACGGGCTTGGCACAATTAGCGGGGAA
Uk_K93         TCTAGCGAAACCACAGCCAAGGGAACGGGCTTGGCACAATTAGCGGGGAA
It_IG46        TCTAGCGAAACCACAGCCAAGGGAACGGGCTTGGCACAATTAGCGGGGAA
Pr3_1          TCTAGCGAAACCACAGCCAAGGGAACGGGCTTGGCACAATTAGCGGGGAA
It_K56         TCTAGCGAAACCACAGCCAAGGGAACGGGCTTGGCACAATTAGCGGGGAA
Fr_K10         TCTAGCGAAACCACAGCCAAGGGAACGGGCTTGGCACAATTAGCGGGGAA
Fr_K12         TCTAGCGAAACCACAGCCAAGGGAACGGGCTTGGCACAATTAGCGGGGAA
Fr_M26         TCTAGCGAAACCACAGCCAAGGGAACGGGCTTGGCACAATTAGCGGGGAA
It_K66         TCTAGCGAAACCACAGCCAAGGGAACGGGCTTGGCACAATTAGCGGGGAA
Uk_K79         TCTAGCGAAACCACAGCCAAGGGAACGGGCTTGACACAATTAGCGGGGAA
Mx_K30         TCTAGCGAAACCACAGCCAAGGGAACGGGCTTGGCACAATTAGCGGGGAA
It_IG50        TCTAGCGAAACCACAGCCAAGGGAACGGGCTTGGCACAATTAGCGGGGAA
It_K68         TCTAGCGAAACCACAGCCAAGGGAACGGGCTTGGCACAATTAGCGGGGAA
Uk_K86         TCTAGCGAAACCACAGCCAAGGGAACGGGCTTGGCACAATTAGCGGGGAA
It_K52         TCTAGCGAAACCACAGCCAAGGGAACGGGCTTGGCACAATTAGCGGGGAA
Fr_K18         TCTAGCGAAACCACAGCCAAGGGAACGGGCTTGGCACAATTAGCGGGGAA
It_K71         TCTAGCGAAACCACAGCCAAGGGAACGGGCTTGGCACAATTAGCGGGGAA
It_K62         TCTAGCGAAACCACAGCCAAGGGAACGGGCTTGGCACAATTAGCGGGGAA
It_K63         TCTAGCGAAACCACAGCCAAGGGAACGGGCTTGGTACAATTAGCGGGGAA
No_K94         TYTAGCGAAACCACAGCCAAGGGAACGGGCTTGGCACAATTAGCGGGGAA
Ppolycephalum  TCTAGCGAAACCACAGCCAAGGGAACGGGCTTGGCACAATTAGCGGGGAA
```

```
Pan1_66        AGAAGACCCTGTTGAGCTTGACTCTAGGCACAAACGCGAGGTGATTCTAA
Pan2           AGAAGACCCTGTTGAGCTTGACTCTAGGCACAAACGCGAGGTGATTCTAA
Pan3_3         AGAAGACCCTGTTGAGCTTGACTCTAGGCACAAACGCGAGGTGATTCTAA
Hon1_7         AGAAGACCCTGTTGAGCTTGACTCTAGGCACAAACGCGAGGTGATTCTAA
CUR1_4         AGAAGACCCTGTTGAGCTTGACTCTAGGCACAAACGCGAGGTGATTCTAA
HA4_1          AGAAGACCCTGTTGAGCTGGACTCTAGGCACAAACGCGAGGTGATTCTAA
CR19_1         AGAAGACCCTGTTGAGCTTGACTCTAGGCACAAACGCGAGGTGATTCTAA
CR8_1          AGAAGACCCTGTTGAGCTTGACTCTAGGCACAAACGCGAGGTGATTCTAA
Fr_K7          AGAAGACCCTGTTGAGCTTGACTCTAGGCACAGAAGCGAGGTGATTCTAA
Fr_K15         AGAAGACCCTGTTGAGCTTGACTCTAGGCACAGAAGCGAGGTGATTCTAA
It_K64         AGAAGACCCTGTTGAGCTTGACTCTAGGCACAGAAGCGAGGTGATTCTAA
It_K80         AGAAGACCCTGTTGAGCTTGACTCTAGGCACAGAAGCGAGGTGATTCTAA
Uk_K77         AGAAGACCCTGTTGAGCTTGACTCTAGGCACAGAAGCGAGGTGATTCTAA
It_IG45        AGAAGACCCTGTTGAGCTTGACTCTAGGCACAGAAGCGAGGTGATTCTAA
CR10           AGAAGACCCTGTTGAGCTTGACTCTAGGCACAAAAGCGAGGTGATTCTAA
It_K61         AGAAGACCCTGTTGAGCTTGACTCTAGGCACAGAAGCGAGGTGATTCTAA
Uk_K78         AGAAGACCCTGTTGAGCCTGACTCTAGGCACAGAAGCGAGGTGATTCTAA
Uk_K93         AGAAGACCCTGTTGAGCTTGACTCTAGGCACAGAAGCGAGGTGATTCTAA
It_IG46        AGAAGACCCTGTTGAGCTTGACTCTAGGCACAGAAGCGAGGTGATTCTAA
Pr3_1          AGAAGACCCTGTTGAGCTTGACTCTAGGCACAGAAGCGAGGTGATTCTAA
It_K56         AGAAGACCCTGTTGAGCTTGACTCTAGGCACAGAAGCGAGGTGATTCTAA
Fr_K10         AGAAGACCCT-TTGAGCTTGACTCTAGGCACAGAAGCGAGGTGATTCTAA
Fr_K12         AGAAGACCCTGTTGAGCTTGACTCTAGGCACAGAAGCGAGGTGATTCTAA
Fr_M26         AGAAGACCCTGTTGAGCTTGACTCTAGGCACAGAAGCGAGGTGATTCTAA
It_K66         AGAAGACCCTGTTGAGCTTGACTCTAGGCACAGAAGCGAGGTGATTCTAA
Uk_K79         AGAAGACCCTGTTGAGCTTGACTCTAGGCACAGAAGCGAGGTGATTCTAA
Mx_K30         AGAAGACCCTGTTGAGCTTGACTCTAGGCACAGAAGCGAGGTGATTCTAA
It_IG50        AGAAGACCCTGTTGAGCTTGACTCTAGGCACAGAAGCGAGGTGATTCTAA
It_K68         AGAAGACCCTGTTGAGCTTGACTCTAGGCACAGAAGCGAGGTGATTCTAA
Uk_K86         AGAAGACCCT-TTGAGCTTGACTCTAGGCACAGAAGCGAGGTGATTCTAA
It_K52         AGAAGACCCTGTTGAGCTTGACTCTAGGCACAGAAGCGAGGTGATTCTAA
Fr_K18         AGAAGACCCTGTTGAGCTTGACTCTAGGCACAGAAGCGAGGTGATTCTAA
It_K71         AGAAGACCCTGTTGAGCTTGACTCTAGGCACAGAAGCGAGGTGATTCTAA
It_K62         AGAAGACCCTGTTGAGCTTGACTCTAGGCACAGAAGCGAGGTGATTCTAA
It_K63         AGAAGACCCTGTTGAGCTTGACTCTAGGCACAGAAGCGAGGTGATTCTAA
No_K94         AGAAGACCCTGTTGAGCTTGACTCTAGGCACAAAAGCGAGGTGATTCTAA
Ppolycephalum  AGAAGACCCTGTTGAGCTTGACTCTAGGCATAGACGCGAGGTGATTCTAA
```

```
Pan1_66        AGGTGTAGCATAGGTGGGAGGGCCTG-GCCCGACCTTGAAATACCACCAC
Pan2           AGGTGTAGCATAGGTGGGAGG-CCTG-GCCCGACCTTGAAATACCACCAC
Pan3_3         AGGTGTAGCATAGGTGGGAGGGCCTG-GCCCGACCTTGAAATACCACCAC
Hon1_7         AGGTGTAGCATAGGTGGGAGGGCCTG-GCCCGACCTTGAAATACCACCAC
CUR1_4         AGGTGTAGCATAGGTGGGAGGGCCTG-GCCCGACCTTGAAATACCACCAC
HA4_1          AGGTGTAGCATAGGTGGGAGGGCCTG-GCCCGACCTTGAAATACCACCAC
CR19_1         AGGTGTAGCATAGGTGGGAGGACCTG-GTCCGACCTTGAAATACCACCAC
CR8_1          AGGTGTAGCATAGGTGGGAGG-CCTG-GCCCGACCTTGAAATACCACCAC
Fr_K7          AGGTGTAGCATAGGTGGGAGGGCCTG-GCCCGACCTTGAAATACCACCAC
Fr_K15         AGGTGTAGCATAGGTGGGAGGGCCTG-GCCCGACCTTGAAATACCACCAC
It_K64         AGGTGTAGCATAGGTGGGAGGGCCTG-GCCCGACCTTGAAATACCACCAC
It_K80         AGGTGTAGCATAGGTGGGAGGGCCTG-GCCCGACCCTGAAATACCACCAC
Uk_K77         AGGTGTAGCATAGGTGGGAGGGCCTG-GCCCGACCTTGAAATACCACCAC
It_IG45        AGGTGTAGCATAGGTGGGAGGACCTG-GTCCGACCTTGAAATACCACCAC
CR10           AGGCGTAGCATAGGTGGGAGGACCTG-GTCCGACCCTGAAATACCACCAC
It_K61         AGGTGTAGCATAGGTGGGAGGGCCTG-GCCCGACCTTGAAATACCACCAC
Uk_K78         AGGTGTAGCATAGGTGGGAGGGCCTG-GCCCGACCTTGAAATACCACCAC
Uk_K93         AGGTGTAGCATAGGTGGGAGGGCCTG-GCCCGACCTTGAAATACCACCAC
It_IG46        AGGTGTAGCATAGGTGGGAGGGCCTG-GCCCGACCTTGAAATACCACCAC
Pr3_1          AGGTGTAGCATAGGTGGGAGGGCCTG-GCCCGACCTTGAAATACCACCAC
It_K56         AGGTGTAGCATAGGTGGGAGGGCCTG-GCCCGACCTTGAAATACCACCAC
Fr_K10         AGGTGTAGCATAGGTGGGAGGGCCTG-GCCCGACCTTGAAATACCACCAC
Fr_K12         AGGTGTAGCATAGGTGGGAGGGCCTG-GCCCGACCTTGAAATACCACCAC
Fr_M26         AGGTGTAGCATAGGTGGGAGGGCCTG-GCCCGACCTTGAAATACCACCAC
It_K66         AGGTGTAGCATAGGTGGGAGGGCCTG-GCCCGACCTTGAAATACCACTAC
Uk_K79         AGGTGTAGCATAGGTGGGAGGGCCTG-GCCCGACCTTGAAATACCACCAC
Mx_K30         AGGTGTAGCATAGGTGGGAGGGCCTG-GCCCGACCTTGAAATACCACCAC
It_IG50        AGGTGTAGCATAGGTGGGAGGGCCTG-GCCCGACCTTGAAATACCACCAC
It_K68         AGGTGTAGCATAGGTGGGAGGGCCTG-GCCCGACCTTGAAATACCACCAC
Uk_K86         AGGTGTAGCATAGGTGGGAGGACCTG-GTCCGCCCTTGAAATACCACCAC
It_K52         AGGTGTAGCATAGGTGGGAGGACCCG-GTCCGCCCTTGAAATACCACCAC
Fr_K18         AGGTGTAGCATAGGTGGGAGGACCTG-GTCCGCCCTTGAA-TACCACCAC
It_K71         AGGTGTAGCATAGGTGGGAGGACCTG-GTCCGCCCTTGAAATACCACCAC
It_K62         AGGTGTAGCATAGGTGGGAGGACCTG-GTCCGCCCTTGAAATACCACCAC
It_K63         AGGTGTAGCATAGGTGGGAGGACCTG-GTCCGCCCTTGAAATACCACCAC
No_K94         AGGTGTAGCATAGGTGGGAGGGCCTG-GCCCGACCTTGAAATACCACCAC
Ppolycephalum  AGGTGTAGCATAGGTGGGAGGGCCCATGCCCGTCAATGAAATACCACCAC
```

```
Pan1_66        TTTCGACATTGCCTTGCTAATGCTGTAATAAGTAAACGACCCCCCTTCGT
Pan2           TTTCGACATTGCCTTGCTAATGCTGTAATAAGTAAACGACCCCCCTTCGT
Pan3_3         TTTCGACATTGCCTTGCTAATGCTGTAATAAGTAAACGACCCCCCTTCGT
Hon1_7         TTTCGACATTGCCTTGCTAATGCTGTAATAAGTAAACGACCCCCCTTCGT
CUR1_4         TTTCGACATTGCCTTGCTAATGCTGTAACAAATGGACGGTCCCCCTTCGT
HA4_1          TTTCGACATTGCTTTGCTAATGCTGTAATGAATGAACGACCCCCTATCGT
CR19_1         TTTCGACATTGCCTTGCTAATGCTGTAAAAAGTGAACGGCCCCCCTTCTC
CR8_1          TTTCGACATTGCCTTGCTAATGCTGTAATAAGTAAACGACCCCCCTTCGT
Fr_K7          TTTCGACATCGCTTTGCTAATGCTGTAACGAATGGACGGCCCCCCGATGA
Fr_K15         TTTCGACATCGCTTTGCTAATGCTGTAACGAATGGACGGCCCCCCGATGA
It_K64         TTTCGACATTGCTTTGCTAATGCTGTAACGAATGGACGGCCCCCTGGTGT
It_K80         TTTCGACATCGCTTTGCTAATGCTGTAACGAATGGACGGCCCCCTGATGT
Uk_K77         TTTCGACATCGCTTTGCTAATGCTGTAACGAATGGACGGCCCCCCGATGA
It_IG45        TTTCGACATTACTTTGCTAATGCTGTAACGAATGAACGACCCCTGGGTCG
CR10           TTTCGACATTGCCTTGCTAATGCTGTAACGAATGAACGGCCCCCTGTTTG
It_K61         TTTCGACATCGCTTTGCTAATGCTGTAACGAACGAACGGCCCCCGGGCCC
Uk_K78         TTTCGACATCGCTTTGCTAATGCTGTAACGAACGAACGGCCCCCGGAAGT
Uk_K93         TTTCGACATCGCTTTGCTAATGCTGTAACGAACGAACGGCCCCCGGGCCC
It_IG46        TTGAGACATTGCTTTGCTAATGCTGTAACGAACGAACGGCCCCCGGGCCG
Pr3_1          TTGAGACATTGCTTTGCTAATGCTGTAACGAACGAACGGCCCCCGGGCCG
It_K56         TTTCGACATCGCTTTGCTAATGCTGTAACGAACGAACGGCCCCCGGAAGT
Fr_K10         TTTCGACATCGCTTTGCTAATGCTGTAACGAACGAACGACCCCCAGGACC
Fr_K12         TTTCGACATCGCTTTGCTAATGCTGTAACGAACGAACGGCCCCCGGAAGT
Fr_M26         TTTCGACATCGCTTTGCTAATGCTGTAACGAACGAACGGCCCCCGGGCCC
It_K66         TTTCGACATCGCTTTGCTAATGCTGTAACGAACGAACGACCCCCAGGACC
Uk_K79         TTTCGACATCGCTTTGCTAATGCTGTAACGAACGAACGACCCCCAGGACC
Mx_K30         TTGAGACATTGCTTTGCTAATGCTGTAACGAACGAACGGCCCCCGGTTCC
It_IG50        TTGAGACATTGCTTTGCTAATGCTGTAACGAACGAACGGCCCCCGGGCCG
It_K68         TTTCGACATCGCTTTGCTAATGCTGTAACGAACGAACGGCCCCCGGGCCC
Uk_K86         TTTCGACATTGCTTTGCTAATGCTGTAACGAACGAACGGCCCCCGGGCTG
It_K52         TTTCGACATTGCTTTGCTAATGCTGTAACGAACGAACGGCCCCCGGGCCG
Fr_K18         TTTCGACATTGCTTTGCTAATGCTGTAACGAACGAACGGCCCCCGGGCTG
It_K71         TTTCGACATTGCTTTGCTAATGCTGTAACGAACGAACGGCCCCCGGGCCG
It_K62         TTTCGACATTGCTTTGCTAATGCTGTAACGAACGAACGGCCCCCGGGCCG
It_K63         TTTCGACATTGCTTTGCTAATGCTGTAACGAACGAACGGCCCCCGGGCCG
No_K94         TTTCGACATTGCTTTGCTAATGCTGTAACGAACGAACGGCCCCCTGGACG
Ppolycephalum  TTTCGACATCGCTTTGCTAATGCTGTAACGAACGAACGGAACCGCGTCCC
```

```
Pan1_66        GTCGGCCTTCACGGGTCGGTACGGTCATGTTCTGGCGTTTTCCCTGCTGG
Pan2           GTCGGCCTTCACGGGTCGGTACGGTCATGTTCTGGCGTTTTCCCTGCTGG
Pan3_3         GTCGGCCTTCACGGGTCGGTACGGTCATGTTCTGGCGTTTTCCCTGCTGG
Hon1_7         GTCGGCCTTCACGGGTCGGTACGGTCATGTTCTGGCGTTTTCCCTGCTGG
CUR1_4         GTCGGCTCCTCACGGGGTGTAAGATCATGTTCTGGCGTTTTCCCGGTTCG
HA4_1          GTCGGCTGCCTTCGGGTGGTAAGGTTATATTCTGGATTTTTCCTGGTTTG
CR19_1         GTTGGCTCCTCGCGGGGTGTAAGGCTATGTTCTGGCGTTTTCCTTGCTGG
CR8_1          GTCGGCCTTCACGGGTCGGTACGGTCATGTTCTGGCGTTTTCCCTGCTGG
Fr_K7          CTCGGTTGCCTCCGGGCGGTAAGGCTTCTTTCTGGTGATTTCCCGGTCTG
Fr_K15         CTCGGTTGCCTCCGGGCGGTAAGGCTTCTTTCTGGTGATTTCCCGGTCTG
It_K64         CCCAGCTACCTCCGGGTAGTAAGGCTTCTTTCTAGCGATTTCCCGGTCTG
It_K80         CTCGGCTGCCTCCGGGTGGTAAGGCTTCTTTCTGGTGATCTCCCGGTCTG
Uk_K77         CTCGGCTGCCTCCGGGCGGTAAGGCTTCTTTCTGGTGATTTCCCGGTCCG
It_IG45        CTCGGCTGCTTAACTGTGGTAAGGTCATATTCTAGCGCTATCCTAGCTAG
CR10           TCGTGCCTCACGGTGCGGGTAAGGTCATATTCTGGTGTTTTCCCGGTTGG
It_K61         GTCACCCGCAAGGGTGGCGTAAGGTCATATTCTAGCGCTTTCCTAGTCGG
Uk_K78         TGCCCGTAAGGGTAGTTTGTAAGGTCATATTCTAGCGCTTTCCTAGCGGG
Uk_K93         GTCGCCCGCAAGGGTGGCGTAAGGTCATATTCTAGCGCTTTCCTAGTCGG
It_IG46        CCATCCGCAAGGGTGGCAGTACGGTCATATTCTAGCGCTTTCCTAGTCGA
Pr3_1          CCATCCGCAAGGGTGGCAGTACGGTCATATTCTAGCGCTTTCCTAGTCGA
It_K56         TGCCCGTAAGGGTAGTTTGTAAGGTCATATTCTAGCGCTTTCCTAGCGGG
Fr_K10         GTTGCCCGCAAGGGTGACGTAAGGTCATATTCTAGCGCTTTCCTCGTTGG
Fr_K12         TGCCCGTAACGGTAGTTTGTAAGGTCATATTCTAGCGCTTTCCTTGCGGG
Fr_M26         GCCATCCGCAAGGGTGGCGTAAGGTCATATTCTAGCGCTTTCCTAGTTGG
It_K66         GTTGCCCGCAAGGGTGACGTAAGGTCATATTCTAGCGCTTTCCTCGTTGG
Uk_K79         GTTGCCCGCAAGGGTGACGTAAGGTCATATTCTAGCGCTTTCCTCGTTGG
Mx_K30         CGTCTTCGGGCGGTGCCGGTAAGGTTATATTCTAGCGCTTTCCTAGTCGG
It_IG50        CCATCCGCAAGGGTGGCAGTACGGTCATATTCTAGCGCTTTCCTAGTCGA
It_K68         GTCACCCGCAAGGGTGGCGTAAGGTCATATTCTAGCGCTTTCCTAGTCGG
Uk_K86         TCCAGGCCGCAAGGTCTGGTAAGGTCATATTCTAGCGCTTTCCTGGCTGG
It_K52         CTTGGGCCGCAAGGTTCAGTAAGGTCATATTCTAGCGCTTTCCTGGCTAT
Fr_K18         TTCAGGCCGCAAGGTCTGGTAAGGTCATATTCTAGCGCTTTCCTGGCTGG
It_K71         CTTGGGCCGCAAGGTTCAGTAAGGTCATATTCTAGCGCTTTCCTGGCTAT
It_K62         CTTGGGCCGCAAGGTTCAGTAAGGTCATATTCTAGCGCTTTCCTGGCTAA
It_K63         CTTGGGCCGCAAGGTTCAGTAAGGTCATATTCTAGCGCTTTCCTGGCTGG
No_K94         TCGGTGCCCTCGCGGTATGTAAGGTCATCTTCTAGCGCTTTCCTAGCTAG
Ppolycephalum  TCTCACCGTAAAAGGTGGGGCAATCCAAATTCTAGCGCTTTCCGCGCTCA
```

```
Pan1_66        TAATGATGGCGTGGCTACCTCGGAGAAGTACCCTGAGGCCGCAATCCAGG
Pan2           TAATGATGGCGTGGCTACCTCGGAGAAGTACCCTGAGGCCGCAATCCAGG
Pan3_3         TAATGATGGCGTGGCTACCTCGGAGAAGTACCCTGAGGCCGCAATCCAGG
Hon1_7         TAATGATGGCGTGGCTACCTCGGAGAAGTACCCTGAGGCCGCAATCCAGG
CUR1_4         TCATGATGGCTTGGCTACCCTGGATAAGTACCCTAGGGCCGCAACGTGGG
HA4_1          CCACGATGGTCTGGCTACCCTGGGTGAGTACCCTGGGACCGTGAGTCGGG
CR19_1         TCACGATGGCTTGGCTACCTTGGGTTAGTACCTCGGGGCTACAAGCCGGG
CR8_1          TAATGATGGCGTGGCTACCTCGGAGAAGTACCCTGAGGCCGCAATCCAGG
Fr_K7          ATACGGTGGTTTGGCCCCTCTGGGATAGTACCTCGAGACCATGAGTTGGG
Fr_K15         ATACGGTGGTTTGGCCCCTCTGGGATAGTACCTCGAGACCATGAGTTGGG
It_K64         ATATGATGGCTTGGCCCCTCTGGGTGAGTACTTTGGGACCATGAGTCGGG
It_K80         ACACGGTGGCTTGGCCCCTCTGGGTGAGTACTTCGAGACCATGAGTTGGG
Uk_K77         TCATGATGGTTTGGCCCCTCTGGGATAGTACTTCGAGACCATGAGTTGGG
It_IG45        -TACGACGGTTTTGCCCCTGCGGGATAGTACCTTGGGACCGTGAGTTGGG
CR10           TCATGATGGTCTGGCCCCTCTGGGCGAGTACCCTGGGACCGTGCGTTGGG
It_K61         ATACGATGGCTTGGCTCCTCTGGGTGAGTACCTTGGGACCGCAAACTGGG
Uk_K78         ATACGGTAACTTGGCTCCTCTGGGTGAGTACCTTGGATCCACAAACTGGG
Uk_K93         ATACGATGGCTTGGCTCCTCTGGGTGAGTACTCTGGGACCGCAAACTGGG
It_IG46        GTACGACGGCTTGGCTCCTCTGGGTGAGTACCCTGGGACCGCAAGTTGGG
Pr3_1          GTACGACGGCTTGGCTCCTCTGGGTGAGTACCCTGGGACCGCAAGTTGGG
It_K56         ATACGGTAACTTGGCTCCTCTGGGTGAGTACCTTGGATCCACAAACTGGG
Fr_K10         GTATGATGGTTTGGCTCCTCTGGGATAGTACTTCGAAGCTGCAAACTGGG
Fr_K12         ATACGGTAACTTGGCTCCTCTGGGTGAGTACCTTGGATCCACAAACTGGG
Fr_M26         ATACGGTGGCTTGGCCCCTCTGGGTGAGTACTCTGGGACCGCAACCTGGG
It_K66         GTACGATGGTTTGGCTCCTCTGGGATAGTACTTCGGGACTGCAAACTGGG
Uk_K79         GTACGATGGTTTGGCTCCTCTGGGATAGTACTTCGGGACTGCAAACTGGG
Mx_K30         TCACGACGGCTTGGCTCCTCTGGGATAGTACTCTGAGACCGCGAGCCGGG
It_IG50        GTACGACGGCTTGGCTCCTCTGGGTGAGTACCCTGGGACCGCAAGTTGGG
It_K68         ATACGATGGCTTGGCTCCTCTGGGTGAGTACCTTGGGACCGCAAACTGGG
Uk_K86         TCATGATGGCTTGGCTCCTCTGGGTGAGTACTCTGGGACCGCAAACTGGG
It_K52         ACAAGGTGGCTTGGCTCCTCTGGGTGAGTACTCTGGGACCGCAAACTGGG
Fr_K18         TCATGATGGCTTGGCTCCTCTGGGTGAGTACTCTGGGACCGCAAACTGGG
It_K71         ACAAGGTGGCTTGGCTCCTCTGGGTGAGTACTCTGGGACCGCAAACTGGG
It_K62         TCAAGACGGCTTGGCCCCTCTGGGTGAGTACTCTGGGACCGCAAACTGGG
It_K63         TCAAGATGGCTTGGCCCCTCTGGGTGAGTACTCTGGGACCGCAAACTGGG
No_K94         GTACGGTGGTTTGGCCCCTCTGGGTGAGTACCTTGGGACCACGAGCTGGG
Ppolycephalum  -TACGACGAGTTGGCTCCCCTGGGTGAGTACCGTGGAACCACGAGCTGGG
```

```
Pan1_66        CTTTCGGGCTCGGTGAGCGCTGTTCTCGGGGGAACCGGGGGTTCGTTGTG
Pan2           CTTTCGGGCTCGGTGAGCGCTGTTCTCGGGGGAACCGGGGGTTCGTTGTG
Pan3_3         CTTTCGGGCTCGGTGAGCGCTGTTCTCGGGGGAACCGGGGGTTCGTTGTG
Hon1_7         CTTTCGGGCTCGGTGAGCGCTGTTCTCGGGGGAACCGGGGGTTCGTTGTG
CUR1_4         CTTCCGGGCTCGCAGCGCGTTGTTCTTGGGGGAACTGGGGATC-GCTGGG
HA4_1          CTTCCGAGTCTGGCGAGCGCTGTTCTCGGGGGAACTAGGGCTT-GTTGGG
CR19_1         CTTCCGGGCTCGGTGAGTATTGTTCTCGGGGGAACTGAGGGTCCGCTGGG
CR8_1          CTTTCGGGCTCGGTGAGCGCTGTTCTCGGGGGAACCGGGGGTTCGTTGTG
Fr_K7          CTTCCGAGCCCGGCGAGTGTTG-TTTTGGGGGAACCAGCGACG-GTTGAG
Fr_K15         CTTCCGAGCCCGGCGAGTGTTG-TTTTGGGGGAACCAGCGACG-GTTGAG
It_K64         CTTCCGAGCCTGGCGAGTGCTG-TCTTGGGGGAACCAGAGAAG-GTTGGG
It_K80         CTTCCGAGCCTGGCGATTGCTG-TCTTGGGGGAACCAGCGACG-GTTGAG
Uk_K77         CTTCCGAGCCCGGCGAGTGCTG-TTTTGGGGGAACCAGCGACG-GTTGAG
It_IG45        CTTCCGAGTCCGGCAAACGCTGCTTCTGGGGGAACTGCGGAAG-GTACGA
CR10           CTTCCGAGTCTGACAAACGTTGCTCCCGGGGGAACCAGGGATG-GTTGGA
It_K61         CTTCCGGGTCTGGTGCGCGTTGTTTCCGGGGGAACCAGGGATG-GTTGGG
Uk_K78         CTTCCGGGTCTGGTGCGTGCTGCTTCTGGGGGAACTAGGGATG-GTCGGG
Uk_K93         CTTCCGGGTCTGGTGCGCGTTGTTTCCGGGGGAACCAGGGATG-GTTGGG
It_IG46        CTTCCGGGTCTGACGAGCGTTGCTCCTGGGGGAACCAGGGATA-GTTGGG
Pr3_1          CTTCCGGGTCTGACGAGCGTTGCTCCTGGGGGAACCAGGGATA-GTTGGG
It_K56         CTTCCGGGTCTGGTGCGTGCTGCTTCTGGGGGAACTAGGGATG-GTCGGG
Fr_K10         CTTCCGGGTTCGGTGCGCAT-G-TTTTGGGGGAACCAGGGATG-GTTGGA
Fr_K12         CTTCCGGGTCTGGTGCGTGCTGCTTCTGGGGGAACTAGGGATG-GTCGGG
Fr_M26         CTTCCGGGTCTGGTGCGCGTTGCTTCCGGGGGAACCAGGGATG-GTTGGG
It_K66         CTTCCGGGTTCGGTGCGCATTG-TTCTGGGGGAACCAGGGATG-GTTGGA
Uk_K79         CTTCCGGGTTCGGTGCGCATTG-TTCTGGGGGAACCAGGGATG-GTTGGA
Mx_K30         CTTCCGGGTCTGGTGAGCGTTGCTCTTGGGGGAACCAGGGATA-GTTGGG
It_IG50        CTTCCGGGTCTGACGAGCGTTGCTCCTGGGGGAACCAGGGATA-GTTGGG
It_K68         CTTCCGGGTCTGGTGCGCGTTGTTTCCGGGGGAACCAGGGATG-GTTGGG
Uk_K86         CTTCCGGGTCTGGTGCGCGCTGCTCTTGGGGGAACCAGAGATG-GTTGGG
It_K52         CTTCCGGGTCTGGTGCGCGCTGCTCTCGGGGGAACCAGAGATG-GTTGGG
Fr_K18         CTTCCGGGTCTGGTGCGCGCTGCTCTTGGGGGAACCAGAGATG-GTTGGG
It_K71         CTTCCGGGTCTGGTGCGCGCTGCTCTCGGGGGAACCAGAGATG-GTTGGG
It_K62         CTTCCGGGTCTGGTGCGCGCTGCTCTCGGGGGAACCAGAGATG-GTTGGG
It_K63         CTTCCGGGTCTGGTGCGCGCTGCTCTTGGGGGAACCAGAGATG-GTTGGG
No_K94         CTTACGAGTCTGGTGAGTGTCGCTTCCGGGGGAACCAGGGACG-GTTGGA
Ppolycephalum  CTTCCGAGTCCGGCGAGTGCTGTTTCCGCGGGAACCAGAGACG-GTCTTC
```

```
Pan1_66        CTGTCCTCTGGCCGGCGTAAAGG--GGTATACAAGTC-GTGTAGCAAACT
Pan2           CTGTCCTCTGGCCGGCGTAAAGG--GGTATACAAGTC-GTGTAGCAAACT
Pan3_3         CTGTCCTCTGGCCGGCGTAAAGG--GGTATACAAGTC-GTGTAGCAAACT
Hon1_7         CTGTCCTCTGGCCGGCGTAAAGG--GGTATACAAGTC-GTGTAGCAAACT
CUR1_4         TTGTCCTCTGGCGGGCGTTAACGG-GGTACATAAGTA-GTGTAGCAGACT
HA4_1          CTGTCCTCTGGTGGGCGTTAAATG-GGTACATAAGTC-GTGTAGCAGACT
CR19_1         TTGTCCTCTGGCTGGCTTCGAGG--GGTACACAAGTT-GTGTAGCAAACT
CR8_1          CTGTCCTCTGGCCGGCGTAAAGG--GGTATACAAGTC-GTGTAGCAAACT
Fr_K7          CTGTCCTCTGTCGGACCTCGTAG--GGTACATAAGCC-GTGTAGCAAACC
Fr_K15         CTGTCCTCTGTCGGACCTCGTAG--GGTACATAAGCC-GTGTAGCAAACC
It_K64         CTGTCCTCTGTTGGGCCAACGAAG-GGTACATAAGCC-GTGTAGCAAACC
It_K80         CCGCCCTCTGTCGGACCTCGTAG--GGTACATAAGCC-GTGTAGCAAACC
Uk_K77         CTGCCCTCTGTCGGACCTCGTAG--GGTACATAAGCC-GTGTAGCAAACC
It_IG45        CTGTCCTCTG-CTGGCATCCG----GGCTCATAAGTC-GTGTAGCAGACT
CR10           CTGTCCTCTGTCTGGCCTCGGTAG-GGTACATAAGTT-GTGTAGCAAACC
It_K61         CTGTCCTCTGTCCGGCGAAATCG--GGTACGTAAGCC-GTGTAGCAGACC
Uk_K78         CTGCCCTCTGTCCTGCAAAATCG--GGTACGTAAGCC-GTGTAGCAGACA
Uk_K93         CTGTCCTCTGTCTGGCGAATCG---GGTACGTAAGCC-GTGTAGCAGACC
It_IG46        CTGTCCTCTGCTTGGCGATTCG---GGTACGTAAGCC-GTGTAGCAGACC
Pr3_1          CTGTCCTCTGCTTGGCGATTCG---GGTACGTAAGCC-GTGTANCAGACC
It_K56         CTGCCCTCTGTCCTGCAAAATCG--GGTACGTAAGCC-GTGTAGCAGACA
Fr_K10         CTGTCCTCTGCCTGGCAATTTTG--GGTACGTAAGCC-GTGTAGCAGACT
Fr_K12         CTGCCCTCTGTCCTGCAAAATCG--GGTACGTAAGCC-GTGTAGCAGACA
Fr_M26         CTGTCCTCTGTCTGGCAAGTCG---GGTACGTAAGCC-GTGTAGCAGACC
It_K66         CTGTCCTCTGCCTGGCAATTTTG--GGTACGTAAGCC-GTGTAGCAGACT
Uk_K79         CTGTCCTCTGCCTGGCAATTTTG--GGTACGTAAGCC-GTGTAGCAGACT
Mx_K30         CTGTCCTCTGGCCGGCGTCCT----GGTACGTAAGCC-GTGTAGCAGACC
It_IG50        CTGTCCTCTGCTTGGCGATTCG---GGTACGTAAGCC-GTGTAGCAGACC
It_K68         CTGTCCTCTGTCCGGCGAAATCG--GGTACGTAAGCC-GTGTAGCAGACC
Uk_K86         CTGCCCTCTGTCTGGCGTCAAAATGGGTACGTAAGCC-GTGTAGCAGACA
It_K52         CTGCCCTCTGGATAGCATAAATATGGGTACGTAAGCC-GTGTAGCAGACC
Fr_K18         CTGCCCTCTGTCTGGCGTCAAAATGGGTACGTAAGCC-GTGTAGCAGACA
It_K71         CTGCCCTCTGGATAGCATAAATATGGGTACGTAAGCC-GTGTAGCAGACC
It_K62         CTGCCCTCTGTATAGCATAAATATGGGTACGTAAGCC-GTGTAGCAGACC
It_K63         CTGTCCTCTGTCTGGCATAAATATGGGTACGTAAAGCCGTGTAGCAGACC
No_K94         TCACCCTCTGCCTGGCGTAATCG--GGTACGTTAGCC-GTGTAGCAGACC
Ppolycephalum  TCGGCCTCTG-TGGGCTTCATGCC-GGTACGTAATTGCGTGTAGCAGACT
```

```
Pan1_66        ATTT-GTGTTAGGGAGTTT-GGCTGGGGCGGAAAA-CTGCTACACGGCAA
Pan2           ATTT-GTGTTAGGGAGTTT-GGCTGGGGCGGAAAA-CTGCTACACGGCAA
Pan3_3         ATTT-GTGTTAGGGAGTTT-GGCTGGGGCGGAAAA-CTGCTACACGGCAA
Hon1_7         ATTT-GTGTTAGGGAGTTT-GGCTGGGGCGGAAAA-CTGCTACACGGCAA
CUR1_4         ATTT-GTGTTAGGGAGTTT-GGCTGGGGCGGAAAAACTGCTACACGGCAA
HA4_1          ATTT-GTGTTGGGGAGTTT-GGCTGGGGCGGAAAA-CTGCTACACGGCAA
CR19_1         ATTTTGTGTTAGGGAGTTTTGGCTGGGGCGGAAAA-CTGCTACACGGCAA
CR8_1          ATTT-GTGTTAGGGAGTTT-GGCTGGGGCGGAAAA-CTGCTACACGGCAA
Fr_K7          ATCT-GTGTTGGGGAGTTT-GGCTGGGGCGGAAAA-CTGCTACACGGCAA
Fr_K15         ATCT-GTGTTGGGGAGTTT-GGCTGGGGCGGAAAA-TTGCTACACGGCAA
It_K64         ATCT-GTGTTGGGGAGTTT-GGCTGGGGCGGAAAA-CTGCTACACGGCAA
It_K80         ATCT-GTGTTGGGGAGTTT-GGCTGGGGCGGAAAA-CTGCTACACGGCAA
Uk_K77         ATCT-GTGTTGGGGAGTTT-GGCTGGGGCGGAAAA-CTGCTACACGGCAA
It_IG45        ATCT-GTGTTGGGGAGTTT-GGCTGGGGCGGAAAA-CTGCTACATGGCAA
CR10           ATTT-GTGTTGGGGAGTTT-GGCTGGGGCGGAAAA-CTGCTACACGGCAA
It_K61         ATCT-GTGTTGGGGAGTTT-GGCTGGGGCGGAAAA-CTGCTACACGGCAA
Uk_K78         CTCT-GTGTTGGGGAGTTT-GGCTGGGGCGGAAAA-CTGCTACACGGCAA
Uk_K93         ATCT-GTGTTGGGGAGTTT-GGCTGGGGCGGAAAA-CTGCTACACGGCAA
It_IG46        ATCT-GTGTTGGGGAGTTT-GGCTGGGGCGGAAAA-CTGCTACACGGCAA
Pr3_1          ATCT-GTGTTGGGGAGTTT-GGCTGGGGCGGAAAA-CTGCTACACGGCAA
It_K56         CTCT-GTGTTGGGGAGTTT-GGCTGGGGCGGAAAA-CTGCTACACGGCAA
Fr_K10         ATCT-GTGTTGGGGAGTTT-GGCTGGGGCGGAAAA-CTGCTACACGGCAA
Fr_K12         CTCT-GTGTTGGGGAGTTT-GGCTGGGGCGGAAAA-CTGCTACACGGCAA
Fr_M26         ATCT-GTGTTGGGGAGTTT-GGCTGGGGCGGAAAA-CTGCTACACGGCAA
It_K66         ATCT-GTGTTGGGGAGTTT-GGCTGGGGCGGAAAA-CTGCTACACGGCAA
Uk_K79         ATCT-GTGTTGGGGAGTTT-GGCTGGGGCGGAAAA-CTGCTACACGGCAA
Mx_K30         ATCT-GTGTTGGGGAGTTT-GGCTGGGGCGGAAAA-CTGCTACACGGCAA
It_IG50        ATCT-GTGTTGGGGAGTTT-GGCTGGGGCGGAAAA-CTGCTACACGGCAA
It_K68         ATCT-GTGTTGGGGAGTTT-GGCTGGGGCGGAAAA-CTGCTACACGGCAA
Uk_K86         CTCT-GTGTTGGGGAGTTT-GGCTGGGGCGGAAAA-CTGCTACACGGCAA
It_K52         ATCT-GTGTTGGGGAGTTT-GGCTGGGGCGGAAAC-CTGCTACACGGCAA
Fr_K18         CTCT-GTGTTGGGGAGTTT-GGCTGGGGCGGAAAA-CTGCTACACGGCAA
It_K71         ATCT-GTGTTGGGGAGTTT-GGCTGGGGCGGAAAA-CTGCTACACGGCAA
It_K62         ATCT-GTGTTGGGGAGTTT-GGCTGGGGCGGAAAA-CTGCTACACGGCAA
It_K63         ATCT-GTGTTGGGGAGTTT-GGCTGGGGCGGAAAA-CTGCTACACGGCAA
No_K94         ATTT-GTGTTGGGGAGTTT-GGCTGGGGCGGAAAA-CTGCTACACGGCAA
Ppolycephalum  ATCT-ATGTTGGGGAGTTT-GGCTGGGGCGGAAAA-CTGCTACACGGCAA
```

```
Pan1_66        CGGCAGTCTCCTAAGGTTCACTCAGAGACGACAGAAACGTCTCGTAGAGC
Pan2           CGGCAGTCTCCTAAGGTTCACTCAGAGACGACAGAAACGTCTCGTAGAGC
Pan3_3         CGGCAGTCTCCTAAGGTTCACTCAGAGACGACAGAAACGTCTCGTAGAGC
Hon1_7         CGGCAGTCTCCTAAGGTTCACTCAGAGACGACAGAAACGTCTCGTAGAGC
CUR1_4         CGGCAGTCTCCTAAGGTCCACTCAGAGACGACAGAAACGTCTCGTAGAGC
HA4_1          CGGCAGTCTCCTAAGGTCCACTCAGAGACGACAGAAACGTCTCGTAGAGC
CR19_1         CGGCAGTCTCCTAAGGTCCACTCAGAGACGACAGAAACGTCTCGTAGAGC
CR8_1          CGGCAGTCTCCTAAGGTTCACTCAGAGACGACAGAAACGTCTCGTAGAGC
Fr_K7          CGGCAGTCTCCTAAGGTCCACTCAGAGACGACAGAAACGTCTCGTAGAGC
Fr_K15         CGGCAGTCTCCTAAGGTCCACTCAGAGACGACAGAAACGTCTCGTAGAGC
It_K64         CGGCAGTCTCCTAAGGTCCACTCAGAGACGACAGAAACGTCTCGTAGAGC
It_K80         CGGCAGTCTCCTAAGGTCCACTCAGAGACGACAGAAACGTCTCGTAGAGC
Uk_K77         CGGCAGTCTCCTAAGGTCCACTCAGAGACGACAGAAACGTCTCGTAGAGC
It_IG45        CGGCAGTCTCCTAAGGTCCACTCAGAGACGACAGAAACGTCTCGTAGAGC
CR10           CGGCAGTCTCCTAAGGTCCACTCAGAGACGACAGAAACGTCTCGTAGAGC
It_K61         CGGCAGTCTCCTAAGGTCCACTCAGAGACGACAGAAACGTCTCGTAGAGC
Uk_K78         CGGCAGTCTCCTAAGGTCCACTCAGAGACGACAGAAACGTCTCGTAGAGC
Uk_K93         CGGCAGTCTCCTAAGGTCCACTCAGAGACGACAGAAACGTCTCGTAGAGC
It_IG46        CGGCAGTCTCCTAAGGTCCACTCAGAGACGACAGAAACGTCTCGTAGAGC
Pr3_1          CGGCAGTCTCCTAAGGTCCACTCAGAGACGACAGAAACGTCTCGTAGAGC
It_K56         CGGCAGTCTCCTAAGGTCCACTCAGAGACGACAGAAACGTCTCGTAGAGC
Fr_K10         CGGCAGTCTCCTAAGGTCCACTCAGAGACGACAGAAACGTCTCGTAGAGC
Fr_K12         CGGCAGTCTCCTAAGGTCCACTCAGAGACGACAGAAACGTCTCGTAGAGC
Fr_M26         CGGCAGTCTCCTAAGGTCCACTCAGAGACGACAGAAACGTCTCGTAGAGC
It_K66         CGGCAGTCTCCTAAGGTCCACTCAGAGACGACAGAAACGTCTCGTAGAGC
Uk_K79         CGGCAGTCTCCTAAGGTCCACTCAGAGACGACAGAAACGTCTCGTAGAGC
Mx_K30         CGGCAGTCTCCTAAGGTCCACTCAGAGACGACAGAAACGTCTCGTAGAGC
It_IG50        CGGCAGTCTCCTAAGGTCCACTCAGAGACGACAGAAACGTCTCGTAGAGC
It_K68         CGGCAGTCTCCTAAGGTCCACTCAGAGACGACAGAAACGTCTCGTAGAGC
Uk_K86         CGGCAGTCTCCTAAGGTCCGCTCAGAGACGACAGAAACGCCTCGTAGAGC
It_K52         CGGCAGTCTCCTAAGGTCCGCTCAGAGACGACAGAAACGCCTCGTAGAGC
Fr_K18         CGGCAGTCTCCTAAGGTCCGCTCAGAGACGACAGAAACGCCTCGTAGAGC
It_K71         CGGCAGTCTCCTAAGGTCCGCTCAGAGACGACAGAAACGCCTCGTAGAGC
It_K62         CGGCAGTCTCCTAAGGTCCGCTCAGAGACGACAGAAACGCCTCGTAGAGC
It_K63         CGGCAGTCTCCTAAGGTCCGCTCAGAGACGACAGAAACGCCTCGTAGAGC
No_K94         CGGCAGTCTCC-AAGGTCCACTCAGAGACGACAGAAACGTCTCGTAGAGC
Ppolycephalum  CGGCAGTCTCCTAAGGTCCACTCAGAGACGACAGAAACGTCTCGTAGAGC
```

```
Pan1_66        ATAAGGGCAAAAGTGAGCTTAACTCACATTTTCAGTAGTAATGTGAAGCA
Pan2           ATAAGGGCAAAAGTGAGCTTAACTCACATTTTCAGTAGTAATGTGAAGCA
Pan3_3         ATAAGGGCAAAAGTGAGCTTAACTCACATTTTCAGTAGTAATGTGAAGCA
Hon1_7         ATAAGGGCAAAAGTGAGCTTAACTCACATTTTCAGTAGTAATGTGAAGCA
CUR1_4         ATAAGGGCAAAAGTGGGCTTAACTCACATTTTCAGTAGTAATGTGAAGCA
HA4_1          ATAAAGGCAAAAGTGGGCTTAACTTACATTTTCAGTAGTAATGTGAAGCA
CR19_1         ATAAGGGCAAAAGTGGGCTTAACTTACATTTTCAGTAGTAATGTGAAGCA
CR8_1          ATAAGGGCAAAAGTGAGCTTAACTCACATTTTCAGTAGTAATGTGAAGCA
Fr_K7          ATAAAGGCAAAAGTGGGCTTAACTTACATTTTCAGTAGTAATGTGAAGCA
Fr_K15         ATAAAGGCAAAAGTGGGCTTAACTTACATTTTCAGTAGTAATGTGAAGCA
It_K64         ATAAAGGCAAAAGTGGGCTTAACTTACATTTTCAGTAGTAATGTGAAGCA
It_K80         ATAAAGGCAAAAGTGGGCTTAACTTACATTTTCAGTAGTAATGTGAAGCA
Uk_K77         ATAAAGGCAAAAGTGGGCTTAACTTACATTTTCAGTAGTAATGTGAAGCA
It_IG45        ATAAAGGCAAAAGTGGGCTTAACTCGCATTCTCAGTAGTAATGTGAAGCA
CR10           ATAAAGGCAAAAGTGGGCTTAACTTACATTTTCAGTAGTAATGTGAAGCA
It_K61         ATAAAGGCAAARGTGGGCTTAACTCGCATTTTCAGTAGTAATGTGAAGCA
Uk_K78         ATAAAGGCAAAAGTGGGCTTAACTTACATTTTCAGTAGTAATGTGAAGCA
Uk_K93         ATAAAGGCAAAAGTGGGCTTAACTCGCATTTTCAGTAGTAATGTGAAGCA
It_IG46        ATAAAGGCAAAAGTGGGCTTAACTTACATTCTCAGTAGTAATGTGAAGCA
Pr3_1          ATAAAGGCAAAAGTGGGCTTAACTTACATTCTCAGTAGTAATGTGAAGCA
It_K56         ATAAAGGCAAAAGTGGGCTTAACTTACATTTTCAGTAGTAATGTGAAGCA
Fr_K10         ATAAAGGCAAAAGTGGGCTTAACTCGCATTTTCAGTAGTAATGTGAAGCA
Fr_K12         ATAAAGGCAAAAGTGGGCTTAACTTACATTTTCAGTAGTAATGTGAAGCA
Fr_M26         ATAAAGGCAAAAGTGGGCTTAACTCGCATTTTCAGTAGTAATGTGAAGCA
It_K66         ATAAAGGTAAAAGTGGGCTTAACTCGCATTTTCAGTAGTAATGTGAAGCA
Uk_K79         ATAAAGGCAAA-GTGGGCTTAACTCGCATTTTCAGTAGTAATGTGAAGCA
Mx_K30         ATAAAGGCAAAAGTGGGCTTAACTTACATTCTCAGTAGTAATGTGAAGCA
It_IG50        ATAAAGGCAAAAGTGGGCTTAACTTACATTCTCAGTAGTAATGTGAAGCA
It_K68         ATAAAGGCAAAAGTGGGCTTAACTCGCATTTTCAGTAGTAATGTGAAGCA
Uk_K86         ATAAAGGCAAAAGCGGGCTTAACTTACATTTTCAGTAGTAATGTGAAGCA
It_K52         ATAAAGGCAAAAGCGGGCTTAACTTACATTTTCAGTAGTAATGTGAAGCA
Fr_K18         ATAAAGGCAAAAGCGGGCTTAACTTACATTTTCAGTAGTAATGTGAAGCA
It_K71         ATAAAGGCAAAAGCGGGCTTAACTTACATTTTCAGTAGTAATGTGAAGCA
It_K62         ATAAAGGCAAAAGCGGGCTTAACTTACATTTTCAGTAGTAATGTGAAGCA
It_K63         ATAAAGGCAAAAGCGGGCTTAACTTACATTTTCAGTAGTAATGTGAAGCA
No_K94         ATAAAGGCAAAAGTGGGCTTAACTTACATTCTCAGTAGTAATGTGAAGCA
Ppolycephalum  ATAAAGGCAAAAGTGGGCTTAACTCGCATTTTCAGTAGTAATGTGAAGCA
```

```
Pan1_66        AGAAATTGAGGCCTAACGATCCTTAACGGCGGGTGCCAGCCCACGTTTGA
Pan2           AGAAATTGAGGCCTAACGATCCTTAACGGCGGGTGCCAGCCCACGTTTGA
Pan3_3         AGAAATTGAGGCCTAACGATCCTTAACGGCGGGTGCCAGCCCACGTTTGA
Hon1_7         AGAAATTGAGGCCTAACGATCCTTAACGGCGGGTGCCAGCCCACGTTTGA
CUR1_4         AGAAATTGAGGCCTAACGATCCTTAACGGCGGGTGCCAGCCCACGTTTGA
HA4_1          AGAAATTGAGGCTTAACGATCCTTAACGTCGGGTGCCAGCCCACGTTTGA
CR19_1         AGAAATTGAGGCCTAACGATCCTTAACGGCGGGTGCCAGCCCACGTTTGA
CR8_1          AGAAATTGAGGCCTAACGATCCTTAACGGCGGGTGCCAGCCCACGTTTGA
Fr_K7          AGAAATTGAGGCTTAACGATCCTTAGCGTCGGGTGCCAGCCCGCGCTTGA
Fr_K15         AGAAATTGAGGCTTAACGATCCTTAGCGTCGGGTGCCAGCCCGCGCTTGA
It_K64         AGAAATTGAGGCTTAACGATCCTTAGCGACGGGTGCCAGCCCACGCTTGA
It_K80         AGAAATTGAGGCTTAACGATCCTTAGCGTCGGGTGCCAGCCCGCGCTTGA
Uk_K77         AGAAATTGAGGCTTAACGATCCTTAGCGTCGGGTGCCAGCCCGCGCTTGA
It_IG45        AGAAATTGCGGCTTAACGATCCTTAACGTCGGGTGCCAGCCCACGTTTGA
CR10           AGAAATTGAGGCTTAACGATCCTTAACGTCGGGTGCCAGCCCGCGTTTGA
It_K61         AGAAATTGAGGCTTAACGATCCTTAGCAGCGGGTGCCAGCCCATGCTTGA
Uk_K78         AGAAATTGAGGCTTAACGATCCTTAGCAGCGGGTGCCAGCCCAYGCTTGA
Uk_K93         GGAAATTGAGGCTTAACGATCCTTAGCAGCGGGTGCCAGCCCATGCTTGA
It_IG46        AGAAATTGAGGCTTAACGATCCTTAGCAGCGGGTGCCAGCCCATGCTTGA
Pr3_1          AGAAATTGAGGCTTAACGATCCTTAGCAGCGGGTGCCAGCCCATGCTTGA
It_K56         AGAAATTGAGGCTTAACGATCCTTAGCAGCGGGTGCCAGCCCAYGCTTGA
Fr_K10         AGAAATTGAGGCTTAACGATCCTTAGCAGCGGGTGCCAGCCCATGCTTGA
Fr_K12         AGAAATTGAGGCTTAACGATCCTTAGCAGCGGGTGCCAGCCCATGCTTGA
Fr_M26         AGAAATTGAGGCTTAACGATCCTTAGCAGCGGGTGCCAGCCCATGCTTGA
It_K66         AGAAATTGAGGCTTAACGATCCTTAGCAGCGGGTGCCAGCCCATGCTTGA
Uk_K79         AGAAATTGAGGCTTAACGATCCTTAGCAGCGGGTGCCAGCCCATGCTTGA
Mx_K30         AGAAATTGAGGCTTAACGATCCTTAGCGGCGGGTGCCAGCCCACGCTTGA
It_IG50        AGAAATTGAGGCTTAACGATCCTTAGCAGCGGGTGCCAGCCCATGCTTGA
It_K68         AGAAATTGAGGCTTAACGATCCTTAGCAGCGGGTGCCAGCCCAYGCTTGA
Uk_K86         AGAAATTGAGGCTTAACGATCCTTAGCGTCGGGTGCCAGCCCACGCTTGA
It_K52         AGAAATTGAGGCTTAACGATCCTTAGCGTCGGGTGCCAGCCCACGCTTGA
Fr_K18         AGAAATTGAGGCTTAACGATCCTTAGCGTCGGGTGCCAGCCCACGCTTGA
It_K71         AGAAATTGAGGCTTAACGATCCTTAGCGTCGGGTGCCAGCCCACGCTTGA
It_K62         AGAAATTGAGGCTTAACGATCCTTAGCGTCGGGTGCCAGCCCACGCTTGA
It_K63         AGAAATTGAGGCTTAACGATCCTTAGCGTCGGGTGCCAGCCCACGCTTGA
No_K94         AGAAATTGAGGCTTAACGGTCCTTAACGTCGGGTGCCAGCCCGCGTTTGA
Ppolycephalum  AGAAATTGCGGCTTAACGATCCTTAGCGGCGGGTGCCAGCCCACGCTTGA
```

```
Pan1_66        GGTGAGAGAAAAGTTACCACAGGGATAACTGGCTTGTGGCCGCCAAGCGT
Pan2           GGTGAGAGAAAAGTTACCACAGGGATAACTGGCTTGTGGCCGCCAAGCGT
Pan3_3         GGTGAGAGAAAAGTTACCACAGGGATAACTGGCTTGTGGCCGCCAAGCGT
Hon1_7         GGTGAGAGAAAAGTTACCACAGGGATAACTGGCTTGTGGCCGCCAAGCGT
CUR1_4         GGTGAGAGAAAAGTTACCACAGGGATAACTGGCTTGTGGCCGCCAAGCGT
HA4_1          GGTGAGAGAAAAGTTACCACAGGGATAACTGGCTTGTGGCCGCCAAGCGT
CR19_1         GGTGAGAGAAAAGTTACCACAGGGATAACTGGCTTGTGGCCGCCAAGCGT
CR8_1          GGTGAGAGAAAAGTTACCACAGGGATAACTGGCTTGTGGCCGCCAAGCGT
Fr_K7          GGTGAGAGAAAAGTTACCACAGGGATAACTGGCTTGTGGCCGCCAAGCGT
Fr_K15         GGTGAGAGAAAAGTTACCACAGGGATAACTGGCTTGTGGCCGCCAAGCGT
It_K64         GGTGAGAGAAAAGTTACCACAGGGATAACTGGCTTGTGGCCGCCAAGCGT
It_K80         GGTGAGAGAAAAGT------------AACTGGCTTGTGGCCGCCAAGCGT
Uk_K77         GGTGAGAGAAAAGTTACCACAGGGATAACTGGCTTGTGGCCGCCAAGCGT
It_IG45        GGTGAGAGAAAAGTTACCACAGGGATAACTGGCTTGTGGCCGCCAAGCGT
CR10           GGTGAGAGAAAAGTTACCACAGGGATAACTGGCTTGTGGCCGCCAAGCGT
It_K61         GGTGAGAGAAAAGTTACCACAGGGATAACTGGCTTGTGGCCGCCAAGCGT
Uk_K78         GGTGAGAGAAAAGTTACCACAGGGATAACTGGCTTGTGGCCGCCAAGCGT
Uk_K93         GGTGAGAGAAAAGTTACCACAGGGATAACTGGCTTGTGGCCGCCAAGCGT
It_IG46        GGTGAGAGAAAAGTTACCACAGGGATAACTGGCTTGTGGCCGCCAAGCGT
Pr3_1          GGTGAGAGAAAAGTTACCACAGGGATAACTGGCTTGTGGCCGCCAAGCGT
It_K56         GGTGAGAGAAAAGTTACCACAGGGATAACTGGCTTGTGGCCGCCAAGCGT
Fr_K10         GGTGAGAGAAAAGTTACCACAGGGATAACTGGCTTGTGGCCGCCAAGCGT
Fr_K12         GGTGAGAGAAAAGTTACCACAGGGATAACTGGCTTGTGGCCGCCAAGCGT
Fr_M26         GGTGAGAGAAAAGTTACCACAGGGATAACTGGCTTGTGGCCGCCAAGCGT
It_K66         GGTGAGAGAAAAGTTACCACAGGGATAACTGGCTTGTGGCCGCCAAGCGT
Uk_K79         GGTGAGAGAAAAGTTACCACAGGGATAACTGGCTTGTGGCCGCCAAGCGT
Mx_K30         GGTGAGAGAAAAGTTACCACAGGGATAACTGGCTTGTGGCCGCCAAGCGT
It_IG50        GGTGAGAGAAAAGT------------AACTGGCTTGTGGCCGCCAAGCGT
It_K68         GGTGAGAGAAAAGTTACCACAGGGATAACTGGCTTGTGGCCGCCAAGCGT
Uk_K86         GGTGAGAGAAAAGTTACCACAGGGATAACTGGCTTGTGGCCGCCAAGCGT
It_K52         GGTGAGAGAAAAGTTACCACAGGGATAACTGGCTTGTGGCCGCCAAGCGT
Fr_K18         GGTGAGAGAAAAGTTACCACAGGGATAACTGGCTTGTGGCCGCCAAGCGT
It_K71         GGTGAGAGAAAAGTTACCACAGGGATAACTGGCTTGTGGCCGCCAAGCGT
It_K62         GGTGAGAGAAAAGTTACCACAGGGATAACTGGCTTGTGGCCGCCAAGCGT
It_K63         GGTGAGAGAAAAGTTACCACAGGGATAACTGGCTTGTGGCCGCCAAGCGT
No_K94         GGTGAGAGAAAAGTTACCACAGGGATAACTGGCTTGTGGCCGCCAAGCGT
Ppolycephalum  GGTGAGAGAAAAGTTACCACAGGGATAACTGGCTTGTGGCCGCCAAGCGT
```

```
Pan1_66        TCATAGCGACGTGGC
Pan2           TCATAGCGACGTGGC
Pan3_3         TCATAGCGACGTGGC
Hon1_7         TCATAGCGACGTGGC
CUR1_4         TCATAGCGACGTGGC
HA4_1          TCATAGCGACGTGGC
CR19_1         TCATAGCGACGTGGC
CR8_1          TCATAGCGACGTGGC
Fr_K7          TCATAGCGACGTGGC
Fr_K15         TCATAGCGACGTGGC
It_K64         TCATAGCGACGTGGC
It_K80         TCATAGCGACGTGGC
Uk_K77         TCATAGCGACGTGGC
It_IG45        TCATAGCGACGTGGC
CR10           TCATAGCGACGTGGC
It_K61         TCATAGCGACGTGGC
Uk_K78         TCATAGCGACGTGGC
Uk_K93         TCATAGCGACGTGGC
It_IG46        TCATAGCGACGTGGC
Pr3_1          TCATAGCGACGTGGC
It_K56         TCATAGCGACGTGGC
Fr_K10         TCACAGCGACGTGGC
Fr_K12         TCATAGCGACGTGGC
Fr_M26         TCATAGCGACGTGGC
It_K66         TCATAGCGACGTGGC
Uk_K79         TCATAGCGACGTGGC
Mx_K30         TCATAGCGACGTGGC
It_IG50        TCATAGCGACGTGGC
It_K68         TCATAGCGACGTGGC
Uk_K86         TCATAGCGACGTGGC
It_K52         TCATAGCGACGTGGC
Fr_K18         TCATAGCGACGTGGC
It_K71         TCATAGCGACGTGGC
It_K62         TCATAGCGACGTGGC
It_K63         TCATAGCGACGTGGC
No_K94         TCATAGCGACGTGGC
Ppolycephalum  TCATAGCGACGTGGC
```
