# Supplementary material for: Polyphyletic origin of the genus Physarum (Physarales, Myxomycetes) revealed by nuclear rDNA mini-chromosome analysis and group I intron synapomorphy
Source: BMC Evol Biol. 2012 Aug 31;12:166. doi: 10.1186/1471-2148-12-166 (PMC3511172; doi:10.1186/1471-2148-12-166)
Supplement: Additional file 7 — Figure S4. Alignment of the Physaraceae LSU data set. [file 1471-2148-12-166-S7.pdf]

Additional file Figure S4

LSU alignment (766 nt)

Variable sites: 225
Parsimony informative characters: 162

```
#NEXUS
BEGIN DATA;
dimensions ntax=22 nchar=766;
format missing=?
interleave=yes datatype=DNA gap=-;



matrix
Az4_1          GAGTAACTATGACTCTCTTAAGGTAGCCAAATGCCTCGTCATTTAATTTG
Pr_1           GAGTAACTATGACTCTCTTAAGGTAGCCAAATGCCTCGTCATTTAATTTG
Butricularis   GAGTAACTATGACTCTCTTAAGGTAGCCAAATGCCTCGTCATTTAATTTG
Cur1           GAGTAACTATGACTCTCTTAAGGTAGCCAAATGCCTCGTCATTTAATTTG
It_IG38        GAGTAACTATGACTCTCTTAAGGTAGCCAAATGCCTCGTCATTTAATTTG
IW_1           GAGTAACTATGACTCTCTTAAGGTAGCCAAATGCCTCGTCATTTAATTTG
Mx_K28         GAGTAACTATGACTCTCTTAAGGTAGCCAAATGCCTCGTCATTTAATTTG
NY_1           GAGTAACTATGACTCTCTTAAGGTAGCCAAATGCCTCGTCATTTAATTTG
It_IG39        GAGTAACTATGACTCTCTTAAGGTAGCCAAATGCCTCGTCATTTAATTTG
Poblonga       GAGTAACTATGACTCTCTTAAGGTAGCCAAATGCCTCGTCAT--------
Fr_K2          GAGTAACTATGACTCTCTTAAGGTAGCCAAATGCCTCGTCATTTAATTTG
It_IG42        GAGTAACTATGACTCTCTTAAGGTAGCCAAATGCCTCGTCATTTAATTTG
Idn2           GAGTAACTATGACTCTCTTAAGGTAGCCAAATGCCTCGTCATTTAATTTG
CJ1_1          GAGTAACTATGACTCTCTTAAGGTAGCCAAATGCCTCGTCAT--AATTTG
Cr_1           GAGTAACTATGACTCTCTTAAGGTAGCCAAATGCCTCGTCATTTAATTTG
Pdidermoides   GAGTAACTATGACTCTCTTAAGGTAGCCAAATGCCTCGTCATT-AATTTG
CJA3           GAGTAACTATGACTCTCTTAAGGTAGCCAAATGCCTCGTCATTTAATTTG
Prigidum       GAGTAACTATGACTCTCTTAAGGTAGCCAAATGCCTCGTCATTTAATTTG
C1             GAGTAACTATGACTCTCTTAAGGTAGCCAAATGCCTCGTCAT--AATTTG
UFF1           GAGTAACTATGACTCTCTTAAGGTAGCCAAATGCCTCGTCATTTAATTTG
Ppolycephalum  GAGTAACTATGACTCTCTTAAGGTAGCCAAATGCCTCGTCATTTAATTTG
Diridis        GAGTAACTATGACTCTCTTAAGGTAGCCAAATGCCTCGTCATTTAATTTG

Az4_1          TGACGCGCATGAATGGATTAATGAGATTCCCACTGTCCCTACCTACTATC
Pr_1           TGACGCGCATGAATGGATTAATGAGATTCCCACTGTCCCTACCTACTATC
Butricularis   TGACGCGCATGAATGGATTAATGAGATTCCCACTGTCCCTACCTACTATC
Cur1           TGACGCGCATGAATGGATTAATGAGATTCCCACTGTCCCTACCTACTATC
It_IG38        TGACGCGCATGAATGGATTAATGAGATTCCCACTGTCCCTACCTACTATC
IW_1           TGACGCGCATGAATGGATTAATGAGATTCCCACTGTCCCTACCTACTATC
Mx_K28         TGACGCGCATGAATGGATTAATGAGATTCCCACTGTCCCTACCTACTATC
NY_1           TGACGCGCATGAATGGATTAATGAGATTCCCACTGTCCCTACCTACTATC
It_IG39        TGACGCGCATGAATGGATTAATGAGATTCCCACTGTCCCTACCTACTATC
Poblonga       --ACGCGCATGAATGGATTAATGAGATTCCCACTGTCCCTACCTACTATC
Fr_K2          TGACGCGCATGAATGGATTAATGAGATTCCCACTGTCCCTACCTACTATC
It_IG42        TGACGCGCATGAATGGATTAATGAGATTCCCACTGTCCCTACCTACTATC
Idn2           TGACGCGCATGAATGGATTAATGAGATTCCCACTGTCCCTACCTACTATC
CJ1_1          TGACGCGCATGAATGGATTAATGAGATTCCCACTGTCCCTACCTACTATC
Cr_1           TGACGCGCATGAATGGATTAATGAGATTCCCACTGTCCCTACCTACTATC
Pdidermoides   TGACGCGCATGAATGGATTAATGAGATTCCCACTGTCCCTACCTACTATC
CJA3           TGACGCGCATGAATGGATTAATGAGATTCCCACTGTCCCTACCTACTATC
Prigidum       TGACGCGCATGAATGGATTAATGAGATTCCCACTGTCCCTACCTACTATC
C1             TGACGCGCATGAATGGATTAATGAGATTCCCACTGTCCCTACCTACTATC
UFF1           TGACGCGCATGAATGGATTAATGAGATTCCCACTGTCCCTACCTACTATC
Ppolycephalum  TGACGCGCATGAATGGATTAATGAGATTCCCACTGTCCCTACCTACTATC
Diridis        TGACGCGCATGAATGGATTAATGAGATTCCCACTGTCCCTACCTACTATC
```

```
Az4_1          TAGCGAAACCACAGTCAAGGGAACGGTCTTGACACAATTTGCGGGGAAAG
Pr_1           TAGCGAAACCACAGCCAAGGGAACGGGCTTGGCACAATTAGCGGGGAAAG
Butricularis   TAGCGAAACCACAGCCAAGGGAACGGGCTTGGCACAATTAGCGGGGAAAG
Cur1           TAGCGAAACCACAGCCAAGGGAACGGGCTTGGCACAATTAGCGGGGAAAG
It_IG38        TAGCGAAACCACAGCCAAGGGAACGGGCTTGGCACAATTAGCGGGGAAAG
IW_1           TAGCGAAACCACAGCCAAGGGAACGGGCTTGGCACAATTAGCGGGGAAAG
Mx_K28         TAGCGAAACCACAGCCAAGGGAACGGGCTTGGCACAATTAGCGGGGAAAG
NY_1           TAGCGAAACCACAGCCAAGGGAACGGGCTTGGCACAATTAGCGGGGAAAG
It_IG39        TAGCGAAACCACAGCCAAGGGAACGGGCTTGGCACAATTAGCGGGGAAAG
Poblonga       TAGCGAAACCACAGCCAAGGGAACGGGCTTGGCACAATTAGCGGGGAAAG
Fr_K2          TAGCGAAACCGCAGCCAAGGGAACGGGCTTGGCACAATTAGCGGGGAAAG
It_IG42        TAGCGAAACCACAGCCAAGGGAACGGGCTTGGCACAATTAGCGGGGAAAG
Idn2           TAGCGAAACCACAGCCAAGGGAACGGGCTTGGCACAATTAGCGGGGAAAG
CJ1_1          TAGCGAAACCACAGTCAAGGGAACGGGCTTGACACAATTAGCAGGGAAAG
Cr_1           TAGCGAAACCACAGTCAAGGGAACGGGCTTGACACAATTAGCGGGGAAAG
Pdidermoides   TAGCGAAACCACAGCCAAGGGAACGGGCTTGGCACAATTAGCGGGGAAAG
CJA3           TAGCGAAACCACAGCCAAGGGAACGGGCTTGGCACAATTAGCGGGGAAAG
Prigidum       TAGCGAAACCACAGCCAAGGGAACGGGCTTGGCACAATTAGCGGGGAAAG
C1             TAGCGAAACCACAGCCAAGGGAACGGGCTTGGCACAATTAGCGGGGAAAG
UFF1           TAGCGAAACCACAGCCAAGGGAACGGGCTTGGCACAATTAGCGGGGAAAG
Ppolycephalum  TAGCGAAACCACAGCCAAGGGAACGGGCTTGGCACAATTAGCGGGGAAAG
Diridis        TAGCGAAACCACAGCCAAGGGAACGGGCTTGGCACAATTAGCGGGGAAAG

Az4_1          AAGACCCTGTTGAGCTTGACTCTAGGCATAGACGCGAGGTGATTCTAAAG
Pr_1           AAGACCCTGTTGAGCTTGACTCTAGGCACAGACGCGAGGTGATTCTAAAG
Butricularis   AAGACCCTGTTGAGCTTGACTCTAGGCATAGAAGCGAGGTGATTCTAAAG
Cur1           AAGACCCTGTTGAGCTTGACTCTAGGCACAGACGCGAGGTGATTCTAAAG
It_IG38        AAGACCCTGTTGAGCTTGACTCCAGGCATAGAAGCGAGGTGATTCTAAAG
IW_1           AAGACCCTGTTGAGCTTGACTCTAGGCACAGAAGCGAGGTGATTCTAAAG
Mx_K28         AAGACCCTGTTGAGCTTGACTCTAGGCACAGAAGCGAGGTGATTCTAAAG
NY_1           AAGACCCTGTTGAGCTTGACTCTAGGCACAGAAGCGAGGTGATTCTAAAG
It_IG39        AAGACCCTGTTGAGCTTGACTCTAGGCATAGACGCGAGGTGATTCTAAAG
Poblonga       AAGACCCTGTTGAGCTTGACTCTAGGCACAAACGCGAGGTGATTCTAAAG
Fr_K2          AAGACCCTGTTGAGCTTGACTCTAGGCATAGACGCGAGGTGATTCTAAAG
It_IG42        AAGACCCTGTTGAGCTTGACTCTAGGCATAGAAGCGAGGTGATTCTAAAG
Idn2           AAGACCCTGTTGAGCTTGACTCTAGGCATAGACGCGAGGTGATTCTAAAG
CJ1_1          AAGACCCTGTTGAGCTTGACTCTAGGCATAGACGCGAGGTGATTCTAAAG
Cr_1           AAGACCCTCTTAATCTTGACTCTAGGCATAGACGCCAGGTGATTCTAAAG
Pdidermoides   AAGACCCTGTTGAGCTTGAGTCTAGGCATAGACGCGAGGTGATTCTAAAG
CJA3           AAGACCCTGTTGAGCTTGACTCTAGGCACAGACGCGAGGTGATTCTAAAG
Prigidum       AAGACCCTGTTGAGCTTGACTCTAGGCATAGACGCGAGGTGATTCTAAAG
C1             AAGACCCTGTTGAGCTTGACTCTAGGCACAAACGCGAGGTGATTCTAAAG
UFF1           AAGACCCTGTTGAGCTTGACTCTAGGCATAGAAGCGAGGTGATTCTAAAG
Ppolycephalum  AAGACCCTGTTGAGCTTGACTCTAGGCATAGACGCGAGGTGATTCTAAAG
Diridis        AAGACCCTGTTGAGCTTGACTCTAGGCACAAACGCGAGGTGATTCTAAAG

Az4_1          GTGTAGCATAGGTGGGAGGGCCCAGCCCGATCCTGAAATACCACCACTTT
Pr_1           GTGTAGCATAGGTGGGAGGGCCCAGCCCGACCTTGAAATACCACCACTTT
Butricularis   GTGTAGCATAGGTGGGAGGGTCCAGCCCGACAATGAAATACCACCACTTT
Cur1           GTGTAGCATAGGTGGGAGGGCCCAGCCCGACCTTGAAATACCACCACTTT
It_IG38        GTGTAGCATAGGTGGGAGAACCCAGTTCGACCTTGAAATACCACCACTTT
IW_1           GTGTAGCATAGGTGGGAGGGCCCAGCCCGTCCATGAAATACCACCACTTT
Mx_K28         GTGTAGCATAGGTGGGAGGGCCCAGCCCGTCCATGAAATACCACCACTTT
NY_1           GTGTAGCATAGGTGGGAGGGCCCAGCC-GTCCATGAAATACCACCACTTT
It_IG39        GTGTAGCATAGGTGGGAGGGCCTAGCCCGACCTTGAAATACCACCACTTT
Poblonga       GTGTAGCATAGGTGGGAGGGCCCAGCCCGACCTTGAAATACCACCACTTT
Fr_K2          GTGTAGCATAGGTGGGAGGGCCCAGCCCGATCCTGAAATACCACCACTTT
It_IG42        GTGTAGCATAGGTGGGAGGGCACAGCCCGACCTTGAAATACCACCACTTT
Idn2           GTGTAGCATAGGTGGGAGGGCCCAGCCCGATCCTGAAATACCACCACTTT
CJ1_1          GTGTAGCATAGGTGGGAGGGCCCAGCCCGATCCTGAAATACCACCACTTT
Cr_1           GTGTAGCATAGGTGGGAGGGCCCAGCCCGATCCTGAAATACCACCACTTT
Pdidermoides   GTGTAGCATAGGTGGGAGGGCCCGATCC----CTGAAATACCACCACTTT
CJA3           GTGTAGCATAGGTGGGAGGGCCCAGCCCGACCTTGAAATACCACCACTTT
Prigidum       GTGTAGCATAGGTGGGAGGGCCCCGCCCGTCCATGAAATACCACCACTTT
C1             GTGTAGCATAGGTGGGAGGGCACAGCCCGACCTTGAAATACCACCACTTT
UFF1           GTGTAGCATAGGTGGGAGAGCACAGCTCGCCAATGAAATACCACCACTTT
Ppolycephalum  GTGTAGCATAGGTGGGAGGGCCCAGCCCGTCAATGAAATACCACCACTTT
Diridis        GTGTAGCATAGGTGGGAG-GCCTGGCCCGACCTTGAAATACCACCACTTT
```

```
Az4_1          CGACATCGCTTTGCTAATGCTGTAACGAATGAACGACCCC-CCTTCT-GA
Pr_1           CGACATCGCTTTGCTAATGCTGTAACGAACGAACGGCCCCTCGGTGTCCC
Butricularis   CGACATCGCTTTGCTAATGCTGTAACGAACGAACGGAACCGCGTCCCTCT
Cur1           CGACATCGCTTTGCTAATGCTGTAACGAACGAACGGCCCCTCGGTGTCCC
It_IG38        CGACATTGCTTTGCTAATGCTGTAACGAATGAACGGCCCC-CGGCACCCT
IW_1           CGACTTTGCTTTGCTAATGCTGTAACGAACGAACGACACCCAAGGGGCTT
Mx_K28         CGGCATTGCTTTGCTAATGCTGTAACGAACGAACGACACCCAAGGGGCTT
NY_1           CGACATTGCTTTGCTAATGCTGTAACGAACGAACGACACCCAAGGG-CTT
It_IG39        CGACATTGCTTTGCCAATGCTGTAACGAATGAACGGCCCCTCGGTACTCG
Poblonga       AGACATTGCTTTGCTAATGCTGTAACGAATGAACGGCCCCCTGATATCCT
Fr_K2          CGACATCGCTTTGCTAATGCTGTAACGAATGAACGATCCCCCGTTCCAGA
It_IG42        CGACATTGCTTTGCTAACGCTGTAACGAATGAACGGCCCCCTAGTCTCCC
Idn2           CGACATCGCTTTGCTAATGCTGTAACGAATGAACGATCCCCCGTTCCAGA
CJ1_1          CGACATCGCTTTGCTAATGCTGTAACGAATGAACGACCCC-CCTTCT-GA
Cr_1           CGACATCGCTTTGCTAATGCTGTAACGAATGAACGACCCC-CGTTCT-GA
Pdidermoides   CGACATCGCTTTGCTAATGCTGTAACGAATGAACGACCCCTCGTCCT-GA
CJA3           CGACATCGCTTTGCTAATGCTGTAACGAACGAACGACCCCTCGGTGTCCC
Prigidum       CGACATCGCTTTGCTAATGCTGTAACGAACGAACGGAACCGCGTCCCTCT
C1             CGACATTGCTTTGCTAATGCTGTAACGAACAAACGGCCCCCTGGTTCCCC
UFF1           CGATATTGCTTTGCTAATGCTGTAACGAACGAACGCAACCGCGTCCCTCT
Ppolycephalum  CGACATCGCTTTGCTAATGCTGTAACGAACGAACGGAACCGCGTCCCTCT
Diridis        CGACATTGCCTTGCTAATGCTGTAATAAGTAAACGACCCCCCTTCGTGTC

Az4_1          AGCCC-GCAAGGGTGGAAGCTGGGTAAGGTCATATTCTGGCGTCGTCCTC
Pr_1           GCTCG--AAAGGGCGGGCGCCGGGTAAGGTCACATTCTAGCGCTTTCCCG
Butricularis   CGTCG--AAAGGCGGGGGAAGGGATAGGCCCAAATTCTAGCGCTTTCCGC
Cur1           GCTCG--AAAGGGCGGGCGCCGGGTAAGGTCACATTCTAGCGCTTTCCCG
It_IG38        AGTCG--CAAGATTAG--GACCGGTAAGGTCATATTCTGGCGCTTTCCGA
IW_1           -----------TACGCACCTAGGGTAAG-TCATATTCTAGCGTTTTCCAG
Mx_K28         -----------TACGCACCTAGGGTAAG-TCATATTCTAGCGTTTTCCAG
NY_1           -----------TACGCACCTAGGGTAAG-TCATATTCTAGCGTTTTCCAG
It_IG39        GCTCG--AAAGGGTCGTTGCTGGGTAAGGTCATATTCTGGCGATTTCCGA
Poblonga       GCTCG--GAAGGGTGGGTATTGGGTAAGGTCATATTCTAGTGCTTTCCGA
Fr_K2          GGTCC-GCAAGGGCTGAAGTCGGGTAAGATCATATTCTGGCGTCTTCCAC
It_IG42        GACCG--AAAGAA-AGGTACTGGGTAAGGTCATTTTCTAGCATTTTCCGT
Idn2           GGTCC-GCAAGGGCTGAAGTCGGGTAAGATCATATTCTGGCGTCTTCCAC
CJ1_1          AGCCC-GCAAGGGTGGAAGCTGGGTAAGGTCATATTCTGGCGTCGTCCTC
Cr_1           AGCC--GCAAGG-TGGAAGCTCGGTAAGGTCATATTCTGGCGTCGTCCTC
Pdidermoides   AGCTC-GCAAGAGTGGAAGTCGGGTAAGGTCAGATTCTGGTGTCCTCCAT
CJA3           GCTCG--AAAGGGCGGGCGCCGGGTAAGGTCACATTCTAGCGCTTTCCCG
Prigidum       CGTCG--CAAGATGGGGGAAGGGATAGGCCCAAATTCTAGCGCTTTCCAC
C1             GTCCG--CAAGGGCGGGTGCCGGGTAAGGTCATATTCTAGCGTTTTCCGG
UFF1           CGTCG--AAAGACGGGGGAAGGGATAGGCGCAAATTCTAGCGCTTTCCGC
Ppolycephalum  CACCGTAAAAGGTGGGGGAAGGGATAGGCCCAAATTCTAGCGCTTTCCGC
Diridis        GGCCTTCACGGGTCGGCTGTTGGGTACGGTCATGTTCTGGCGTTTTCCCT

Az4_1          GATCGCATGACGAGTCGGCTACCCTGGGATAGTACTTCNAATCCGTAATT
Pr_1           ACTCGTACGGTGGCCTGGCCCCTCCGGGTGAGTACCTCGTGACCACAAAC
Butricularis   GCTCGCATGACGGATCGGCTCCTCTGGGTGAGTACTGTGGGACCGCGAGT
Cur1           ACTCGTACGGTGGCCTGGCCCCTCCGGGTGAGTACCTCGTGACCACAAAC
It_IG38        GTCCAAATGATAGGTCGGACCCCCTAGGTGAGTACTTCGGAACCGCAAAT
IW_1           GCACGTACGATGGTCAAACCCCTCTGGGTGAGTACCTCGAGACCACAAAC
Mx_K28         GCACGTACGATGGTCAAACCCCTCTGGGTGAGTACCTCGAGACCACAAAC
NY_1           GCACGT-CGATGGGTCAAACCCTCTGGGTGAGTACCTCGAGACCACAAAC
It_IG39        GCTCACATGGTGGGTCGGACCCTCTGGGTGAGTACTTCGGGACCACAAAC
Poblonga       GCTCGTATGATGGCTTGGGCTCTCTGGGATAGTACTTTGGGACCGCAAAC
Fr_K2          GGTTGCATGATGGGTTAGCTAACCTAGGATAGTACTTCGGATCCACGAGA
It_IG42        GT-CATACGGCGGCTTGGCCCCTCTGGGTGAGTACTTTGGGACCACAAAC
Idn2           GGTTGCATGATGGGTTAGCTAACCTAGGATAGTACTTCGGATCCACGAGA
CJ1_1          GATCGCATGACGAGTCGGCTACCCTGGGATAGTACTTCGAATCCGTAATT
Cr_1           GATCGTACGACGGGTTGGCTACCCTGGGATAGTACTTCGAATCCGCAATT
Pdidermoides   AATCGTACGATTGGTTGGCTAACCTAGGATAGTACTTCGAGTCCGTGAGC
CJA3           ACTCGTACGGTGGCCTGGCCCCTCCGGGTGAGTACCTCGTGACCACAAAC
Prigidum       GCTCTCATGATGTGGCGGCTCCCCTGGGTGAGTACCGCGAGACCGTACGC
C1             GTTCACACGATGGCTTGGCTCCTCTGGGTGAGTACTCTGGGACCACAAAC
UFF1           GCCCGCATGACGGATTGGCTCCCCTGGGTGAGTACTGCGAGACCGTAAGT
Ppolycephalum  GCTCATACGACGAGTTGGCTCCCCTGGGTGAGTACCGTGGAACCACGAGC
Diridis        GCTGGTATGATGGCGTGGCTACCTCGGAGAAGTACCCTGAGGCCGCAATC
```

```
Az4_1          CGGGCTTCCGGGTCT-GGAGAGCGCTGTGTTTGGGGGAACTAGGTGTGA-
Pr_1           TGGGCCTCCGGGTCT-GGTGCGTGTCGTTCTCGGGGGAACCGGAGACGG-
Butricularis   CGGGCTTCCGAGTCT-GGCGAGTGCTGCTTCTGCGGGAACCAGGGACGG-
Cur1           TGGGCCTCCGGGTCT-GGTGCGTGTCGTTCTCGGGGGAACCGGAGACGG-
It_IG38        TGGGCTTCCGGGTCT-GATGCGCGTCGATTTTGGGGGAACTAGGGATGA-
IW_1           TGGGCTTCCGGGTCT-GGTGCGTGTTGCTCTTGGGGGAACTGGAGGTCG-
Mx_K28         TGGGCTTCCGGGTCT-GGTGCGTGTTGCTCTTGGGGGAACTGGAGGTCG-
NY_1           TGGGCTTCCGGGTCTTGGTGCGTGTTGCTCTTGGGG-AACTGGAGGTCG-
It_IG39        TGGGCTTCCGGGTCT-GGTGCGTGTTGATTCTGGGGGAACCAGAGACGG-
Poblonga       TGGGCTTCCGGGTTC-GGTGCGTGTTGTTTCTGAGGGAACCAGAGATCGC
Fr_K2          CGGGCTTCCGGGTCT-GGCGAATGTTGCGTTCGGGGGAACTAGGCGTGA-
It_IG42        TGGGCTTCCGGGTCT-GGTGCGTGCTGCTCCTGGGGGAACTAGGGATGG-
Idn2           CGGGCTTCCGGGTCT-GGCGAGTGTTGCGTTCGGGGGAACTAGGCGTGA-
CJ1_1          CGGGCTTCCGGGTCT-GGAGAGCGCTGTGTTTGGGGGAACTAGGTGTGA-
Cr_1           CGGGCTTCCGGGTCT-GGAGCGCGCTGTGTTTGGGGGAACCAGGTGTGA-
Pdidermoides   TGGGCTTCCGGGTCT-GGCAAGCGTTGTGCTTGGGGGAACTAGGCGTNA-
CJA3           TGGGCCTCCGGGTCT-GGTGCGTGTCGTTCTCGGGGGAACCGGAGACGG-
Prigidum       TGAGCTTCCGAGCCC-GGTAAACGCTGTCCTCGTGGGAACCAGAGACGG-
C1             TGGGCCTCCGGGTTC-GGTGCGTGCTGCTTCTGGGGGAACCAGGGATGG-
UFF1           TGGGCTTCCGAGTCC-GGCGCGCGTTGTTCTTGCGGGAACCAGGGACGGG
Ppolycephalum  TGGGCTTCCGAGTCC-GGCGAGTGCTGTTTCCGCGGGAACCAGAGACGG-
Diridis        CAGGCTTTCGGGCTC-GGTGAGCGCTGTTCTCGGGGGAACCGGGGGTTCG

Az4_1          TGGCTGCGTCCTCTGCGGTCTTTATT--GGTACATAGTCA---GTAGCAG
Pr_1           TTGGGCCGCCCTCTGCGGGCTAAAAG--GGTACGTAAGCC-GTGTAGCAA
Butricularis   TCGAACTGTCCTCTGCGGGCTTCAAGCCGGTACGTAAT-GCGTGTAGCAG
Cur1           TTGGGCCGCCCTCTGCGGGCTAAAAG--GGTACGTAAGCC-GTGTAGCAA
It_IG38        TTGACCCGTCCTCTGGGACACT------GGTACATAA-GCCGTGTAGCAA
IW_1           TGCGACTGTCCTCTGCGGGCATTAAACTGGTACGTAATCC-GTGTAGCAG
Mx_K28         TGCGACTGTCCTCTGCGGGCATTAAACTGGTACGTAATCC-GTGTAGCAG
NY_1           TGCGACTGTCCTCTGCGGGCATTAAACTGGTACGTAATCC-GTGTAGCAG
It_IG39        CTGACTCGTCCTCTGGGGCAAAT-----GGTACATAA-GTCGTGTAGCAG
Poblonga       TTGGGCTGTCCTCTATGGGGTCAAGGATGGTACATAAAGTCGTGTAGCAG
Fr_K2          GAGC-TCATCCTCTGCAGCCAAAAT---GGTACATAGTCA---GTAGCAG
It_IG42        TTGGGTTGTCCTCTGTGGCAAAAATT--GGTACATAA-GCCGTGTAGCGG
Idn2           GAGC-TCATCCTCTGCAGCCAAAAT---GGTACATAGTCA---GTAGCAG
CJ1_1          TGGC-GCGTCCTCTGCGGTCTTTATT--GGTACATAGTCA---GTAGCAG
Cr_1           TAAC-ACGTCCTCTGCGGTCTTTATT--GGTACATAGTCA---GTAGCAG
Pdidermoides   TGACTG-GTCCTCTGCGGTTTAATATT-GGTACATAGTCA---GTAGCAG
CJA3           TTGGGCTGCCCTCTGCGGGCTAAAAG--GGTACGTAAGCCCGTGTAGCAA
Prigidum       TCGTCGTGTCCTCTGAGGGCTTCAAGTTGGTACGTAAG-CCGTGTAGCAG
C1             TTGGGCTGTCCTCTGTGGGCGAATATC-GGTATGTAA-GCCGTGTAGCAG
UFF1           TCGAACCGTCCTCTGTGGGCTTTAAATGGGTACGTAATTGCGTGTAGCAG
Ppolycephalum  TCTTCTCGGCCTCTGTGGGCTTCATGCCGGTACGTAATTGCGTGTAGCAG
Diridis        TTGTGCTGTCCTCTGGCCGGCGTAAAGGGGTATACAAGTC-GTGTAGCAA

Az4_1          ACTATCTATGTTGGGGAGTTTGGCTGGGGCGGAAAACTGCTACACGGCAA
Pr_1           ACCATCTGTGTTGGGGAGTTTGGCTGGGGCGGAAAACTGCTACACGGCAA
Butricularis   ACTATCTATGTTGGGGAGTTTGGCTGGGGCGGAAAACTGCTACACGGCAA
Cur1           ACCATCTGTGTTGGGGAGTTTGGCTGGGGCGGAAAACTGCTACACGGCAA
It_IG38        ACCATCTATGTTGGGGAGTTTGGCTGGGGCGGAAAACTGCTACACGGCAA
IW_1           ACACTCTGTGTTGGGGAGTTTGGCTGGGGCGGAAAACTGCTACACGGCAA
Mx_K28         ACACTCTGTGTTGGGGAGTTTGGCTGGGGCGGAAAACTGCTACACGGCAA
NY_1           ACACTCTGTGTTGGGGAGTTTGGCTGGGGCGGAAAACTGCTACACGGCAA
It_IG39        ACTATCTATGTTGGGGAGTTTGGCTGGGGCGGAAAACTGCTACACGGCAA
Poblonga       ACTCTTTGTGTTAGGGAGTTTGGCTGGGGCGGAAAACTGCTACATGGCAA
Fr_K2          ACTATCTATGTTGGGGAGTTTGGCTGGGGCGGAAAACTGCTACACGGCAA
It_IG42        ACCATCTATGTTGGGGAGTTTGGCTGGGGCGGAAAACTGCTACACGGCAA
Idn2           GCTATCTATGTTGGGGAGTTTGGCTGGGGCGGAAACCTACCACACGGCAA
CJ1_1          ACTATCTATGTTGGGGAGTTTGGCTGGGGCGGAAAACTGCTACACGGCAA
Cr_1           ACTATCTATGTTGGGGAGTTTGGCTGGGGCGGAAAACTGCTACACGGCAA
Pdidermoides   ACTATCTATGTTGGGGAGTTTGGCTGGGGCGGAAAACTGCTACACGGCAA
CJA3           ACCATCTGTGTTGGGGAGTTTGGCTGGGGCGGAAAACTGCTACACGGCAA
Prigidum       ACTATCTATGTTGGGGAGTTTGGCTGGGGCGGAAAACTGCTACACGGCAA
C1             ACCATTTGTGTTGGGGAGTTTGGCTGGGGCGGAAAACTGCTACACGGCAA
UFF1           ACTATCTATGTTGGGGAGTTTGGCTGGGGCGGAAAACTGCTACACGGCAA
Ppolycephalum  ACTATCTATGTTGGGGAGTTTGGCTGGGGCGGAAAACTGCTACACGGCAA
Diridis        ACTATTTGTGTTAGGGAGTTTGGCTGGGGCGGAAAACTGCTACACGGCAA
```

```
Az4_1          CGGCAGTCTCCTAAGGTCCACTCAGAGACGACAGAAACGTCTCGTAGAGC
Pr_1           CGGCAGTCTCCTAAGGTCCACTCAGAGACGACAGAAACGTCTCGTAGAGC
Butricularis   CGGCAGTCTCCTAAGGTCCACTCAGAGACGACAGAAACGTCTCGTAGAGC
Cur1           CGGCAGTCTCCTAAGGTCCACTCAGAGACGACAGAAACGTCTCGTAGAGC
It_IG38        CGGCAGTCTCTTAAGGTTCACTCAGCGACGACAGAAACGCCGCGTAGAGC
IW_1           CGGCAGTCTCCTAAGGTCCACTCAGAGACGACAGAAACGTCTCGTAGAGC
Mx_K28         CGGCAGTCTCCTAAGGTCCACTCAGAGACGACAGAAACGTCTCGTAGAGC
NY_1           CGGCAGTCTCCTAAGGTCCACTCAGAGACGACAGAAACGTCTCGTAGAGC
It_IG39        CGGCAGTCTCCTAAGGTCCACTCAGAGACGACAGAAACGTCTCGTAGAGC
Poblonga       CGGCAGTCTCCTAAGGTCCACTCAGAGACGACAGAAAC-TCTCGTAGAGC
Fr_K2          CGGCAGTCTCCTAAGGTCCACTCAGAGACGACAGAAACGTCTCGTAGAGC
It_IG42        CGGCAGTCTCCTAAGGTCCACTCAGAGACGACAGAAACGTCTCGTAGAGC
Idn2           CGGCAGTCTCCTAAGGTCCACTCAGAGACGACAGAAACGTCTCGTAGAGC
CJ1_1          CGGCAGTCTCCTAAGGTCCACTCAGAGACGACAGAAACGTCTCGTAGAGC
Cr_1           CGGCAGTCTCCTAAGGTCCACTCAGAGACGACAGAAACGTCTCGTAGAGC
Pdidermoides   CGGCAGTCTCCTAAGGTCCACTCAGAGACGACAGAAACGTCTCGTAGAGC
CJA3           CGGCAGTCTCCTAAGGTCCACTCAGAGACGACAGAAACGTCTCGTAGAGC
Prigidum       CGGCAGTCTCCTAAGGTCCACTCAGAGACGACAGAAACGTCTCGTAGAGC
C1             CGGCAGTCTCCTAAGGTCCACTCAGAGACGACAGAAACGTCTCGTAGAGC
UFF1           CGGCAGTCTCCTAAGGTCCGCTCAGAGACGACAGAAACGTCTCGTAGAGC
Ppolycephalum  CGGCAGTCTCCTAAGGTCCACTCAGAGACGACAGAAACGTCTCGTAGAGC
Diridis        CGGCAGTCTCCTAAGGTTCACTCAGAGACGACAGAAACGTCTCGTAGAGC

Az4_1          ATAAAGGCAAAAGTGGGCTTAACTCACGTTTTCA-GTAGTAATGTGAAGC
Pr_1           ATAAAGGCAAAAGTGGGCTTANGTCGCATTTTCA-GTAGTAATGTGAAGC
Butricularis   ATAAAGGCAAAAGTGGGCTTAACTCGCATTTTCAAGTAGTAATGTGAAGC
Cur1           ATAAAGGCAAAAGTGGGCTTAACTCGCATTTTCA-GTAGTAATGTGAAGC
It_IG38        ATAAAGGCAAAAGTGAGCTTAACTTACATTTTCA-GTAGTAATGTGAAGC
IW_1           ATAAAGGCAAAAGTGGGCTTAACTCACGTTTTCA-GTAGTAATGTGAAGC
Mx_K28         ATAAAGGCAAAAGTGGGCTTAACTCACGTTTTCA-GTAGTAATGTGAAGC
NY_1           ATAAAGGCAAAAGTGGGCTTAACTCACGTTTTCA-GTAGTAATGTGAAGC
It_IG39        ATAAAGGCAAAAGTGGGCTTAACTCGCATTTTCA-GTAGTAATGTGAAGC
Poblonga       ATAAAGGCAAAAGTGGGCTTAACTCGCATTTT-A-GTAGTAATGTGAAGC
Fr_K2          ATAAAGGCAAAAGTGGGCTTAACTCGCATTTTCA-GTAGTAATGTGAAGC
It_IG42        ATAAAGGCAAAAGTGGGCTTAACTYRCATTTTCA-GTAGTAATGTGAAGC
Idn2           ATAAAGGCAAAAGTGGGCTTAACTCACGTTTTCA-GTAGTAATGTGAAGC
CJ1_1          ATAAAGGCAAAAGTGGGCTTAACTCACGTTTTCA-GTAGTAATGTGAAGC
Cr_1           ATAAAGGCAAAAGTGGGCTTAACTCACGTTTTCA-GTAGTAATGTGAAGC
Pdidermoides   ATAAAGGCAAAAGTGGGCTTAACTCACGTTTTCA-GTAGTAATGTGAAGC
CJA3           ATAAAGGCAAAAGTGGGCTTAACTCGCATTTTCA-GTAGTAATGTGAAGC
Prigidum       ATAAAGGCAAAAGTGGGCTTAACTCGCATTTTCA-GTAGTAATGTGAAGC
C1             ATAAAGGNAAAAGTGGGCTTAACTCGCATTTTCA-GTAGTAATGTGAAGC
UFF1           ATAAAGGCAAAAGCGGGCTTAACTCGCATTTTCA-GTAGTAATGTGAAGC
Ppolycephalum  ATAAAGGCAAAAGTGGGCTTAACTCGCATTTTCA-GTAGTAATGTGAAGC
Diridis        ATAAGGGCAAAAGTGAGCTTAACTCACATTTTCA-GTAGTAATGTGAAGC

Az4_1          AAGAAATTGAGGCTTAACGATCCTTAGCGGCGGGTGCCAGCCCACGCTTG
Pr_1           AAGAAATTGAGGCTTAACGATCCTTAACGGCGGGTGCCAGCCCACGTTTG
Butricularis   AAGAAATTGCGGCTTAACGATCCTTAGCGGCGGGTGCCAGCCCACGCTTG
Cur1           AAGAAATTGAGGCTTAACGATCCTTAACGGCGGGTGCCAGCCCACGTTTG
It_IG38        AAGAAATTGAGGCTTAACGATCCTTAGCGGCGGGTGCCAGCCCACGCTTG
IW_1           AAGAAATTGCGGCTTAACGATCCTTAGCAGCGGGTGCCAGCCCATGCTTG
Mx_K28         AAGAAATTGCGGCTTAACGATCCTTAGCAGCGGGTGCCAGCCCAYGCTTG
NY_1           AAGAAATTGCGGCTTAACGATCCTTAGCAGCGGGTGCCAGCCCACGCTTG
It_IG39        AAGAAATTGAGGCTTAACGATCCTTAGCGGCGGGTGCCAGCCCACGCTTG
Poblonga       AAGAAATTGAGGCTTAACGATCCTTAGCCGCGGGTGCCAGCCCACGCTTG
Fr_K2          GAGAAATTGCGGCTTAACGATCCTTAGCGGCGGGTGCCAGCCCACGCTTG
It_IG42        AAGAAATTGMGGCTTAACGATCCTTAGCGGCGGGTGCCAGCCCACGCTTG
Idn2           AAGAAATTGAGGCTTAACGATCCTTAGCGGCGGGTGCCAGCCCACGCTTG
CJ1_1          AAGAAATTGAGGCTTAACGATCCTTAGCGGCGGGTGCCAGCCCACGCTTG
Cr_1           AAGAAATTGAGGCTTAACGATCCTTAGCGGCGGGTGCCAGCCCACGCTTG
Pdidermoides   AAGAAATTGAGGCTTAACGATCCTTAGCGGCGGGTGCCAGCCCACGCTTG
CJA3           AAGAAATTGAGGCTTAACGATCCTTAACGGCGGGTGCCAGCCCACGTTTG
Prigidum       AAGAAATTGCGGCTTAACGATCCTTAGCGGCGGGTGCCAGCCCACGCTTG
C1             AAGAAATTGAGGCTTAACGATCCTTAGCGGCGGGTGCCAGCCCACGCT--
UFF1           AAGAAATTGCGGCTTAACGATCCTTAGCGGCGGGTGCCAGCCCACGCTTG
Ppolycephalum  AAGAAATTGCGGCTTAACGATCCTTAGCGGCGGGTGCCAGCCCACGCTTG
Diridis        AAGAAATTGAGGCCTAACGATCCTTAACGGCGGGTGCCAGCCCACGTTTG
```

```
Az4_1          AGGTGAGAGAAAAGTTACCACAGGGATAACTGGCTTGTGGCCGCCAAGCG
Pr_1           AGGTGAGAGAAAAGTTACCACAGGGATAACTGGCTTGTGGCCGCCAAGCG
Butricularis   AGGTGAGAGAAAAGTTACCACAGGGATAACTGGCTTGTGGCCGCCAAGCG
Cur1           AGGTGAGAGAAAAGTTACCACAGGGATAACTGGCTTGTGGCCGCCAAGCG
It_IG38        AGGTGAGAGAAAAGTTACCACAGGGATAACTGGCTTGTGGCCGCCAAGCG
IW_1           AGGTGAGAGAAAAGTTACCACAGGGATAACTGGCTTGTGGCCGCCAAGCG
Mx_K28         AGGTGAGAGAAAAGTTACCACAGGGATAACTGGCTTGTGGCCGCCAAGCG
NY_1           AGGTGAGAGAAAAGTTACCACAGGGATAACTGGCTTGTGGCCGCCAAGCG
It_IG39        AGGTGAGAGAAAAGTTACCACAGGGATAACTGGCTTGTGGCCGCCAAGCG
Poblonga       AGGTGAGAGAAAAGTTACCACAGGGATAACTGGCTTGTGGCCGCCAAGCG
Fr_K2          AGGTGAGAGAAAAGTTACCACAGGGATAACTGGCTTGTGGCCGCCAAGCG
It_IG42        AGGTGAGAGAAAAGTTACCACAGGGATAACTGGCTTGTGGCCGCCAAGCG
Idn2           AGGTGAGAGAAAAGCTACCACAGGGATAACTGGCTTGTGGCCGCCAAGCG
CJ1_1          AGGTGAGAGAAAAGTTACCACAGGGATAACTGGCTTGTGGCCGCCAAGCG
Cr_1           AGGTGAGAGAAAAGTTACCACAGGGAT-ACTGGCTTGTGGCCGCCAAGCG
Pdidermoides   AGGTGAGAGAAAAGTTACCACAGGGATAACTGGCTTGTGGCCGCCAAGCG
CJA3           AGGTGAGAGAAAAGTTACCACAGGGATAACTGGCTTGTGGCCGCCAAGCG
Prigidum       AGGTGAGAGAAAAGTTACCACAGGGATAACTGGCTTGTGACCGCCAAGCG
C1             ---------------------------AACTGGCTTGTGGCTGCCAAGCG
UFF1           AGGTGAGAGAAAAGTTACCACAGGGATAACTGGCTTGTGGCCGCCAAGCG
Ppolycephalum  AGGTGAGAGAAAAGTTACCACAGGGATAACTGGCTTGTGGCCGCCAAGCG
Diridis        AGGTGAGAGAAAAGTTACCACAGGGATAACTGGCTTGTGGCCGCCAAGCG

Az4_1          TTCATAGCGACGTGGC
Pr_1           TTCATAGCGACGTGGC
Butricularis   TTCATAGCGACGTGGC
Cur1           TTCATAGCGACGTGGC
It_IG38        TTCATAGCGACGTGGC
IW_1           TTCATAGCGACGTGGC
Mx_K28         TTCATAGCGACGTGGC
NY_1           TTCATAGCGACGTGGC
It_IG39        TTCATAGCGACGTGGC
Poblonga       TTCATAGCGACGTGGC
Fr_K2          TTCATAGCGACGTGGC
It_IG42        TTCATAGCGACGTGGC
Idn2           TTCATAGCGACGTGGC
CJ1_1          TTCATAGCGACGTGGC
Cr_1           TTCATAGCGACGTGGC
Pdidermoides   TTCATAGCGACGTGGC
CJA3           TTCATAGCGACGTGGC
Prigidum       TTCATAGCGACGTGGC
C1             TTCATAGCGACGTAGC
UFF1           TTCATAGCGACGTGGC
Ppolycephalum  TTCATAGCGACGTGGC
Diridis        TTCATAGCGACGTGGC
```
