# Supplementary material for: Polyphyletic origin of the genus Physarum (Physarales, Myxomycetes) revealed by nuclear rDNA mini-chromosome analysis and group I intron synapomorphy
Source: BMC Evol Biol. 2012 Aug 31;12:166. doi: 10.1186/1471-2148-12-166 (PMC3511172; doi:10.1186/1471-2148-12-166)
Supplement: Additional file 8 — Table S4. Geographic origin, classification and culturing of the myxomycete isolates. [file 1471-2148-12-166-S8.pdf]

TABLE S4. Origin of myxomycete isolates and culture methods

Species	Isolate	Comment
Didymium		
D. clavus	It-IG45	Collected as sporocarps; classified by I and GM; DNA extracted directly from the sporocarps
D. dubium	Fr-K7	Collected as sporocarps; classified according to fruiting body morphology; cell-line obtained; cultivated and DNA extracted according to [12]
D. dubium	Fr-K15	Collected as sporocarps; classified according to fruiting body morphology; cell-line obtained; cultivated and DNA extracted according to [12]
D. dubium	It-K64	Collected as sporocarps; classified according to fruiting body morphology; DNA extracted directly from sporocarps
D. dubium	Uk-K80	Collected as sporocarps; classified according to fruiting body morphology; DNA extracted directly from sporocarps
D. dubium	Uk-K77	Collected as sporocarps; classified according to fruiting body morphology; DNA extracted directly from sporocarps
D. iridis	Pan1-66	Collected and classified by JC; cultivated according to [38]; DNA extracted according to [12]
D. iridis	Pan2	Cultivated according to [38]; DNA extracted according to [11]
D. iridis	Pan3-3	Collected and classified by JC; cultivated according to [38]; DNA extracted according to [12]
D. iridis	Hon1-7	Cultivated according to [38]; DNA extracted according to [11]
D. iridis	CUR1-4	Collected and classified by JC; cultivated according to [38]; DNA extracted according to [12]
D. iridis	HA4-1	Collected and classified by JC; cultivated according to [38]; DNA extracted according to [12]
D. iridis	CR19-1	Collected and classified by JC; cultivated according to [38]; DNA extracted according to [12]
D. iridis	CR8-1	Collected and classified by JC; cultivated according to [38]; DNA extracted according to [12]
D. squamulosum	Cr10	Collected and classified by JC; cultivated according to [38]; DNA extracted according to [12]
Diderma		
D. meyerae	It-K61	Collected as sporocarps; classified according to fruiting body morphology; DNA extracted directly from sporocarps
D. microcarpum	Uk-K93	Collected as sporocarps; classified according to fruiting body morphology; DNA extracted directly from sporocarps
D. niveum	Fr-K10	Collected as sporocarps; classified according to fruiting body morphology; cell-line obtained; cultivated and DNA extracted according to [12]
D. niveum	Fr-M26	Collected as sporocarps; classified by MM; DNA extracted directly from the sporocarps
D. niveum	It-K66	Collected as sporocarps; classified according to fruiting body morphology; DNA extracted directly from sporocarps
D. niveum	Uk-K79	Collected as sporocarps; classified according to fruiting body morphology; DNA extracted directly from sporocarps
D. saondersii	Mx-K30	Collected as sporocarps; classified according to fruiting body morphology; DNA extracted directly from sporocarps
D. testaceum	It-IG50	Collected as sporocarps; classified by I and GM; DNA extracted directly from the sporocarps
Diderma sp.	It-K68	Collected as sporocarps; classified according to fruiting body morphology; DNA extracted directly from sporocarps
Diderma sp.	Fr-K12	Collected as sporocarps; classified according to fruiting body morphology; DNA extracted directly from sporocarps
Diderma sp.	It-K56	Collected as sporocarps; classified according to fruiting body morphology; DNA extracted directly from sporocarps
Diderma sp.	Uk-K78	Collected as sporocarps; classified according to fruiting body morphology; DNA extracted directly from sporocarps
Diderma sp.	It-IG46	Collected as sporocarps; classified by I and GM; DNA extracted directly from the sporocarps
Diderma sp.[(a)]	Pr3-1	Collected and classified by JC; cultivated according to [38]; DNA extracted according to [12]
Lepidoderma		
L. aggregatum	Uk-K86	Collected as sporocarps; classified according to fruiting body morphology; DNA extracted directly from sporocarps
L. carestianum	Fr-K18	Collected as sporocarps; classified according to fruiting body morphology; cell-line obtained; cultivated and DNA extracted according to [12]
L. carestianum	It-K71	Collected as sporocarps; classified according to fruiting body morphology; DNA extracted directly from sporocarps
L. crustaceum	It-K62	Collected as sporocarps; classified according to fruiting body morphology; DNA extracted directly from sporocarps
L. peyerimhoffii	It-K63	Collected as sporocarps; classified according to fruiting body morphology; DNA extracted directly from sporocarps
Lepidoderma sp.	It-K52	Collected as sporocarps; classified according to fruiting body morphology; DNA extracted directly from sporocarps
Mucilago		
M. crustacea	No-K94	Collected as sporocarps; classified according to fruiting body morphology; DNA extracted directly from sporocarps
Badhamia		
B. melanospora	Az4-1	Collected and classified by JC; cultivated according to [38]; DNA extracted according to [12]
B. melanospora	Pr-1	Collected and classified by JC; cultivated according to [38]; DNA extracted according to [12]
B. utricularis	-- [(b)]	Collected and classified by EFH; cultivated according to [38]; DNA extracted according to [12]

Physaracea-like sp.	Cur1	Collected by JC; cultivated according to [38]; DNA extracted according to [12]
Craterium		
C. minutum	It-IG38	Collected as sporocarps; classified by I and GM; DNA extracted directly from the sporocarps
Fuligo		
F. septica	IW-1	Collected as sporocarps; classified by PH; DNA extracted directly from the sporocarps
F. septica	Mx-K28	Collected as sporocarps; classified according to fruiting body morphology; DNA extracted directly from sporocarps
F. septica	NY-1	Collected as sporocarps; classified according to fruiting body morphology; DNA extracted directly from sporocarps
Leocarpus		
L. fragilis	It-IG39	Collected as sporocarps; classified by I and GM; DNA extracted directly from the sporocarps
Physarella		
P. oblonga	--	Collected and classified by EFH; cultivated according to [38]; DNA extracted according to [12]
Physarum		
P. albescens	Fr-K2	Collected as sporocarps; classified according to fruiting body morphology; DNA extracted directly from sporocarps
P. bivalve	It-IG42	Collected as sporocarps; classified by I and GM; DNA extracted directly from the sporocarps
P. cinereum	Idn-2	Collected and classified by JC; cultivated according to [38]; DNA extracted according to [12]
P. compressum	CJ1-1	Collected and classified by JC; cultivated according to [38]; DNA extracted according to [12]
P. compressum	Cr-1	Collected and classified by JC; cultivated according to [38]; DNA extracted according to [12]
P. didermoides	--	Collected and classified by EFH; cultivated according to [38]; DNA extracted according to [12]
P. flavicomum	UFF1	Collected and classified by GS; cultivated according to [38]; DNA extracted according to [12]
P. polycephalum	Wis1	Cultivated according to [38]; DNA extracted according to [11]
P. pusillum	CJA3	Collected and classified by JC; cultivated according to [38]; DNA extracted according to [12]
P. rigidum	ATCC22485	Collected and classified by EFH; cultivated according to [38]; DNA extracted according to [12]
P. roseum	C1	Collected and classified by JC; cultivated according to [38]; DNA extracted according to [12]
MYCETOZOA		
**Myxomycetes** (plasmodial slime molds)		
**Stemonitales (Order)**		
Stemonites flavogenita	ATCC24714	Collected and classified by EFH; cultivated according to [38]; DNA extracted according to [12]
**Echinosteliales (Order)**		
Echinostelium minutum	ATCC22345	Collected and classified by EFH; cultivated according to [38]; DNA extracted according to [12]
**Protostelids**		
Soliformovum irregulare	ATCC26826	Collected and classified by EFH; cultivated according to [38]; DNA extracted according to [12]
**Dictyostelids** (cellular slime molds)		
Acytostelium ellipticum	ATCC22247	Collected and classified by EFH; cultivated according to [38]; DNA extracted according to [12]

Notes:

Collectors: I and GM, Iolantha and Giovanni Manavella (Italy); JC, Jim Clark (University of Kentucky, USA); MM, Marianne Meyer (France); EFH, Edward F. Haskins (University of Washington, USA); PH, Peik Haugen (University of Tromsø, Norway); GS, Greg Shipley (University of Texas, USA).

Geographic regions of isolates: Fr, French Alps; It, Italian maritime Alps; Mx, Mexico; Uk, Western Ukraine; No, Northern Norway; NY, New York State, USA; IW, Iowa State, USA; Us, USA.

[(a)] Reported previous as *Didymium anellus* in [11,12]. [(b)] --, no isolate name given
